# ﻿New species in old mountains: integrative taxonomy reveals ten new species and extensive short-range endemism in *Nesticus* spiders (Araneae, Nesticidae) from the southern Appalachian Mountains

**DOI:** 10.3897/zookeys.1145.96724

**Published:** 2023-02-03

**Authors:** Marshal Hedin, Marc A. Milne

**Affiliations:** 1 Department of Biology, San Diego State University, San Diego, California 92182–4614, USA San Diego State University San Diego United States of America; 2 Department of Biology, University of Indianapolis, Indianapolis, Indiana 46227, USA University of Indianapolis Indianapolis United States of America

**Keywords:** Cryophilic, invertebrate conservation, mitochondrial introgression, montane speciation, short range endemism, ultraconserved elements

## Abstract

This revision is based on sampling efforts over the past three decades in the southern Appalachian Mountains which have provided *Nesticus* (Araneae, Nesticidae) collections of approximately 2100 adult specimens from more than 475 unique collecting events. Using a “morphology first” framework we examined recently collected specimens plus museum material to formulate morphology-based species hypotheses for putative new taxa (discovery phase). Using sequence capture of nuclear ultraconserved elements (UCEs) we analyzed 801 nuclear loci to validate new (and prior) morphology-based species hypotheses (validation phase) and reconstructed a robust backbone phylogeny including all described and new species. Sanger sequencing and UCE-bycatch were also used to gather mitochondrial data for more than 240 specimens. Based on our integrative taxonomic framework ten new *Nesticus* species are herein described, including *N.binfordae***sp. nov.**, *N.bondi***sp. nov.**, *N.canei***sp. nov.**, *N.cherokeensis***sp. nov.**, *N.dellingeri***sp. nov.**, *N.dykemanae***sp. nov.**, *N.jemisinae***sp. nov.**, *N.lowderi***sp. nov.**, *N.roanensis***sp. nov.**, and *N.templetoni***sp. nov.** Previously unknown males are also described for *N.bishopi* Gertsch, 1984, *N.crosbyi* Gertsch, 1984, and *N.silvanus* Gertsch, 1984, as well as the previously unknown female for *N.mimus* Gertsch, 1984. Based on combined evidence *N.cooperi* Gertsch, 1984 is placed in synonymy with *N.reclusus* Gertsch, 1984. Overall, the montane radiation of Appalachian *Nesticus* reveals a general lack of species sympatry and compelling biogeographic patterns. Several regional *Nesticus* taxa are rare, microendemic habitat specialists that deserve conservation attention and detailed future monitoring as conservation sentinels.

## ﻿Introduction

Systematists and evolutionary biologists have long been interested in mountains. Mountains function as habitat islands, serve as refugia in the face of climatic variation, and generate ecological gradients (Wollenberg et al. 2008; Starrett et al. 2018; Rahbek et al. 2019; Perrigo et al. 2020). These combinations of isolation and selective forces act as engines for the origin and persistence of species diversity. In North America, the several physiographic provinces that together comprise the southern Appalachian Mountains represent an ancient and biodiverse region (Stein et al. 2000; Niemiller and Zigler 2013). A combination of climatic variability and long-term habitat availability, in concert with high topographic complexity, has promoted species diversification. For example, endemic radiations of upland arthropod taxa are found in millipedes (Marek and Bond 2006, 2009; Marek 2010; Means et al. 2021; Hennen et al. 2022), harvestmen (Thomas and Hedin 2008; Hedin and Thomas 2010; Hedin and McCormack 2017; Derkarabetian et al. 2022), spiders (Hendrixson and Bond 2005; Keith and Hedin 2012; Hedin et al. 2015; Newton et al. 2020), and beetles (Sokolov et al. 2004; Caterino and Langton-Myers 2019).

**Figure 1.**
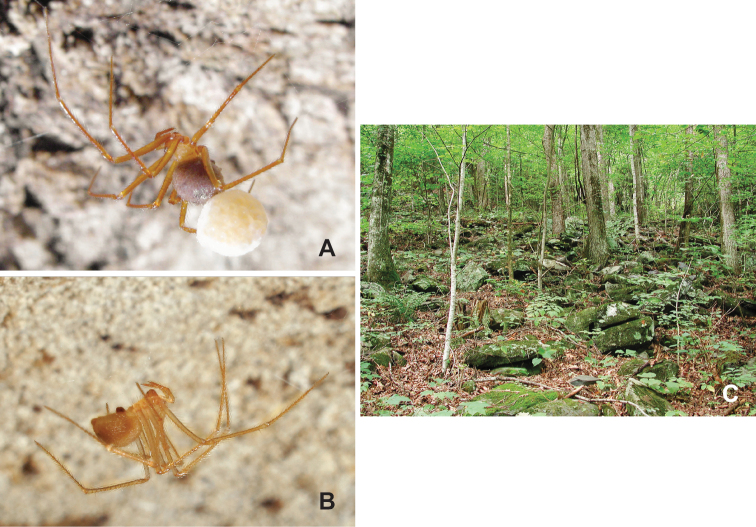
**A** ♀ *Nesticusstupkai* with egg sac, Tennessee, Sevier Co., Wear Cove **B** ♀ *N.barri*, Alabama, Jackson Co., Horseskull Cave **C** montane boulderfield habitat, North Carolina, Macon Co., S of Wayah Bald, MCH 02_169.

The spider genus *Nesticus* Thorell, 1869 (family Nesticidae) is taxonomically diverse in southern Appalachia, with 28 described species distributed over a geographic area extending from southern West Virginia to central Alabama (Gertsch 1984; Coyle and McGarity 1992; Hedin 1997a; Hedin and Dellinger 2005; Zigler and Milne 2022). Appalachian *Nesticus* are habitat specialists with apparently strict physiological constraints that limit these spiders to dark, cool and moist microhabitats (Fig. 1A–C). Suitable microhabitats include limestone caves (at lower elevations), higher-elevation fissure caves, void spaces in north-facing rock fields, and deep north-facing litter. Appalachian *Nesticus* must be specifically targeted for collecting and are uncommon in general collections. Recent updates to the revisionary work of Gertsch (1984) have focused on cave-dwelling, highly troglomorphic *Nesticus* from the Appalachian Valley and Ridge and Cumberland Plateau geologic provinces. This fauna includes several species known only from single localities (Hedin and Dellinger 2005; Carver et al. 2016; Zigler and Milne 2022), making these taxa susceptible to population decline and perhaps extinction.

This revision focuses more specifically, but not exclusively, on montane Appalachian *Nesticus*, particularly taxa from the mountains of northern Georgia, western North Carolina, northeastern Tennessee, and southwestern Virginia. Both Gertsch (1984) and Coyle and McGarity (1992) described *Nesticus* species from this region but based taxonomic conclusions on small sample sizes often from widely separated geographic locations. For previously described taxa, much denser sampling is needed to fully understand geographic distributions, patterns of geographic variation, and species interactions at geographic boundaries. In addition, because of habitat specificity and high regional topographic complexity, denser geographic sampling is expected to result in the discovery of new montane microendemic species.

In his 1984 revision of North American nesticid spiders, Gertsch (1984) argued that his treatment of Appalachian *Nesticus* was preliminary. In particular, Gertsch predicted that additional geographic and specimen sampling would likely increase regional species diversity and knowledge of geographic distributions, and possibly alter species limits hypotheses. Here we combine morphological data from more than 2100 specimens, original ultraconserved element (UCE) DNA sequence data for 95 specimens, and Sanger / UCE-bycatch mitochondrial data for 241 specimens. Most of these specimens are derived from *Nesticus*-devoted collecting efforts from the past 25 years. We take a “morphology first” taxonomic approach, formulating species hypotheses based on study of male and female genitalia, then independently test these hypotheses using genomic-scale nuclear data bolstered by mitochondrial evidence. Overall, this research reveals a remarkable radiation of short-range endemic (*sensu*Harvey et al. 2011), mostly parapatric *Nesticus* species, including several new species which are rare in suitable habitats and deserve conservation attention.

## ﻿Materials and methods

### ﻿Specimen and geographic sampling

We acknowledge that the land upon which we searched for and collected specimens is the traditional and ancestral territories of the Calicuas, Cheraw, Chickasaw, Eno, Kaskaskia, Keyauwee, Lumbee, Manahoac, Miccosukee, Monacan, Moneton, Mvskoke (Muscogee), Myaamia, Occaneechi, Osage, Pee Dee, Saponi, S’atsoyaha (Yuchi), Shakori, Shawandasse Tula (Shawanwaki / Shawnee), Sissipahaw, Skaruhreh / Tuscarora, Sugaree, Tsalaguwetiyi (Cherokee), Waxhaw, Yesan (Tutelo), and Yį Įsuwą (Catawba) peoples. Most specimens used in this revision were obtained from collections made during the past 25 years by the authors, many collaborators, and prior students of the first author (see Acknowledgements). The types of all previously described taxa were loaned from the American Museum of Natural History (**AMNH**). Members of field expeditions searched appropriate microhabitats for spiders and collected specimens by hand or using an aspirator. Most spiders were preserved in the field in either 80% or 100% EtOH for subsequent molecular analysis. Molecular samples were later stored in a -80 °C freezer.

Geographic location data were taken in the field using a global positioning system (GPS) device, and later verified/adjusted using ACME Mapper (https://mapper.acme.com/). Map figures were generated by importing CVS files into the USGS Survey Map Viewer (https://maps.usgs.gov/map/) then adjusting terrain overlay and zoom levels.

We identified immature specimens using the following guidelines: 1) If immatures were collected in association with adults from the same geographic location and in the same microhabitats, these specimens were attributed to the same species, reflecting a very low probability of syntopy (three locations of > 450 unique collecting events; Suppl. material 1). We define syntopy as finding two or more species at the same geographic location (same collecting event), even though we cannot claim that spiders were found in identical microhabitats. 2) If only immatures were collected from a previously published location (generally caves), these specimens were attributed to the known species from this location. 3) Immature spiders from new locations without associated adults were not identified to species (see Suppl. material 2).

### ﻿Morphology-based species discovery

Some authors have divided species delimitation into a two-step process (Carstens et al. 2013), including a discovery phase (formulating species hypotheses) and a validation phase (formally testing these hypotheses using typically independent evidence). Of course, the validation phase can also be used to test previously formulated hypotheses, i.e., species described by prior authors. We used patterns of morphological variation to formulate putative new species hypotheses. Our general approach was to rely most on patterns of male genitalic variation for a priori species delimitation (as is almost universally applied in araneomorph spider taxonomy, see Bond et al. 2022), assuming that the complex structures of the male nesticid palp best reflect species divergence.

One caveat to our morphology-first approach is that we expect some morphological variation within species, and the distinction between geographic variation vs. species-level divergence is not obvious using only a qualitative approach (e.g., vs. conducting morphometrics and statistical analyses). We expected morphological variation within species because the habitats occupied by these spiders are naturally fragmented (e.g., populations found in caves, isolated mountain ranges, talus fields within mountain ranges, etc.), and the spiders themselves are dispersal-limited. For example, Hedin (1997a) used mitochondrial DNA sequence data to show that gene flow is highly constrained in both cave- and surface-dwelling taxa of the *tennesseensis* group. The combination of natural fragmentation plus dispersal-limitation provides ample opportunity for the evolution of morphological geographic variation. This expected pattern is revealed in the specimens examined here, where genitalic variation is sometimes observed within a single population (e.g., within the confines of a single cave), and variation across populations is common. Yaginuma (1977) discussed similar patterns of challenging intraspecific variation in Japanese nesticids.

This revision shows that female epigynal morphology is generally (but not always) more conserved within groups of closely related taxa. This impacted our revisionary research because adult males were not always available from all collecting locations. In these cases, our species assignments for female-only locations were less confident, and sometimes relied more heavily on geographic and/or genetic (a posteriori) evidence.

### ﻿UCE data collection and analysis

To formally test or validate morphological hypotheses using independent character evidence we gathered phylogenomic-scale UCE data and supplemented this nuclear perspective with mitochondrial data. Original UCE data were gathered for 95 specimens representing all but one previously described Appalachian *Nesticus* species and all putative new species (Suppl. material 3). Original data were combined with previously published UCE data for two specimens (see Suppl. material 3). We paid particular attention to genetic sampling from the type localities (or locations nearby) for previously described taxa, and more extensive geographic sampling was conducted for taxa with larger geographic distributions. UCE outgroup data were generated for *Nesticus* species from California and Mexico, and *Nesticellamogera* (Yaginuma, 1972) from Japan. In recent analyses of Ribera and Dimitrov (2023), Appalachian taxa were recovered as monophyletic and sister to taxa from Japan (*Cyclocarcinafloronoides* Kishida, 1942) and South Korea (*Nesticuskyongkeomsanensis* Namkung, 2002); these authors however did not include *Nesticus* taxa from Mexico in their analysis.

Genomic DNA was extracted from leg tissues using the DNeasy Kit (Qiagen). At least 200 ng was sent to RAPID genomics for UCE library prep (Suppl. material 3), where UCEs were captured using the spider-specific probe set (Kulkarni et al. 2020). Libraries were sequenced using HiSeq 4000 paired-end 150 bp reads. TRIMMOMATIC v. 0.39 (Bolger et al. 2014) was used to trim adapters and low quality base calls using the following commands: PE ILLUMINACLIP:$adaptersfasta:2:30:10:2:keepBothReads LEADING:5 TRAILING:15 SLIDINGWINDOW:4:15 MINLEN:40. SPADES v. 3.15.2 (Prjibelski et al. 2020) was then used for assembling clean reads, using the commands: spades.py --sc --careful –cov-cutoff auto. Assembled contigs were imported into the UCE pipeline PHYLUCE 1.6.7 (Faircloth 2016), where the merged arachnid and spider probesets (see Maddison et al. 2020) were matched to contigs using default (80, 80) match values. Sequence alignments were conducted in PHYLUCE using MAFFT (Katoh and Standley 2013) and trimmed using GBLOCKS (Castresana 2000). Matrices with at least 70% occupancy were imported into Geneious Prime 2021.1.1, where alignments were spot-checked.

Using individual UCE loci alignments (*n* = 801) as separate partitions, optimal models were selected using the merging strategy as described in Lanfear et al. (2012), implemented in IQ–TREE 2 (Nguyen et al. 2015). IQ–TREE 2 was also used to reconstruct concatenated maximum likelihood trees and calculate gene (gCF) and site (sCF) concordance factors. For every node of a reference tree, gCF is the percentage of “decisive” gene trees containing that node while sCF is the percentage of decisive sites (in an alignment) supporting a node (Lanfear 2018; Minh et al. 2018).

A species tree was also estimated under a multispecies coalescent model using ASTRAL v. 5.7.8 (Mirarab et al. 2014; Mirarab and Warnow 2015; Rabiee et al. 2019). Input gene trees were estimated using IQ–TREE 2 and treated as unrooted. Internal branch lengths were estimated in coalescent units, with branch support measured as both quartet scores (Sayyari and Mirarab 2016) and local posterior probability values (a function of number of loci and quartet frequencies; Sayyari and Mirarab 2016).

### ﻿Mitochondrial data collection and analysis

We generated mitochondrial sequences for 218 specimens using standard polymerase chain reaction (PCR) combined with Sanger sequencing. These Sanger data were collected prior to the UCE data (Suppl. material 3). Because there are very few instances of species sympatry as noted above, immature specimens were sometimes used for mitochondrial analysis, but in almost all cases immatures were associated with a sample of adult specimens from the same collecting event. PCR experiments targeted an approximately 1050 bp fragment of the mitochondrial cytochrome c oxidase subunit I (COI) gene region, using C1-J-1510/C1-N-2776 primers and amplification parameters as in Hedin and McCormack (2017). PCR amplification products were sequenced in both directions and sequence contigs were assembled and edited using Sequencher v. 4.2.2.

For specimens for which we had UCE data but no Sanger mitochondrial data, we used BLAST searches in Geneious to recover COI mitochondrial “by-catch” from UCE contigs. As proof of concept, we also included specimens for which we already had Sanger data to confirm the accuracy of the by-catch method and captured mitochondrial data (Suppl. material 3). In all cases by-catch and Sanger sequences from the same specimens were identical (see Results).

Combined Sanger and UCE by-catch mitochondrial data were manually aligned in Geneious. Phylogenetic analysis of the COI matrix was conducted using maximum likelihood searches implemented in IQ–TREE 2. The matrix was partitioned by codon position, with a best-fitting partition scheme (following Lanfear et al. 2012) found by possibly merging partitions (command: -s -p -m MFP+MERGE, with ultrafast bootstrap -B 1000).

### ﻿Integrative species delimitation

We defined species under an integrative species delimitation framework as follows: “single populations or sets of populations that share diagnostic male palpal morphologies and that are supported by nuclear phylogenomic monophyly”. This definition includes some necessary caveats. First, our nuclear sample is representative but obviously not exhaustive. We did not generate UCE data for all available sample locations and as such could not formally validate the placement of all specimens / populations. Second, as it was not possible to apply the monophyly criterion for putative species including only a single UCE sample (typically species known only from one location), we here considered long branch lengths, either in a concatenation or coalescent unit framework (long internal branch lengths for the latter). Third, given only qualitative assessments of variation, the concept of “diagnostic male palpal morphologies” is necessarily subjective, and we allowed for some intraspecific variation (for reasons argued above). The distinction between species level morphological variation vs. geographic variation within a species is not always obvious a priori, and we thus allowed the molecular results (nuclear data in particular) to help guide these decisions.

While mitochondrial evidence was sometimes useful in *Nesticus* species delimitation, we relied most heavily on nuclear gene tree patterns. Conspicuously, mitochondrial data sometimes failed to recover well-supported nuclear lineages, including some well-supported species, likely because of high mitochondrial divergences (see Results). Evidence for mitochondrial introgression and/or deep coalescence of mitochondrial lineages is also apparent within the Appalachian *Nesticus* fauna. Because of the incongruence sometimes observed between hypothesized species (supported by morphology and nuclear data) vs. clades recovered on mitochondrial gene trees, we put less emphasis on the mitochondrial evidence for validating morphological species limits. However, because of larger sample sizes (more geographic populations sampled) and generally higher rates of molecular evolution, the mitochondrial data did provide useful phylogeographic information, and were sometimes used to place some populations for which we only collected female specimens.

Geography also played a secondary role in species delimitation because almost all *Nesticus* species are found in allopatry and typically occupy spatially contiguous geographical distributions that reflect landscape features (e.g., isolated mountain ranges).

### ﻿Taxonomy

**Figure 2.**
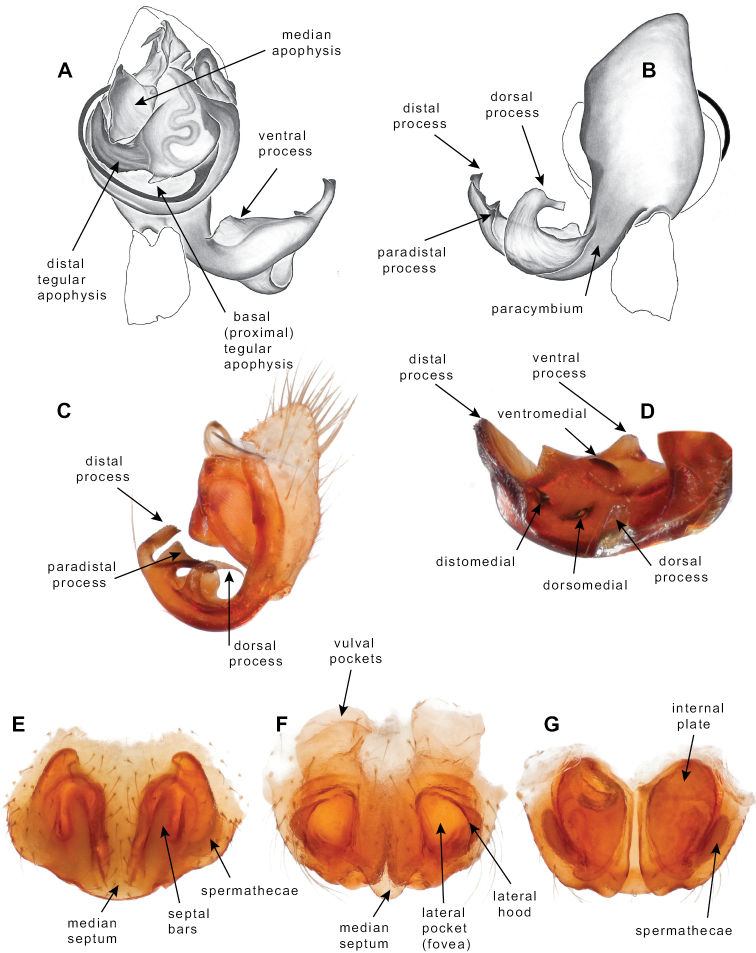
Graphical overview of terminology used to describe genitalic morphology. Tennessee, Overton Co., Obe Lee Cave, *Nesticusstygius*, ♂ MCH specimen #1882 palp ventral (**A**), dorsal (**B**) **C** North Carolina, Haywood Co., near Steestachee Bald overlook, *N.silvanus*, MCH specimen #1145, ♂ palp dorsal **D** North Carolina, Rutherford Co., Moonshiner’s Cave, *N.brimleyi*, MCH 99_014, ♂ paracymbium medial **E** North Carolina, Clay Co., Big Tuni Creek, MCH 02_171, epigynum ventral **F** North Carolina, Henderson Co., W of Bat Cave, MCH 07_134, epigynum ventral **G** North Carolina, Buncombe Co., SW of Cane River Gap, MCH 01_167, epigynum dorsal.

Standard terminology used to describe male and female genitalic morphology follows Coyle and McGarity (1992) and Hedin and Dellinger (2005), as illustrated in Fig. 2A–G. For the medial processes of the paracymbium (Fig. 2D), unless all three are present simultaneously, exact positional homology over distant taxa is uncertain because the relative placement of these processes does appear to evolve (move) across taxa. For example, the definition of dorsomedial vs. distomedial depends upon the relative placement of these two when both are present; if one is lacking then our inference of deeper homology (across species groups) should be viewed as necessarily uncertain. The same argument applies to paradistal vs. dorsal paracymbial processes in some cases. If both processes are not present in a species (or species group), we have observed that the relative positioning of these processes can vary among taxa, challenging our inference of deeper (among species group) positional homology. We emphasize that these issues of positional homology do not impact our species diagnoses, as most diagnoses are restricted to comparisons among relatively closely related taxa in the same species group (as defined below).

With regards to epigynal morphology, the internal fertilization and copulatory ducts are difficult to visualize in *Nesticus* without examination under a compound microscope. Because our drawings and digital images generally do not reveal these details our verbal descriptions similarly emphasize more readily visualized aspects of epigynal morphology.

Because adult body size, leg lengths, and carapace / abdominal color patterns were found to be variable both within and among populations of the same species (see examples below), we generally do not comment upon this variation in the species descriptions below. Instead, intraspecific variation in male and female genitalia (if present) is emphasized.

Holotype and paratype specimens have been deposited at the Bohart Museum of Entomology (**BME**) at UC Davis. All other specimens referenced with San Diego State University (**SDSU**) or M Hedin (**MCH**) numbers are currently housed in the San Diego State University Terrestrial Arthropod Collection (**SDSU_TAC**).

The following character abbreviations are used in our species descriptions: **BL** = body length (CL plus length of abdomen measured in dorsal view); **CL** = carapace length (from posterior edge to front edge of clypeus, measured at midline); **CW** = maximum carapace width. Lengths of leg I segments are measured in retrolateral view as a straight-line distance from opposite articulation points on the dorsal surface of each segment, reported in Species Descriptions as: Total (fm, pt, ti, mt, ta). All appendage measurements were recorded from the left appendage, unless noted otherwise, and are reported in mm. Measurements at SDSU were taken with an Olympus SZX12 stereomicroscope with 10× ocular lenses fitted with an eyepiece micrometer. All measurements were performed at 2× magnification. Measurements at the University of Indianapolis were taken using a Leica M165C stereomicroscope with an attached DMC2900 camera and calibrated annotation tools within the Leica Application Suite X software (Leica Microsystems, LAS Suite X, v. 3.0.12.21488).

Ink drawings of male and female genitalia were made by Nadine Dupérré. A digital camera attached to a stereomicroscope was used to capture images, which were then enlarged and printed. A tracing of this printed image was detailed and shadowed with repeated reference to the specimen under a microscope. Epigyna were removed and cleared with lactic acid prior to illustration. The left palp of male spiders was illustrated in all cases.

Specimens were digitally imaged at SDSU using a Visionary Digital BK plus system including a Canon 40D digital camera and Infinity Optics Long Distance Microscope. Individual images were combined into a composite image using Zerene Stacker v. 1.04 software; this composite image was then edited using Adobe Photoshop. Epigyna were dissected from specimens using fine forceps, immersed for 2–5 min in BioQuip specimen clearing fluid on a depression slide, then imaged directly in this fluid on the slides. Other images were taken with specimens immersed in filtered 70% EtOH, using KY jelly to secure samples.

## ﻿Results and discussion

### ﻿Specimen and geographic sampling

The total morphological sample considered is summarized in Suppl. material 1. This included more than 2100 adult specimens from ~ 480 unique collecting events. We also examined type specimens for all previously described taxa housed at the AMNH.

### ﻿Morphology-based species discovery

Based on the examination of male and female morphology we hypothesized the following new species *a priori* (discovery phase): two undescribed species in the *tennesseensis* group, two undescribed species in the *nasicus* group, two undescribed species in the *barrowsi* group, and three undescribed species in the *reclusus* group (see group definitions below). We also questioned the species-level status of taxa for two species pairs in the *reclusus* group (*Nesticusstupkai* vs. *N.bishopi*; *N.reclusus* vs. *N.cooperi*). We also noted novel patterns of morphological variation within several described species, which could technically represent new species, but *a priori* treated this as intraspecific variation.

For species delimitation of *Nesticusjemisinae* sp. nov. we did not follow the morphology first framework. Instead, a female of uncertain affinity was first included in a UCE experiment and a long phylogenetic branch was discovered. We subsequently requested that colleagues collect additional specimens from the type locality, and upon inspection, both males and females proved to be morphologically unique.

### ﻿UCE data and results

We gathered original UCE data for 95 specimens, supplemented with previously published data for two specimens (Suppl. material 3). Specimens from the type locality (or near type locality) were sampled for 27 of 28 previously described species (Suppl. material 3). Raw read data have been submitted to the Sequence Read Archive (BioProject ID PRJNA912717). After processing in PHYLUCE and GENEIOUS data were available for 801 nuclear loci with a total concatenated length of 863,026 base pairs. Input alignments, analysis log files, and output tree files have been included as Suppl. material 4.

**Figure 3.**
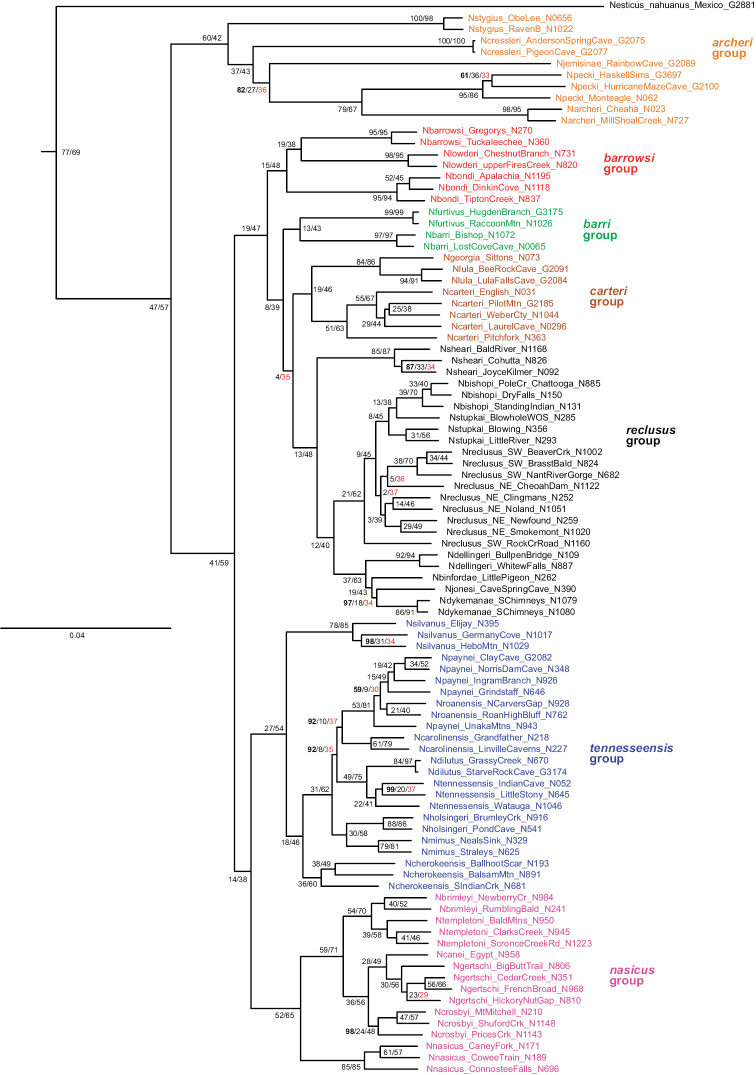
UCE concatenated maximum likelihood tree. Distant outgroups removed (for graphical purposes), specimen numbers correspond to those in Suppl. material 3 (with detailed location provided in Suppl. material 1). Node numbers correspond to bootstrap (bold text) / gCF / sCF. Only bootstrap values below 100 shown, all others 100. gCF and sCF values rounded to nearest integer; sCF values below 38 highlighted with red text.

**Figure 4.**
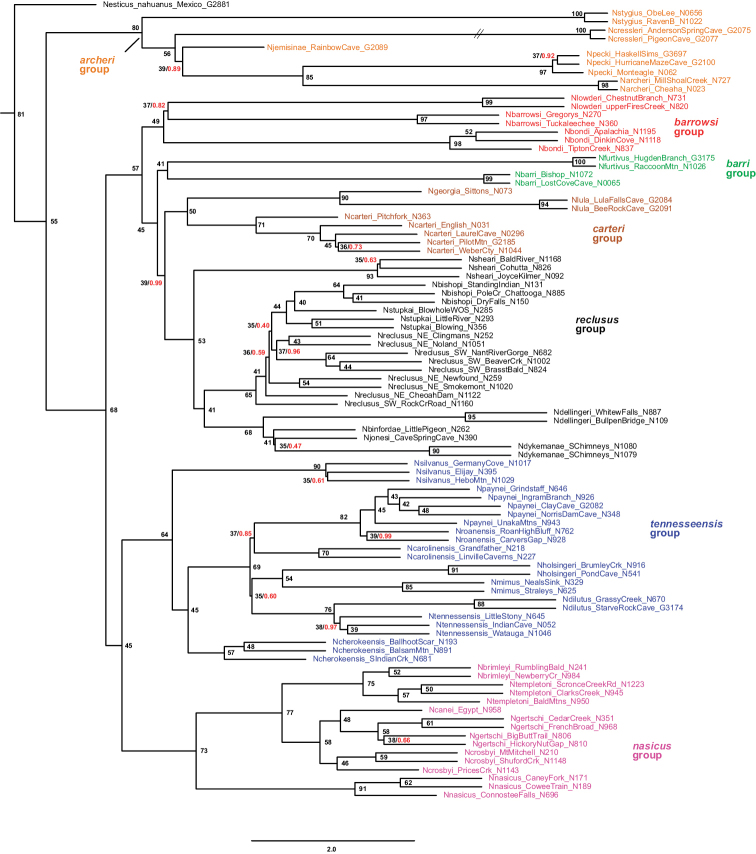
UCE ASTRAL species tree. Distant outgroups removed (for graphical purposes), specimen numbers correspond to those in Suppl. material 3 (with detailed location provided in Suppl. material 1). Included are quartet support values (rounded to nearest integer), and local posterior probability values (if less than 1.0, red text). Branch lengths in coalescent units for internal branches only, terminal branch lengths arbitrary. Internal branch to *Nesticuscressleri* truncated for graphical purposes.

Concatenated maximum likelihood and coalescent-based ASTRAL analyses, with very different analytical assumptions, largely agree on overall tree structure (Figs 3, 4). Rooting with *Nesticellamogera* recovers an Appalachian clade sister to a *Nesticus* species from Mexico, both sister to *Nesticussilvestrii* Fage, 1929 from California. We here define seven primary regional species groups within the Appalachian radiation (Figs 3, 4), all strongly supported on both concatenated and ASTRAL trees (Figs 3, 4) and typically diagnosed by morphological synapomorphies. These morphological synapomorphies are discussed in the Taxonomy section below. The seven primary species groups are as follows: *archeri* group, sister to all other Appalachian lineages; the latter clade including the *tennesseensis* group (first recognized by Gertsch 1984) sister to the *nasicus* group (hypothesized by Coyle and McGarity 1992); remaining taxa in a clade including the *barrowsi*, *barri*, *carteri*, and *reclusus* groups.

### ﻿Mitochondrial data and results

COI sequences were generated for 241 total specimens (Suppl. material 3). For five specimens we gathered duplicate Sanger and UCE by-catch data (always 100% identical), and data for 18 specimens were derived from UCE by-catch only (Suppl. material 3). All mitochondrial sequences have been deposited to GenBank (OQ094967–OQ095207). Input alignments, analysis log files, and output tree files have been included as Suppl. material 4.

Mitochondrial outgroup relationships are as recovered in the UCE data, and the Appalachian fauna is recovered as monophyletic (Fig. 6). The *archeri* group is recovered and identical to nuclear data in internal species relationships resolution. The *tennesseensis* group is also recovered. Beyond this, mitochondrial data failed to recover the remaining groups. Several otherwise well-supported species are also not recovered on the mitochondrial tree (e.g., *N.carteri*). Multiple mitochondrial sequences are available for most species, and all exhibit high intraspecific mitochondrial divergences, where essentially all sample locations are genetically unique. Additional mitochondrial tree details are discussed in the Taxonomy section below for each species or species group.

### ﻿Integrative species delimitation

**Figure 5.**
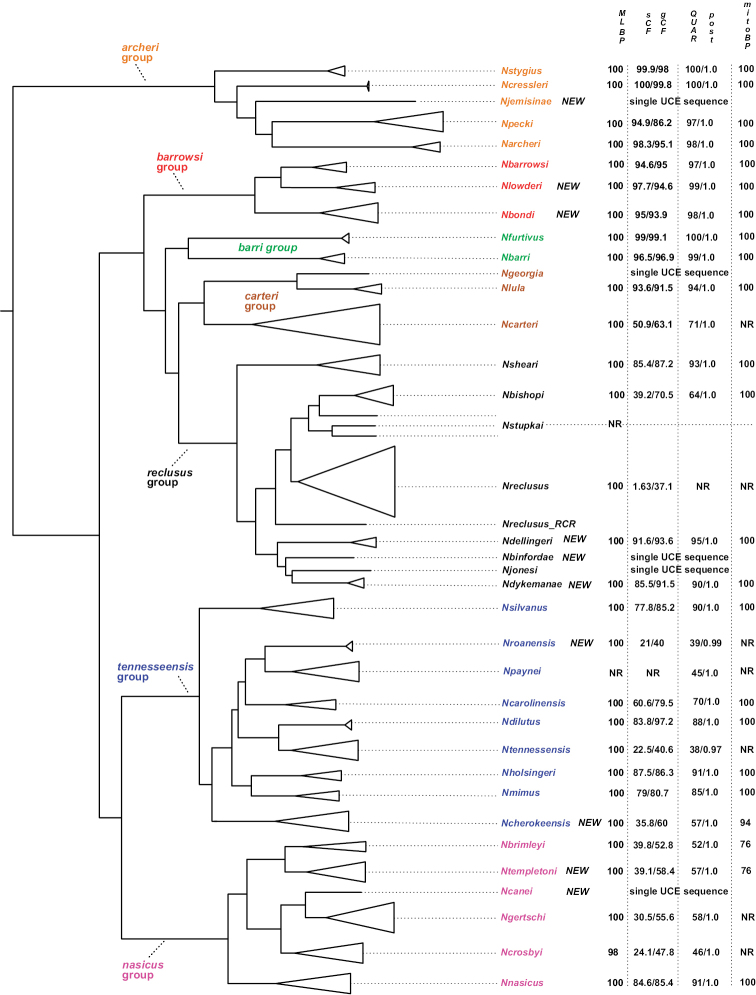
Summary species tree (from UCE topologies), with summary statistics for species support. Branch lengths are arbitrary. New species highlighted. NR = not recovered as clade.

For the nine new species discovered by examination of patterns of male palpal morphology (see above), six are strongly supported (validated) by nuclear gene tree monophyly and associated support metrics (ML bootstrap, concordance factors, ASTRAL quartet scores, and ASTRAL local posterior probabilities; see Fig. 5). One of the nine newly discovered species, *N.roanensis* sp. nov., was weakly supported by phylogenomic evidence and not supported by mitochondrial evidence but was retained as valid (see taxonomic section for this species). Two of the nine species hypotheses could not be formally validated using the monophyly criterion as only a single UCE sample was included.

**Figure 6.**
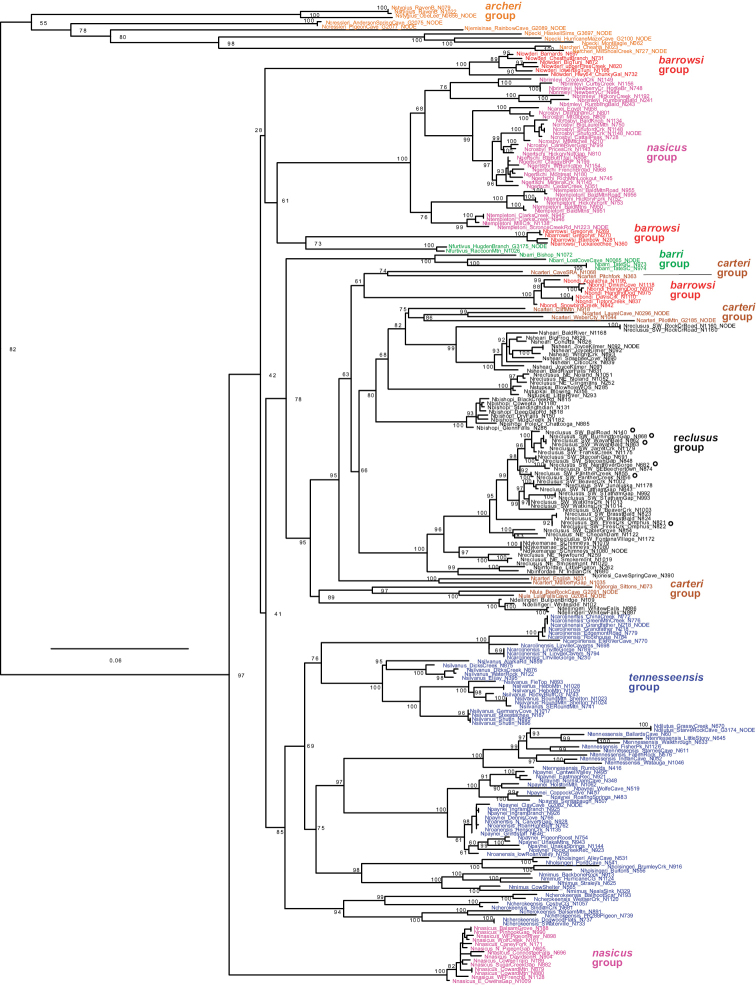
COI IQT gene tree. Distant outgroups trimmed. Specimen numbers correspond to those in Suppl. material 3 (with detailed location provided in Suppl. material 1). Sequences from UCE capture denoted with “NODE”. Populations with a “*Nesticuscooperi*-like” male paracymbium denoted by small circles. Not all tip bootstrap values shown (see Suppl. material 4 for .tre file with all bootstrap values).

The nuclear phylogenomic data also mostly strongly supported previously described species. We note that none of these prior hypotheses have ever been tested (validated) using independent data, as is true for almost all described spider species (see Bond et al. 2022). The previously described species pairs (*Nesticusreclusus* vs. *N.cooperi*; *N.stupkai* vs. *N.bishopi*) proved to represent difficult species delimitation scenarios. These challenging cases revealed phylogenetically discordant patterns (e.g., morphological groups conflicting with gene tree clades, mitonuclear discordance, genetic divergence without coincident morphological change) and are discussed more fully in the relevant Taxonomy sections below.

Patterns of genitalic variation across populations within what we considered as single species are summarized in the Taxonomy section for each species. These patterns of variation are based on much larger sample sizes than previously considered, so most are newly reported here.

### ﻿Niche conservatism and conservation

Below we briefly describe patterns of niche conservatism in Appalachian *Nesticus*, and how this might have impacted the evolution of sympatry, geographic distributions, and species endemicity. Future manuscripts will more comprehensively address these issues.

Contrasting with patterns of intraspecific genetic divergence, the Appalachian *Nesticus* fauna is rather conserved in somatic morphology and ecology. This is certainly true for montane species, all of which occur in similar microhabitats and are very similar in their general habitus. Most ecological and somatic morphological evolution is associated with the evolution of cave-dwelling (troglomorphy), involving characters such as body size, leg length, eye reduction, and pigment reduction. We hypothesize that this general niche conservatism has strongly impacted the evolution of sympatry and syntopy. Either because of ecological similarity or reproductive interference (e.g., Ribeiro and Spielman 1986; Goldberg and Lande 2007), syntopy is extremely rare in the Appalachian radiation. Of 480 unique collecting events, we found members of two different species in syntopy on only three occasions, each time involving relatively distant phylogenetic relatives. These cases are more fully discussed in the Taxonomy section below.

We hypothesize that niche conservatism, and perhaps interactions with competing *Nesticus* species over evolutionary time, has impacted the evolution of endemism in the group. Many species have very small geographic distributions, including three species (*Nesticuscanei* sp. nov., *N.jemisinae* sp. nov., *N.jonesi*) known only from single locations, and many others are known only from a handful of geographically adjacent locations. Many of these microendemic species also appear to be naturally rare (at low abundance). These taxa deserve conservation attention and continued conservation monitoring to ensure their long-term persistence.

The Appalachian *Nesticus* radiation has many parallels with the spider genus *Troglohyphantes* Joseph, 1882 (Linyphiidae) from southern Europe, including many taxa in a small area, many microendemic species, habitat and physiological specializations, rare sympatry, and conservation relevance (Isaia et al. 2017). Researchers have begun to understand how climate change will impact the evolution of both cave and montane-restricted species in *Troglohyphantes* (Mammola et al. 2018, 2019), and have developed conservation profiles for several at-risk species (Milano et al. 2022), developing this taxon into a model genus for invertebrate conservation. With the phylogenomic, geographic, and morphological taxonomic framework provided in this revision we argue that *Nesticus* might similarly be developed into a model taxon to understand the impacts of climate (and other abiotic and biotic) change on the specialized cave- and montane-restricted animal fauna of the Appalachian region.

## ﻿Taxonomy

**Figure 7.**
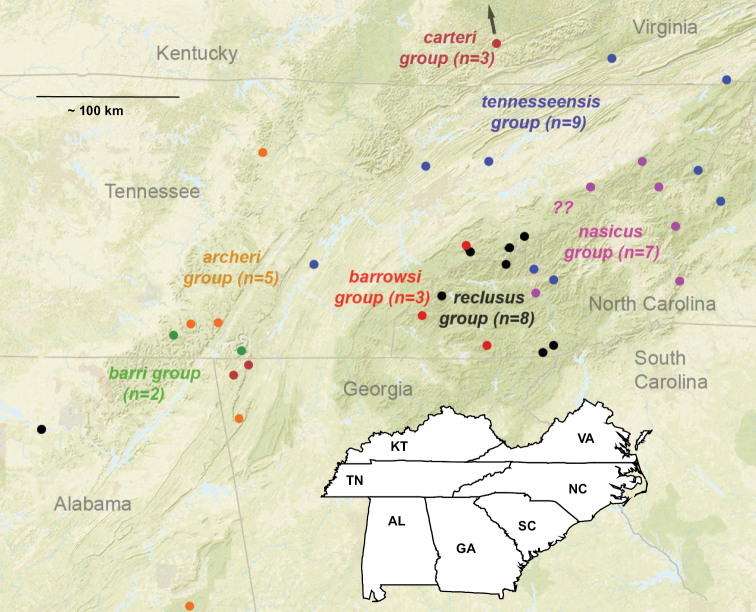
Map of all type localities. Type locality for *Nesticussecretus* is geographically uncertain (see text), designated with a question mark. Inset map shows full map for US states of interest. Scale bar: approximately 100 kilometers.

The taxonomy presented below is structured to follow phylogenomic results, including species groups (treating the *archeri* group first) and order of presentation of species within groups (treating early diverging species first). We do not provide a key to Appalachian species, but rather rely upon the combination of diagnostic morphological features and mostly allopatric geographic distributions to identify specimens. Fig. 7 illustrates the geographic distribution of type localities for all known 37 Appalachian species. Qualitative assessments of conservation status, based on data amassed during the past 25 years, are also emphasized below.

### ﻿Family Nesticidae Simon, 1894

**Genus *Nesticus* Thorell, 1869**

#### ﻿*archeri* group, including:

*Nesticusstygius* Gertsch, 1984

*Nesticuscressleri* Zigler & Milne, 2022

*Nesticusjemisinae* sp. nov.

*Nesticusarcheri* Gertsch, 1984

*Nesticuspecki* Hedin & Dellinger, 2005

Both UCE and mitochondrial data recover this clade and identical species relationships within this clade (Figs 3, 4, 6). Two eyeless taxa (*Nesticusstygius*, *N.cressleri*) form a paraphyletic grade with respect to three eyed taxa. Species in the group are each morphologically distinctive, subtended by long internal phylogenetic branches with high support values for all metrics and minimal apparent gene tree conflict as measured by concordance factor values (Figs 3–6). Overall, species delimitation within this group is straightforward, likely reflecting a relatively more ancient history (and extinction of intervening lineages) within the group.

Males within this species group possess palps with a forked tegular apophysis, including a larger, darkened distal tegular apophysis that extends underneath the median apophysis and a pointed basal tegular apophysis (Figs 2A, 9A, 11A). Females possess distinctive skinny or banana-shaped spermathecae that extend anteriorly near the outer edges of the epigynum (Figs 10A–C, 11C).

**Figure 8.**
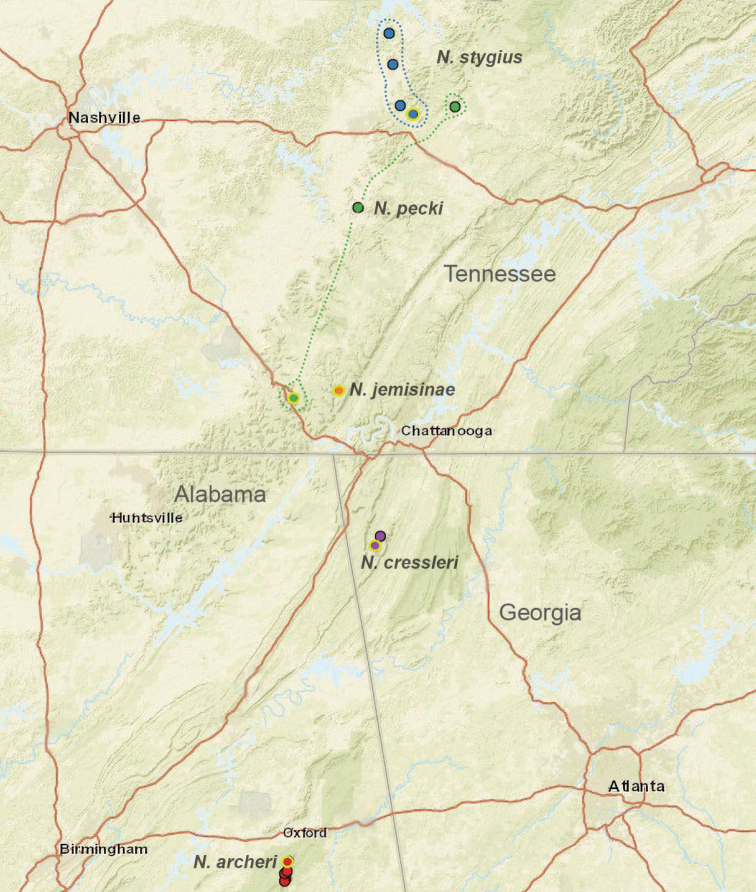
Distribution of the *archeri* group, including *Nesticusarcheri*, *N.pecki*, *N.jemisinae*, *N.cressleri*, and *N.stygius.* State boundaries and major cities shown for geographic context. Dashed lines circumscribe the distributions of *N.pecki* and *N.stygius*.

Species in this species group are distributed in caves on the Cumberland Plateau, and southwards to suitable surface microhabitats at Talladega Mountain in east-central Alabama (Fig. 8). Almost all species appear to have relictual distributions, most species are microendemic, including *Nesticusjemisinae* sp. nov. known only from a single geographic location (Fig. 8). Because of both rarity and microendemism, additional undiscovered populations and species representing this species group are likely. For example, collecting efforts in disjunct “sky island” ridges to the north of Talladega Mountain in east-central Alabama (e.g., Choccolocco Mountain, Weisner Mountain, Bogan Mountain) will likely uncover undescribed *archeri* group species.

**Figure 9.**
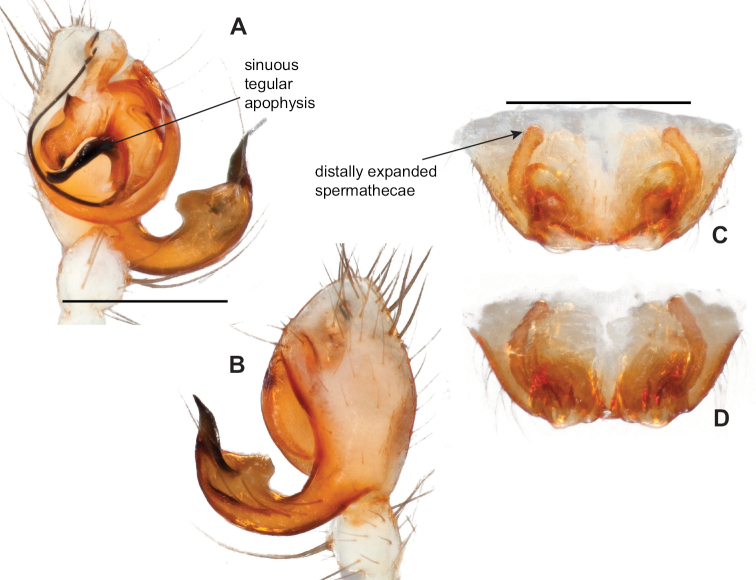
*Nesticusjemisinae* sp. nov. Tennessee, Marion Co., Rainbow Cave, ♂ holotype, SDSU_TAC000662, ventral (**A**), dorsal (**B**). Rainbow Cave, ♀ paratype, SDSU_TAC000663, ventral (**C**), dorsal (**D**). Scale bar: 0.5 mm.

**Figure 10.**
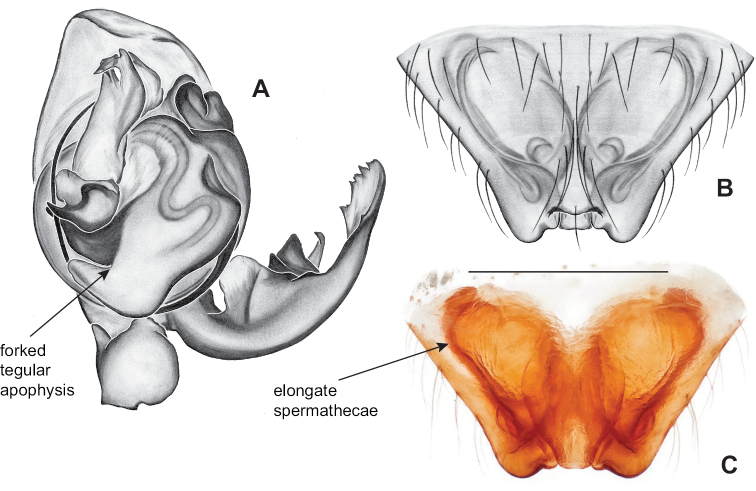
*Nesticusarcheri***A** Alabama, Clay Co., vicinity Mill Shoal Creek, ♂ specimen MCH #2132, ventral palp **B** Alabama, Clay Co., vicinity Mill Shoal Creek, ♀ specimen MCH #2129, epigynum, ventral view **C** dorsal view. Scale bar: 0.5 mm.

**Figure 11.**
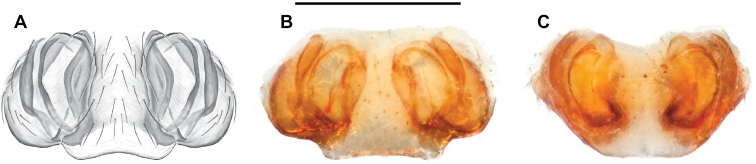
*Nesticuspecki* epigynum variation, ventral views **A** Tennessee, Marion Co., Monteagle Saltpeter Cave, specimen #1625 **B** Tennessee, Fentress Co., Hurricane Maze Cave, MLN 13–063 (SDSU_G2100) **C** Tennessee, White Co., Haskell Sims Cave, MLN 14–004 (SDSU_G3697). Scale bar: 0.5 mm.

##### ﻿ Nesticus stygius

Taxon classificationAnimaliaAraneaeNesticidae

Gertsch, 1984

424DB121-3E9A-5F6B-8221-88C5D82DDA24

Fig. 2A, B

Nesticus stygius Gertsch, 1984: 36, figs 170–172; Hedin and Dellinger 2005: 8, figs 11, 12.

###### Material examined.

**New collections from type locality**: USA – **Tennessee, Overton Co.** • ♂, 3♀; Obe Lee Cave, N of Monterey; 11 Oct. 1993; M. Hedin, C. Phillips leg.; **Non type material**: – **Overton Co.** • ♂, 1 imm; East Water Supply Cave, TOV15; 13 Jul. 2013; M.L. Niemiller, K.D. Kendall leg.; MLN 13–036; • 7♀; Raven Bluff Cave, NW of Allons; 26 Sep. 1992; M. Hedin, S. O’Kane leg.; • 2♂, ♀; Raven Bluff Cave; 1 Oct. 1991; M. Hedin, K. Crandall, A. Gerber leg.; • ♀; Raven Bluff Cave; 28 Aug. 2005; M. Hedin, R. Keith, J. Starrett, S. Thomas leg.; MCH 05_101; • 4♀, 1 imm; Raven Bluff Cave, TOV28; 3 Sep. 2017; M.L. Niemiller, N. Mann leg.; MLN 17–008.6; • ♀, 1 imm; Webb Cave, TOV39; 1 Oct. 2017; N.S. Gladstone, E.T. Carter, L. Hayter leg.; NSG 17–TOV39.17.

###### Diagnosis.

Morphological diagnosis as in Hedin and Dellinger (2005).

###### Distribution.

This highly troglomorphic taxon is only known from a few caves on the western margin of the Cumberland Plateau in Overton County, north-central Tennessee (Fig. 8; Hedin and Dellinger 2005: fig. 1).

##### ﻿ Nesticus cressleri

Taxon classificationAnimaliaAraneaeNesticidae

Zigler & Milne, 2022

4F805820-79AD-55F5-9F9B-9C48736C8FF1

Nesticus cressleri Zigler & Milne, 2022: 293, figs 1B, D, 4, 5, 7.

###### Material examined.

**Type material**: USA – **Georgia, Walker Co.** • ♂, ♀, 1 imm; Anderson Spring Cave (GWK46); 11 Jun. 2014; K.S. Zigler, L. Carver, W.T. Coleman leg.; KSZ 13–159; **Non type material**: – **Walker Co.** • ♀, 1 imm; Pigeon Cave (GWK57); 3 Aug. 2013; L. Carver, A. Cressler, K.S. Zigler leg.; KSZ 13–184.

###### Diagnosis.

Morphological diagnosis as in Zigler and Milne (2022).

###### Distribution and natural history.

This troglomorphic taxon is known only from three geographically adjacent caves on Pigeon Mountain in Walker County, Georgia (Fig. 8; Zigler and Milne 2022: fig. 7).

##### ﻿ Nesticus jemisinae sp. nov.

Taxon classificationAnimaliaAraneaeNesticidae

209BAACA-F374-51E4-871F-0AC8359AAFFA

https://zoobank.org/EC278B87-636E-41F5-AA3E-59599C83AB18

Fig. 9A–D

###### Material examined.

**Type material: *Holotype***: USA – **Tennessee, Marion Co.** • ♂ holotype; Rainbow Cave (TMN20); 20 Oct. 2021; K.S. Zigler leg.; SDSU_TAC000662; ***Paratypes***: • ♀ paratype; data as for holotype; SDSU_TAC000663; • ♂, 2♀ paratypes; data as for holotype; SDSU_TAC000664; **Non type material**: – **Marion Co.** • ♀; Rainbow Cave (TMN20); 10 Nov. 2013; K.S. Zigler leg.; KSZ 14–248. • 5 imm; Rainbow Cave (TMN20); 20 Oct. 2021; K.S. Zigler leg.

###### Diagnosis.

Easily distinguished from other members of the *archeri* group. Distinctly small-bodied *Nesticusjemisinae* possesses well-developed eyes, different from the eyeless *N.cressleri* and *N.stygius*. Males possess a relatively simple paracymbium, contrasting with the multiple apophyses of the complex paracymbium of *N.archeri* (Fig. 10A) and *N.pecki* (Hedin and Dellinger 2005: figs 17, 18). The distinctive tegular apophysis is dark and sinuous, extending under the median apophysis. Female *N.jemisinae* may also be distinguished from the latter species by the epigynum. The epigynum of *N.archeri* is subtriangular with large anterior fovea and a narrow median septum (Fig. 10B, C), *N.pecki* possesses a broad posteriorly-broadening median septum (Fig. 11A–C), and *N.jemisinae* possesses a posteriorly-pointed median septum with the spermathecae expanded into small bulbs distally (Fig. 9C, D).

###### Description of ♂ holotype

**(SDSU_TAC000662; Fig. 9A, B).** Carapace and appendages are a dusky yellow. Abdomen mottled gray with darker patches between lighter parts. Eyes ringed with black and equally well-developed except for AME, which are significantly reduced. Carapace 1.18 long, 1.09 wide. Total body length 2.43. Leg I total length 9.50 (2.74, 0.46, 2.73, 2.56, 1.01), leg I / CW ratio 8.72, leg formula 1423. Paracymbium of palp relatively simple with a large proximally directed ventral process and a dark sinuous distal process. Dorsal process of paracymbium largely reduced to a shallow pocket on the distal edge. Palp tegular apophysis dark, long, narrow, pointed, and extends under median apophysis. Median apophysis elongated towards base of palp and angled proximally at tip.

###### ♂ Variation.

No significant genitalic variation was noted in the material examined.

###### Description of ♀ paratype

**(SDSU_TAC000663; Fig. 9C, D).** Color of carapace, appendages, and abdomen as in male. Eyes as in male. CL 1.20, CW 0.97. Total body length 2.61. Leg I total length 7.96 (2.30, 0.44, 2.29, 1.98, 0.95), Ieg I / CW ratio 8.21, leg formula 1423. Epigynum width approximately half the width of the abdomen. Median septum pointed posteriorly and flanked by fovea along posterior margin. Internal foveal pockets visible from ventral inspection without dissection extending anteriorly angled outwards. Thin, elongate spermathecae curve slightly on outside margins of epigynum, extending anteriorly beyond foveal pockets, expanded into small bulbs distally.

###### ♀ Variation.

No significant genitalic variation was noted in the material examined.

###### Distribution and natural history.

Known only from Rainbow Cave, located near Pocket Creek, a tributary to the Little Sequatchie River (Fig. 8). This cave is approximately 200 meters in length; spiders were collected ~ 50 meters from the cave entrance on the cave ceiling and walls, in total darkness.

###### Etymology.

The specific name is a matronym in honor of N. K. Jemisin whose ‘Broken Earth’ book series features a subterranean colony, including scientists who study caves.

###### Remarks.

*Nesticusjemisinae* sp. nov. is a relictual, single-site endemic whose morphology is quite distinct from that of other members of the species group. This species is nested within a diverged nuclear and mitochondrial subclade of the *archeri* group, sister to *N.pecki* and *N.archeri* (Figs 3, 4, 6).

##### ﻿ Nesticus archeri

Taxon classificationAnimaliaAraneaeNesticidae

Gertsch, 1984

F073E5C5-7498-5C9F-8488-CB1EEE2DEBD7

Fig. 10A–C

Nesticus archeri Gertsch, 1984: 32, figs 115–117, 129–131.

###### Material examined.

**Type material: *Holotype***: USA – **Alabama** • 1♂; Mt. Cheaha, Cheaha State Park; 21 Apr. 1947; A.F. Archer leg; AMNH. **Non type material**: USA – Alabama, **Clay Co.** • 2♂, 3♀; Cheaha State Park, 0.5 mi N Hernandez Peak, along Pinhoti Trail; 33.4645°N, -85.8113°W; 26 Mar. 1995; M. Hedin, B. Dellinger leg.; • 2♂, 2♀; Cheaha State Park, N side of McDill Point; 33.4547°N, -85.8205°W; 26 Mar. 1995; M. Hedin, B. Dellinger leg.; • 2♂, 4♀; Cheaha State Park, Pinhoti Trail, 0.5 mi. S Hernandez Peak; 33.4537°N, -85.8144°W; 26 Mar. 1995; M. Hedin, B. Dellinger leg.; • ♂, 3♀; Talladega Mountain, Cheaha Wilderness, Nubbin Creek Trail, vicinity Mill Shoal Creek; 33.4269°N, -85.8177°W; 26 Mar. 1995; M. Hedin, B. Dellinger leg.; • ♂, 3♀; Talladega Mountain, Talladega National Forest, near headwaters of Cave Creek; 33.4344°N, -85.8128°W; 26 Mar. 1995; M. Hedin, B. Dellinger leg.; – **Cleburne Co.** • 1 imm; Cheaha State Park, just north of Bald Rock; 33.4966°N, -85.8075°W; 27 Sep. 1991; M. Hedin, K. Crandall leg.; • 4♂, 13♀; Cheaha State Park, just north of Bald Rock; 33.4966°N, -85.8075°W; 24 Sep. 1992; M. Hedin, S. O’Kane leg.; • 2♂, 2♀; Cheaha State Park, vicinity Bald Rock saddle, 0.5 mi NE Bald Rock; 33.4985°N, -85.8028°W; 25 Mar. 1995; M. Hedin, B. Dellinger leg.

###### Diagnosis.

The only *Nesticus* species with a surface-dwelling habitus (small-bodied, darkly pigmented, well-developed eyes) in the region, and the only known *Nesticus* from Talladega Mountain. Male palp with a forked tegular apophysis, distal (highly sclerotized) fork lying behind pointed basal part of median apophysis in ventral view (Fig. 10A.). Paracymbium also distinctive, with prominent, sclerotized paradistal process. Very distinctive truncate, heart-shaped epigynum with a narrow median septum, large lateral pockets, and elongate spermathecae that hug the outer edges of the epigynal pockets, curving inwards anteriorly (Fig. 10B, C).

###### Variation.

No significant male or female genitalic variation was noted in the material examined.

###### Distribution and natural history.

All known records are from high elevation habitats (most above 600 m, but as low as 430 m) on Talladega Mountain, east-central Alabama (Fig. 8). This species was previously known only from type material collected at an unspecified location within Cheaha State Park, at the northern end of Talladega Mountain. New collections suggest that *Nesticusarcheri* can be found at several places on Talladega Mountain, spanning an approximately 10^2^ kilometer area from near Bald Rock (northern side of Cheaha Mountain) in the north to Mill Shoal Creek in the south.

This species has been collected in dark, cool, relatively moist near-surface habitats. For example, field notes from 1992 collections north of Bald Rock indicate that spiders were collected from “below bluffs on a steep hillside”, in “talus with a heavy leaf litter cover”, where spiders were “most abundant under large rocks close to the surface”. This situation compares favorably with the original “heavy talus of ravine” collections made by A.F. Archer, and the 1995 Hedin and Dellinger collections, most of which were made in north-facing talus, although at least one collection was from southwest-facing talus. This species is perhaps not as uncommon as previously believed and (in the late 1990s) was found consistently in suitable microhabitats.

###### Remarks.

We view Gertsch’s drawing of the male conductor (fig. 129) as inaccurate (compare to Fig. 10A).

##### ﻿ Nesticus pecki

Taxon classificationAnimaliaAraneaeNesticidae

Hedin & Dellinger, 2005

F45583DC-E6FA-5542-9F10-CE124A7D1817

Fig. 11A–C

Nesticus pecki Hedin & Dellinger, 2005: 14, figs 17–20.

###### Material examined.

**Type material: *Holotype***: USA – **Tennessee, Marion Co.** • ♂ holotype; Monteagle Saltpeter Cave, ~ 6.4 km SE of Monteagle; 26 Sep. 1992; M. Hedin, J. Hedin, S. O’Kane leg.; MCH1624; • ♀ paratype; data as for holotype; MCH1625; • 2♀; Monteagle Saltpeter Cave; 29 Sep. 1991; M. Hedin, K. Crandall, A. Gerber leg.; MCH1012, MCH1013; **Non type material**: – **Fentress Co.** • ♀; Hurricane Maze Cave (TFE331); 31 Aug. 2013; M.L. Niemiller, G. Moni, K. Bobo, A. Crabtree, B. Reeves, K. Pasternak leg.; MLN 13–063; – **White Co.** • 8♀; Haskell Sims Cave; 18 Jan. 2014; M.L. Niemiller, E.T. Carter, G. Moni, C. Sutherland leg.; MLN 14–004.

###### Diagnosis.

Morphological diagnosis as summarized in Hedin and Dellinger (2005), spiders small-bodied with well-developed eyes, males with a thickened and chisel-like tegular apophysis, females with a posteriorly-broadened median septum (Fig. 11A–C).

###### Variation.

The Hurricane Maze Cave specimen is similar to specimens from the type locality, possessing a short and wide epigynum with a posteriorly flaring median septum and banana-shaped spermathecae with narrow bases, lying just lateral to fovea but inside of the sclerotized epigynal outline (Fig. 11A, B). Haskell Sims Cave specimens are fairly different, the posterior edge of the median septum rounded instead of flared, and with internal plates close to touching (Fig. 11C). Given this relatively divergent female morphology it would be useful to attempt to collect adult males from this northern disjunct cave location (Fig. 8).

###### Distribution and natural history.

Previously known only from the type locality in southeastern Tennessee (Hedin and Dellinger 2005, fig. 1). We report here new important northern records from Haskell Sims and Hurricane Maze Caves, extending the geographic distribution of this species significantly northwards (Fig. 8, Suppl. material 1). More collecting on the Cumberland Plateau will likely result in additional new distributional records, although the species appears to be naturally rare. Hedin and Dellinger (2005) and Carver et al. (2016) reported on the rarity of this species at the type locality.

#### ﻿*tennesseensis* group, including:

*Nesticussilvanus* Gertsch, 1984

*Nesticuscherokeensis* sp. nov.

*Nesticusholsingeri* Gertsch, 1984

*Nesticusmimus* Gertsch, 1984

*Nesticustennesseensis* (Petrunkevitch, 1925)

*Nesticusdilutus* Gertsch, 1984

*Nesticuscarolinensis* (Bishop, 1950)

*Nesticuspaynei* Gertsch, 1984

*Nesticusroanensis* sp. nov.

This species group is recovered as monophyletic with both nuclear (Figs 3, 4) and mitochondrial data (Fig. 6), but lacks strong bootstrap support in the latter. Species relationships within this group are consistent and well supported for concatenated and coalescent UCE analyses (Figs 3, 4), and our presentation below follows this (accepted) phylogenomic structuring. Mitochondrial relationships among taxa within the group do not reflect UCE results, with many relatively low bootstrap support values (below 90) along the phylogenetic backbone; following arguments made in the Materials and methods we defer here to the nuclear phylogenomic results.

**Figure 12.**
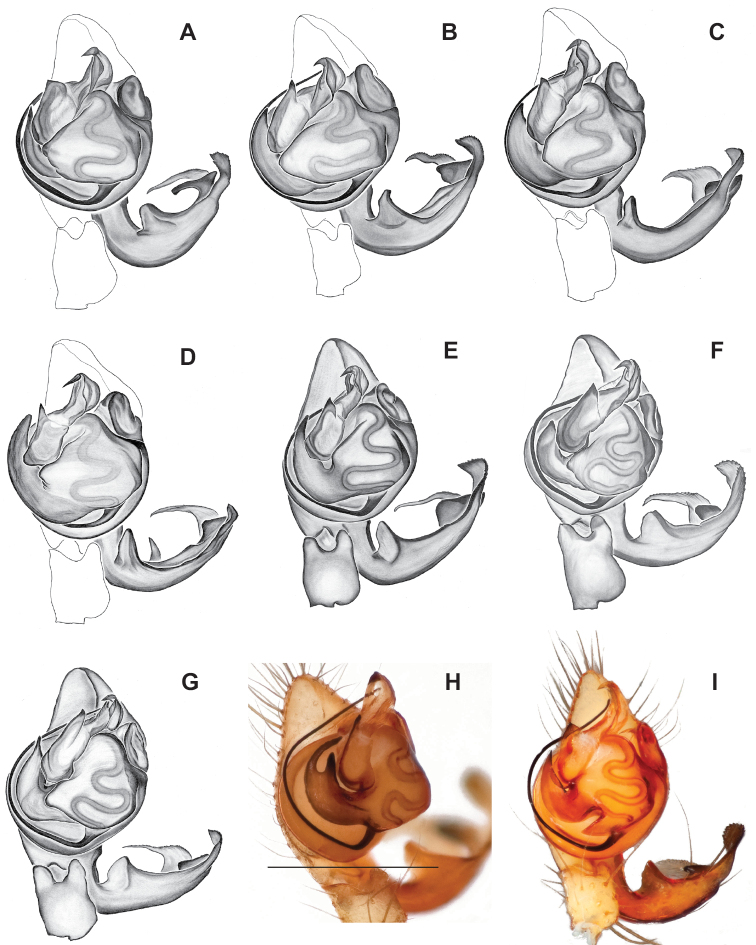
Comparative ♂ palps of *tennesseensis* group **A***Nesticussilvanus***B***N.cherokeensis***C***N.holsingeri***D***N.mimus***E***N.tennesseensis***F***N.dilutus***G***N.carolinensis***H***N.paynei*, and **I***N.roanensis*. All views are ventral. See subsequent figures for specimen locations and voucher details.

Male and female genital morphology suggests common ancestry for this complex of nine species, also defined as a species group by Gertsch (originally not including *Nesticuscherokeensis* sp. nov. or *N.roanensis* sp. nov.). Males of this species group include palps characterized by a translucent dorsal process of the paracymbium, projecting anteriorly then medially, with a thin medial projection with fine anterior serrations (Fig. 12A–G). Viewed dorsally, the dorsal process lies above a rectangular, dark paradistal process which itself lies above a narrower distal process with fine serrations along the anteroventral edge (e.g., Fig. 12F). Palps viewed ventrally include a shoe-shaped tegular apophysis, projecting distally behind the rectangular median apophysis (Fig. 12A–I). Noticeable variation among species is found in the shape of the base of the tegular apophysis (where the tegular apophysis projects from tegulum), the shape of the tegular apophysis itself, and the shape of the various paracymbial processes. Epigynal morphology is relatively conserved across species, with variation in the length and width of the epigynum and variation in spermathecal shape in certain taxa.

**Figure 13.**
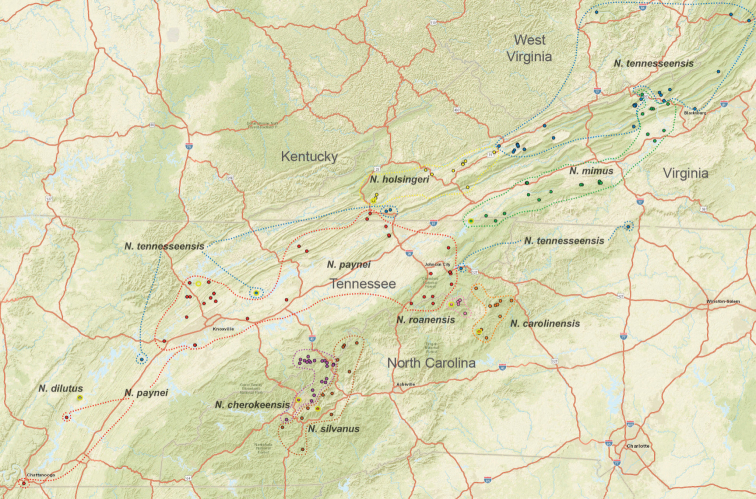
Distribution of *tennesseensis* group species. Type localities designated with yellow circles. State boundaries and major cities shown for geographic context. Dashed lines circumscribe known species distributions.

The *tennesseensis* group includes a combination of mostly cave-dwelling species distributed in the Appalachian Valley and Ridge (*Nesticusdilutus*, *N.holsingeri*, *N.mimus*), surface-dwelling species found entirely in the montane southern Blue Ridge (*N.carolinensis*, *N.cherokeensis*, *N.roanensis*, *N.silvanus*), and species found both in caves of the Valley and Ridge and mountains of the Blue Ridge (*N.tennesseensis*, *N.paynei*; see Fig. 13). The montane species *N.silvanus* and *N.cherokeensis* sp. nov. are successively sister to the remaining members of this species group, suggesting a south to north and mountain to cave biogeographic directionality (Fig. 13).

##### ﻿ Nesticus silvanus

Taxon classificationAnimaliaAraneaeNesticidae

Gertsch, 1984

9142AABF-CED1-5153-B35B-9EF9C11AA14E

Figs 14A–D , 15A–E

Nesticus silvanus Gertsch, 1984: 27, figs 141–143.

###### Material examined.

**Type material: *Holotype***: USA – **North Carolina, Jackson–Haywood Co.** • ♀ holotype; Water Rock Knob summit, elev. 1918 m, 30 Oct. 1969, W. Shear leg; AMNH; **New collections from type locality**: USA – North Carolina, Jackson Co. • 2♂, 7♀; vicinity of Water Rock Knob, off Blue Ridge Parkway; 35.4597°N, -83.1417°W; 9 Aug. 1992; M. Hedin leg; **Non type material**: – **Haywood Co.** • 2♂, 8♀; Blue Ridge Parkway, Mile 438, near Steestachee Bald overlook; 35.4263°N, -83.0388°W; 13 Aug. 1992; M. Hedin leg.; • 2♀; Cold Springs Creek, NE of I–40; 35.7585°N, -82.9938°W; 19 Aug. 2001; M. Hedin, M. Lowder leg.; MCH 01_137; • ♀, 1 imm; Fie Top Road, along Fie Creek; 35.5451°N, -83.1045°W; 3 Sep. 2002; M. Hedin, M. Lowder, P. Paquin leg.; MCH 02_187; • 3♀; Germany Cove Road, vicinity Hemphill Creek; 35.5543°N, -83.036°W; 25 Aug. 2005; M. Hedin, R. Keith, J. Starrett, S. Thomas leg.; MCH 05_087; • 2♀, 4 imm; NW Hebo Mountain, Hwy 209; 35.6869°N, -82.9065°W; 25 Aug. 2005; M. Hedin, R. Keith, J. Starrett, S. Thomas leg.; MCH 05_086; – **Jackson Co.** • 6♀; Dicks Creek, near Dicks Creek Church, N of Dillsboro; 35.4056°N, -83.2586°W; 31 Aug. 2002; M. Hedin, M. Lowder, P. Paquin leg.; MCH 02_173; • 11♀, 1 imm; Soco Creek, up Shut–in Creek road; 35.4653°N, -83.2148°W; 3 Sep. 2002; M. Hedin, M. Lowder, P. Paquin leg.; MCH 02_188; – **Macon Co.** • 2♀; Falls branch of Elijay Creek, 2 mi. E Elijay; 35.2135°N, -83.2535°W; 11 Aug. 1992; M. Hedin leg.; – **Madison Co.** • ♂, 12♀; Hwy 209, W Rocky Bluff campground at Long Mountain Branch; 35.8599°N, -82.8502°W; 19 Aug. 2001; M. Hedin, M. Lowder leg.; MCH 01_139; – **Swain Co.** • 14♀, 4 imm; Alarka Road, N Deep Gap church; 35.3482°N, -83.4064°W; 28 Aug. 2002; M. Hedin, M. Lowder, P. Paquin leg.; MCH 02_168; – **Tennessee, Cocke Co.** • 4♀; south of Round Mountain, Shelton Branch, Hwy 107; 35.835°N, -82.9519°W; 27 Aug. 2005; M. Hedin, R. Keith, J. Starrett, S. Thomas leg.; MCH 05_095; • 5♀; southeast of Round Mountain, W Rattlesnake Gap; 35.8472°N, -82.9443°W; 19 Aug. 2001; M. Hedin, M. Lowder leg.; MCH 01_138.

###### Diagnosis.

**Figure 14.**
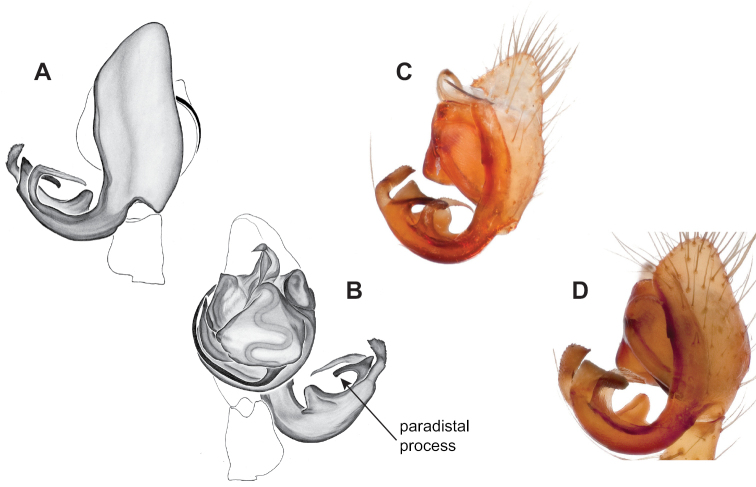
*Nesticussilvanus* ♂ palps. North Carolina, Jackson Co., vicinity of Water Rock Knob, MCH specimen #1080, dorsal (**A**), ventral (**B**) **C** North Carolina, Haywood Co., Blue Ridge Parkway, near Steestachee Bald overlook, MCH specimen #1145, dorsal **D** North Carolina, Madison Co., W of Rocky Bluff campground, MCH 01_139, dorsal. Scale bar: 0.5 mm.

**Figure 15.**
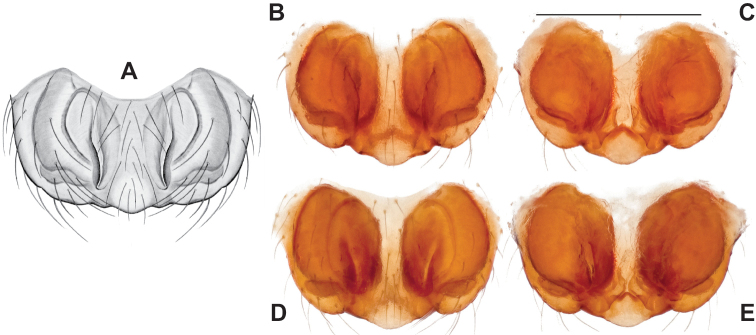
*Nesticussilvanus* epigynal variation **A** North Carolina, Jackson Co., vicinity of Water Rock Knob, MCH specimen #1083, ventral. North Carolina, Macon Co., Falls branch of Elijay Creek, MCH specimen #1115, ventral (**B**), dorsal (**C**) North Carolina, Madison Co., W of Rocky Bluff campground, MCH 01_139, ventral (**D**), dorsal (**E**). Scale bar: 0.5 mm.

**Figure 16.**
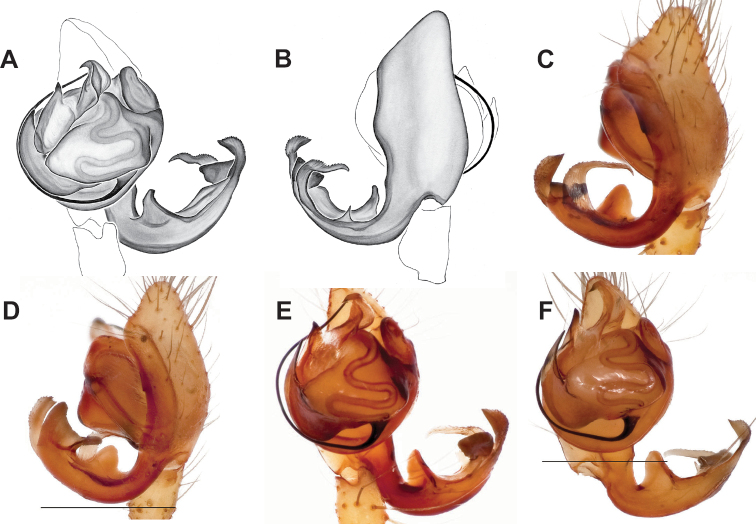
*Nesticuscherokeensis* sp. nov. ♂ palps. North Carolina, Swain Co., Blue Ridge Parkway, below Ballhoot Scar overlook, MCH specimen #1177, ventral (**A**), dorsal (**B**) **C** North Carolina, Haywood Co., S of Waterville, MCH 01_134, dorsal **D** North Carolina, Jackson Co., Blue Ridge Parkway, near Bunches Bald Tunnel, MCH specimen #1089, dorsal **E** North Carolina, Haywood Co., FR 288 above Pigeon River, MCH 01_136, ventral **F** North Carolina, Swain Co., road to Balsam Mountain, MCH 02_185, ventral. Scale bar: 0.5 mm.

**Figure 17.**
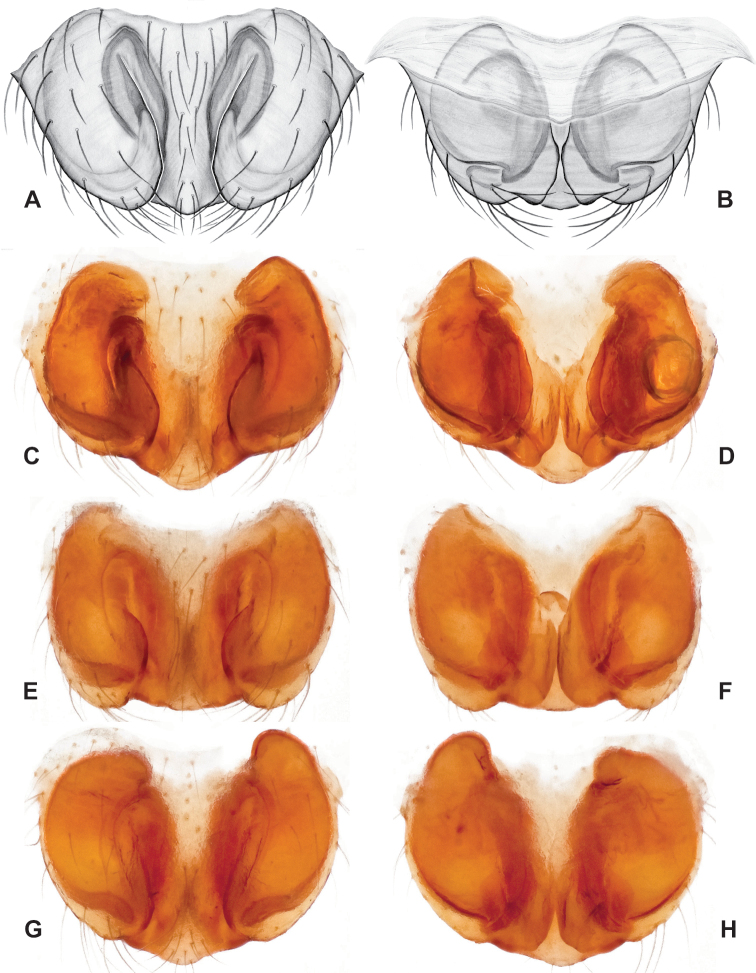
*Nesticuscherokeensis* sp. nov. epigynal variation. North Carolina, Swain Co., Blue Ridge Parkway, below Ballhoot Scar overlook, MCH specimen #1181, ventral (**A**), dorsal (**B**) North Carolina, Haywood Co., S of Waterville, MCH 01_134, ventral (**C**), dorsal (**D**) Tennessee, Cocke Co., S side of Indian Camp Creek, MCH specimen #1982, ventral (**E**), dorsal (**F**) North Carolina, Haywood Co., Cataloochee area, Sag Branch, ventral (**G**), dorsal (**H**). Scale bar: 0.5 mm.

Morphologically very similar to geographically parapatric *Nesticuscherokeensis* (Fig. 13). Males of *N.silvanus* can be distinguished from *N.cherokeensis* by the shape of the paradistal paracymbial process, which possesses a well-sclerotized ventral edge and a long prolateral extension (Fig. 14A–D). Also, the *N.silvanus* paracymbium lacks a basal projection of the dorsal process (Fig. 14A, C, D) as (sometimes) present in *N.cherokeensis* (Fig. 16B, F). Epigyna of *N.silvanus* are very similar to those of *N.cherokeensis* but (when viewed dorsally) possess epigynal plates with well separated medial margins, while in *N.cherokeensis* these plate margins are long, parallel, and touching (but see Fig. 15E vs. Fig. 17B). Additionally, *N.silvanus* epigyna possess anteriorly elongated epigynal pockets, lateral lobes that are shorter than the median septum, and relatively short spermathecae that lie perpendicular to the medium septum, separating them from remaining members of the *tennesseensis* group.

###### Description of previously undescribed ♂ from type locality

**(MCH specimen #1080).** Carapace light cream colored, gray pigmentation behind ocular area leading to midline and around edges. Legs pale yellow / cream. Abdomen with paired faint gray blotches on a light gray background. All eyes approximately equal in size, except for AMEs, ~ 1/3 width of ALEs. Eyes with rings of dark pigment. CL 1.39, CW 1.16, abdomen length 1.89, total body length 3.28. Leg I total length 8.66 (2.41, 0.54, 2.48, 2.25, 0.98), leg formula 1423, leg I / CW ratio 7.5. Paracymbium possesses a hook-shaped paradistal process with a well-sclerotized ventral edge and a long prolaterally-directed extension. Paracymbial dorsal process transparent and concave. Distal paracymbial process directed anteriorly, rounded, with a serrate edge. Ventral paracymbial process triangular with a blunted anterior edge. Median apophysis oval with a sharp anterior edge. Tegular process elongate, narrowing distally, and directed anteriorly. Nose-like bulge at the base of the tegular apophysis. Distal tip of conductor bent and directed prolaterally.

###### Variation.

Minimal palpal variation was observed for males from three sample locations, the dorsal paracymbial process in a single Rocky Bluff male being slightly wider and shorter (Fig. 14A–D). Female genitalic variation across sample locations was minimal (Fig. 15A–E).

###### Distribution and natural history.

Originally recorded from three locations (Gertsch 1984), now known to be relatively broadly distributed in appropriate surface microhabitats, including high-elevation habitats above 1900 m (e.g., Water Rock Knob, Steestachee Bald, etc.). This species is closely parapatric with *Nesticuscherokeensis* directly to the west, with an almost parallel geographic distribution (Fig. 13).

Strong phylogeographic structuring is observed in the mitochondrial data with a well-supported subclade found east of the Pigeon River (FieTop, Hebo Mtn, Rocky Bluff, etc.; Fig. 6), suggesting a possible role for riverine barriers in phylogeographic structuring, and further suggesting a southwest to northeast biogeographic directionality.

As an example of natural history, one male and 12 females were collected from rocky void spaces in a moist, rocky ravine near Rocky Bluff campground (MCH 01_139) during a 30-minute devoted *Nesticus* search.

###### Remarks.

This species is strongly supported as sister to remaining members of the *tennesseensis* group based on UCE evidence (Figs 3, 4).

##### ﻿ Nesticus cherokeensis sp. nov.

Taxon classificationAnimaliaAraneaeNesticidae

6B2AD477-0EEF-5D7B-B14A-BC4220E1348D

https://zoobank.org/6057E5AC-B191-4964-B829-A347E2B74B0D

Figs 16A–F , 17A–H

###### Material examined.

**Type material: *Holotype***: USA – **North Carolina, Swain Co.** • ♂; Blue Ridge Parkway, below Ballhoot Scar overlook near Ravensford; 35.5167°N, -83.2837°W; 9 Aug. 1992; M. Hedin leg.; (MCH specimen #1177). ***Paratypes***: – **North Carolina, Swain Co.** • ♂, 10♀; Blue Ridge Parkway, below Ballhoot Scar overlook near Ravensford; 35.5167°N, -83.2837°W; 9 Aug. 1992; M. Hedin leg.; (MCH specimens #1176, #1178–1187). **Non type material**: – **North Carolina, Haywood Co.** • 2♂, 3♀; Dogwood Flats Creek, W Longarm Mountain; 35.7201°N, -83.0731°W; 18 Aug. 2001; M. Hedin, M. Lowder leg.; MCH 01_135; • ♂, 12♀; Flat Branch Creek of Mt Sterling Creek, south of Waterville; 35.7407°N, -83.0741°W; 18 Aug. 2001; M. Hedin, M. Lowder leg.; MCH 01_134; • 2♀; Flat Branch Rd, SE Mt. Sterling, along Laurel Creek; 35.7526°N, -83.0895°W; 12 Aug. 2004; M. Hedin, R. Keith, J. Starrett, S. Thomas leg.; MCH 04_046; • ♂, 3♀; FR 288 above Pigeon River; 35.726°N, -83.0265°W; 18 Aug. 2001; M. Hedin, M. Lowder leg.; MCH 01_136; • ♀; FR 288 along Pigeon River, 0.4 mi. SW of I–40; 35.7308°N, -83.025°W; 27 Aug. 2005; M. Hedin, R. Keith, J. Starrett, S. Thomas leg.; MCH 05_094; • ♀, 4 imm; Great Smoky Mountains NP, Big Creek, 100 yards up Baxter Creek trail from picnic area; 35.7506°N, -83.1088°W; 17 Oct. 1994; F. Coyle leg.; • 3♀, 6 imm; Great Smoky Mountains NP, Boogerman Trail 0.5 mi from Northern end, N extension Den Ridge; 35.6225°N, -83.0847°W; 11 Sep. 1994; F. Coyle, J Miller leg.; • ♀; Great Smoky Mountains NP, Cataloochee area, Sag Branch, 1.5 mi from N end Caldwell Fork Trail; 35.6435°N, -83.0766°W; 10 Sep. 1994; F. Coyle, J Miller leg.; • ♂; Great Smoky Mountains NP, Cataloochee, 150 meters S mouth Palmer Branch at Caldwell Fork; 35.6251°N, -83.1121°W; 4 Jun. 1996; F. Coyle, Edwards, Stiles, Wright leg.; – **North Carolina, Jackson Co.** • ♂, ♀; Blue Ridge Parkway, Mile 460, near Bunches Bald Tunnel; 35.5092°N, -83.1883°W; 9 Aug. 1992; M. Hedin leg.; – **North Carolina, Swain Co.** • ♂, ♀; Great Smoky Mountains NP, 0.25 mi. NW Hientooga Overlook/Picnic Area on Hientooga Round Bottom Road; 35.5748°N, -83.1805°W; 3 Sep. 2002; M. Hedin, M. Lowder, P. Paquin leg.; MCH 02_186; • ♂, 4♀, 1 imm; Great Smoky Mountains NP, road to Balsam Mountain, N Black Camp Gap; 35.5437°N, -83.1679°W; 3 Sep. 2002; M. Hedin, M. Lowder, P. Paquin leg.; MCH 02_185; • ♂, ♀; Wesser Creek, Dills Road, S of Whittier; 35.3953°N, -83.3746°W; 18 Aug. 2007; M. Hedin, M. McCormack, S. Derkarabetian leg.; MCH 07_118; – **Tennessee, Cocke Co.** • ♂, 5♀; Great Smoky Mountains NP, above Cosby CG on Snake Den Ridge trail; 35.7432°N, -83.2218°W; 1 Aug. 1995; F. Coyle, Carbiener leg.; • ♀; Great Smoky Mountains NP, Cosby Ranger Station along Cosby Creek, behind ATBI residence house; 35.7779°N, -83.2135°W; 28 Jul. 2000; M. Hedin, J. Cokendolpher leg.; MCH 00_138; • ♂; Great Smoky Mountains NP, Maddron Bald trail to Albright Grove; 35.7608°N, -83.271°W; 3 Aug. 2000; M. Hedin, W. Reeves leg.; MCH 00_149; • ♂; Great Smoky Mountains NP, N side Gabes Creek at Gabes Mountain trail; 35.7523°N, -83.2419°W; 1 Aug. 1995; F. Coyle, Williams, Carbiener leg.; • 2♀; Great Smoky Mountains NP, near Cosby campground, below group camp parking area; 35.7533°N, -83.2066°W; 27 Aug. 2005; M. Hedin, R. Keith, J. Starrett, S. Thomas leg.; MCH 05_097; • ♀; Great Smoky Mountains NP, S side Indian Camp Creek on Maddron Bald Trail; 35.7378°N, -83.2777°W; 16 Apr. 1994; M. Hedin, B. Dellinger leg.; • 3♂, 4♀; Great Smoky Mountains NP, trail from Cosby to Low Gap; 35.7453°N, -83.197°W; 1 Aug. 2000; M. Hedin leg.; MCH 00_145; • 2♀; Great Smoky Mountains NP, trail from Low Gap to Mt. Cammerer; 35.754°N, -83.1658°W; 1 Aug. 2000; M. Hedin leg.; MCH 00_146.

###### Diagnosis.

As discussed above, this species is morphologically most similar to geographically adjacent *Nesticussilvanus*. Males have a fan-shaped paradistal paracymbial process (Fig. 16A–F) that lacks the elongate retrolateral extension and well-sclerotized ventral edge found in *N.silvanus*. Epigyna are very similar to that of *N.silvanus*, but when viewed dorsally possess adjacent medial plate margins that are parallel to each other, unlike the indistinct or pointed margins in *N.silvanus* (but see Fig. 15E. vs. Fig. 17B).

###### Description of ♂ holotype

**(MCH specimen #1177).** Carapace cream colored, gray pigmentation behind ocular area leading to midline and around edges. Legs pale yellow / cream. Abdomen with paired gray blotches on a light gray background. All eyes approximately equal in size, except for AMEs, ~ 1/2 width of ALEs. Eyes ringed with dark pigment. CL 1.45, CW 1.27, abdomen length 1.77, total body length 3.22. Leg I total length 10.55 (2.96, 0.54, 3.18, 2.8, 1.07), leg formula 1423, leg I / CW ratio 8.3. Paracymbium with a triangular ventral process with a sclerotized retrolateral edge, a dorsal process with an expanded serrate, distal portion, a heavily sclerotized triangular paradistal process, and a transparent, elongated, prolaterally directed dorsal process with a small triangular basal extension. Median apophysis a narrow oval with anteriorly directed edge coming to a point. Tegular process thick and sharp-tipped distally. Nose-like bulge at the base of the tegular apophysis. Distal tip of conductor bent and directed prolaterally.

###### ♂ Variation.

Males from different sample locations vary in the presence / absence of a small basal projection of the dorsal process (Fig. 16B, C).

###### Description of ♀ paratype

**(MCH specimen #1181).** Carapace cream colored, gray pigmentation behind ocular area leading to midline and around edges. Legs pale yellow / cream. Abdomen with paired dark gray blotches on a pale cream background. Eyes approximately equal in size, except for AMEs, ~ 1/2 width of ALEs. Eyes with rings of dark pigment. CL 1.51, CW 1.28, abdomen length 2.08, total body length 3.59. Leg I total length 9.27 (2.71, 0.58, 2.68, 2.23, 1.07), leg formula 1423, leg I / CW ratio 7.2. Epigynum possesses oval-shaped lateral lobes that extend to the posterior end of the median septum. Spermathecae visible beneath posterior lateral lobes, short and angled slightly upwards from perpendicular to septum. Viewed dorsally, large internal lobes extend anteriorly and possess sclerotized rims. Medial margins parallel to each other and touching along the midline.

###### ♀ Variation.

Females from different sample locations vary in the symmetry of the interior epigynal plates (Fig. 17C, G).

###### Distribution and natural history.

Found in rocky microhabitats from the rugged mountains of the eastern Great Smoky Mountains National Park, and adjacent eastern and southern locations (Fig. 13). The apparent gap at high elevations in this region (Fig. 13) likely reflects a lack of sampling in these less accessible high-elevation locations. Most collections include a modest number of specimens, suggesting a natural rarity for this taxon.

Along the Maddron Bald (along Indian Camp Creek) and the Low Gap to Mt. Cammerer trails (MCH 00_146) both *Nesticuscherokeensis* and *N.binfordae* sp. nov. were collected, indicating that these species are syntopic or nearly so at these locations. At both locations multiple collections were taken along an elevational transect and unfortunately lumped into a single collecting event. It is therefore not possible to discern if different species were collected at the exact same location (truly syntopic) or were closely parapatric along these elevational transects.

###### Etymology.

The species epithet (*cherokeensis*) honors the larger Cherokee Nation whose ancestral homelands included the mountains of western North Carolina. *Nesticuscherokeensis* can also be found near The Qualla Boundary, home of the Eastern Band of Cherokee.

###### Remarks.

This species is strongly supported as sister to remaining members of the *tennesseensis* group based on UCE evidence (Figs 3, 4). Mitochondrial structuring is very pronounced, with each sample location (or set of adjacent locations) genetically distinct (Fig. 6).

##### ﻿ Nesticus holsingeri

Taxon classificationAnimaliaAraneaeNesticidae

Gertsch, 1984

64203252-2D78-5347-8812-D503D29D4709

Fig. 18A–G

Nesticus holsingeri Gertsch, 1984: 25, figs 66–67, 91–93.

###### Material examined.

**Type material: *Holotype***: USA – **Virginia, Scott Co.** • ♂ holotype; Pond Cave; 5 Nov. 1966; J. Holsinger, S. Taylor leg; AMNH; **New collections from type locality**: USA – **Scott Co.** • 2♂, 7♀; Pond Cave, Rye Cove; 6 Oct. 1993; M. Hedin, C. Phillips leg; **Non type material**: – **Russell Co.** • ♂; Banners Corner Cave; 10 Apr. 2017; T. Malabad leg.; • ♂, 2♀; Bundys Cave No. 1, west of Lebanon, VA; 31 Jan. 2020; T. Malabad, R. Reynolds leg.; • ♂, ♀, 8 imm; Concrete Tank Cave; 24 Jul. 2017; T. Malabad leg.; • ♂, 3♀, 12 imm; Daugherty Cave, northeast of Lebanon, VA; 10 Apr. 2017; T. Malabad leg.; • 2♀; Daugherty Cave; 26 Jun. 2020; T. Malabad, A. Malabad leg.; • ♀; Ferrells Cave, northeast of Rosedale, VA; 26 Jun. 2020; T. Malabad, A. Malabad leg.; –**Scott Co.** • 4♂, 11♀; Alley Cave, E of Natural Tunnel SP; 19 Sep. 1992; M. Hedin, S. O’Kane leg.; • ♂; Alley Cave; 6 Oct. 1993; M. Hedin, C. Phillips leg.; • 3♂, ♀; Alley Cave; 22 Aug. 2005; M. Hedin, R. Keith, J. Starrett, S. Thomas leg.; MCH 05_072; • 2♀; Cox Ram Pump Cave; 3 Aug. 2016; T. Malabad leg.; – **Washington Co.** • 3♂, ♀; Brumley Creek, near Brumley Gap; 36.7933°N, -82.0229°W; 7 Aug. 2004; M. Hedin, R. Keith, J. Starrett, S. Thomas leg.; MCH 04_026; – **Wise Co.** • 4♂, 6♀; Burton’s Cave, SW of St Paul; 8 Oct. 1993; M. Hedin, C. Phillips leg.

###### Diagnosis.

**Figure 18.**
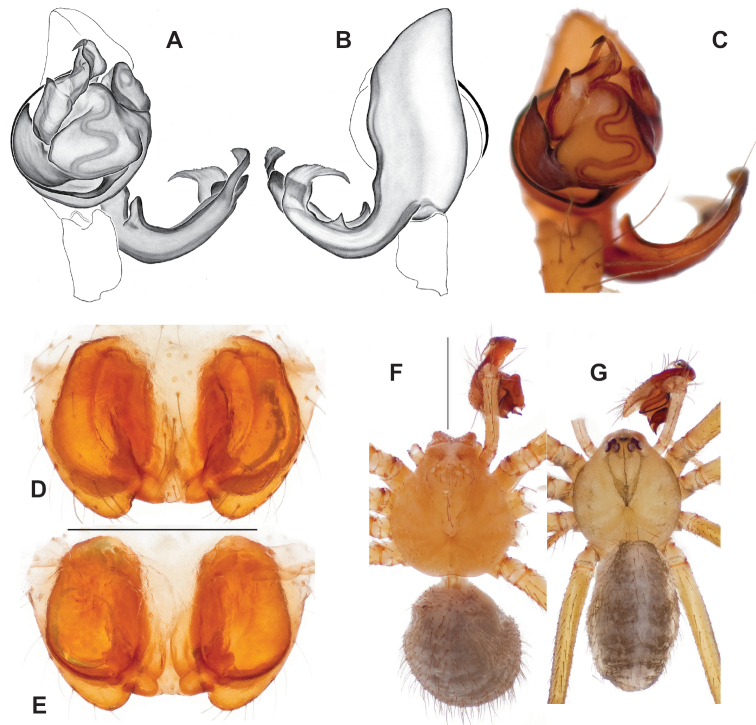
*Nesticusholsingeri*. Virginia, Scott Co., Pond Cave, MCH specimen #1780, ♂ palp, ventral (**A**), dorsal (**B**) **C** Virginia, Washington Co., Brumley Creek, MCH 04_026, ♂ palp, ventral. ♀ epigynum. Virginia, Washington Co., Brumley Creek, MCH 04_026, epigynum, ventral (**D**), dorsal (**E**). Scale bar: 0.5 mm. ♂ habitus images **F** Virginia, Washington Co., Brumley Creek, MCH 04_026 **G** Virginia, Scott Co., Pond Cave, MCH specimen #1780. Scale bar: 1 mm.

**Figure 19.**
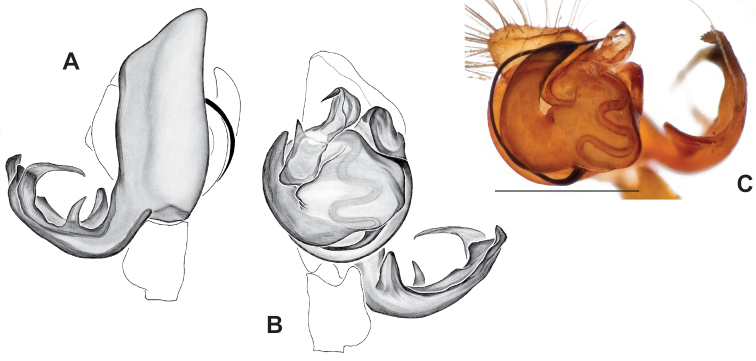
*Nesticusmimus* ♂ palps. Virginia, Giles Co., Straley’s Cave #1, MCH specimen #1396, dorsal (**A**), ventral (**B**) **C** Tennessee, Johnson Co., Backbone Rock Recreational Area, MCH 04_025, ventral. Scale bar: 0.5 mm.

**Figure 20.**
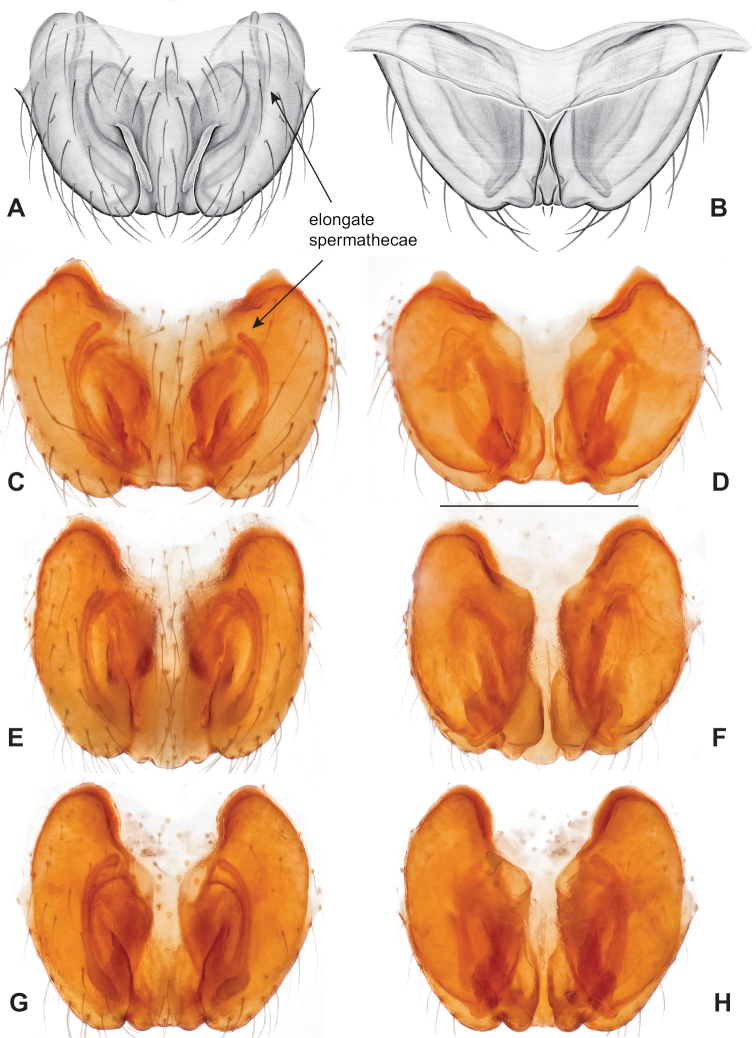
*Nesticusmimus* epigynal variation. Virginia, Giles Co., Straley’s Cave #1, MCH specimen #1399, ventral (**A**), dorsal (**B**). Virginia, Washington Co., Neal’s Sinks, MCH specimen #1430, ventral (**C**), dorsal (**D**). Virginia, Smyth Co., near Hurricane CG, MCH 07_087, ventral (**E**), dorsal (**F**). Tennessee, Johnson Co., Backbone Rock Recreational Area, MCH 04_025, ventral (**G**), dorsal (**H**). Scale bar: 0.5 mm.

Strongly supported by both mitochondrial and UCE data as sister to *Nesticusmimus*. The male tegular apophysis of *N.holsingeri* is shorter (nearly as wide as long) with a more pronounced narrowing tip (Fig. 18A, C) than in *N.mimus* (Fig. 19B, C). *Nesticusholsingeri* epigyna are more squarish and lack conspicuous elongate spermathecae (Fig. 18D, E), as compared to longer (anterior to posterior) *N.mimus* epigyna with conspicuous elongate spermathecae (Fig. 20A–H).

###### Variation.

This species exhibits interesting variation in somatic morphology. Specimens from Pond Cave are pale and long-legged with reduced eye pigmentation, specimens from Burton’s Cave and Alley Cave are pale and long-legged but with well-developed eyes, and specimens from the surface Brumley Creek population exhibit an “epigean” habitus, with dark abdomens, shorter legs, and well-developed eyes (Fig. 18F, G). Minimal genitalic variation was observed across sample locations.

###### Distribution and natural history.

Known only from a small area in southwestern Virginia, in the upper Clinch River drainage basin (Fig. 13). All known records are from limestone caves, except for the Brumley Creek population where spiders were collected from under surface rocks in a moist, north-facing stream valley. This species has been collected in near syntopy with *Nesticuscarteri* at Alley Cave, Virginia. At this site *N.carteri* is found under rocks in talus in a sink leading to the cave entrance, whereas *N.holsingeri* has only been collected from the dark zone of the cave.

###### Remarks.

The variation in degree of troglomorphy within this single species suggests that this character suite (eye development, pigmentation, leg length, etc.) can evolve relatively rapidly, as seen in other cave spider taxa (e.g., Arnedo et al. 2007).

##### ﻿ Nesticus mimus

Taxon classificationAnimaliaAraneaeNesticidae

Gertsch, 1984

E46A332C-2967-52CE-BA1A-51F73CE215CC

Figs 19A–C , 20A–H

Nesticus mimus Gertsch, 1984: 26, figs 64, 65.
Nesticus tennesseensis Gertsch, 1984: 24, 25 (in part).

###### Material examined.

**Type material: *Holotype*: USA** – **Virginia, Washington Co.** •♂ holotype; Shiloh School Cave, SE of Abingdon; 25 Nov. 1960; C.W. Greever leg; AMNH. **Non type material: USA** – **Tennessee, Johnson Co.** • ♂, 3♀; Backbone Rock Rec Area, 4 mi. S Damascus, off Hwy 133; 36.594°N, -81.8163°W; 7 Aug. 2004; M. Hedin, R. Keith, J. Starrett, S. Thomas leg.; MCH 04_025; **Tennessee, Sullivan Co.** • ♂, ♀; Potter’s Cave, S Abingdon; 14 Jul. 1979; J.R. Holsinger et al. leg.; AMNH; – **Virginia, Giles Co.** • 2♀, 26 imm; Curve Saltpetre Cave; 14 May. 2019; T. Malabad leg.; • ♀; Harris Cave, S of Pearisburg; 29 Jun. 1974; J. Holsinger, L. Ferguson leg; AMNH; • ♀, 2 imm; Harris Cave; 14 Sep. 2018; T. Malabad leg.; • 3♀, 7 imm; Spruce Run Mountain Cave; 14 Nov. 2018; T. Malabad leg.; • 2 imm; Straley’s Cave No. 1; 6 Sep. 1958; T.C. Barr leg; AMNH; • 2♂, 3♀; Co. Straley’s Cave No. 1 off Eggleston Road, S Pembroke; 17 Sep. 1992; M. Hedin, S. O’Kane leg.; • ♂, 7♀; Straley’s Cave No. 1 off Eggleston Road, S Pembroke; 10 Oct. 1993; M. Hedin, C. Phillips leg.; • ♀; Straleys Cave No. 2; 12 Sep. 2018; T. Malabad leg.; – **Pulaski Co.** • 4♀, 31 imm; Maze Cave; 25 Sep. 2018; T. Malabad leg.; • 2♀; Mebane Saltpetre Cave; 3 Oct. 2018; T. Malabad leg.; – **Radford Co.** • ♀, 3 imm; Adams Cave; 11 Apr. 2019; T. Malabad leg.; – **Smyth Co.** • 3♂, 6♀; Cow Shelter Cave, SE of Sugar Grove; 6 Oct. 1993; M. Hedin, C. Phillips leg.; • ♂, ♀; McMullin Cave, southwest of Marion, VA; 11 Jul. 2019; T. Malabad leg.; • ♀; McMullin Cave; 10 Sep. 2019; T. Malabad, K. Kosič Ficco leg.; • 2♀; McMullin Cave; 22 Oct. 2019; T. Malabad, K. Kosič Ficco leg.; • 9♂, 20♀; McMullin Cave; 2 Mar. 2020; T. Malabad, K. Kosič Ficco leg.; • ♂, 3♀; McMullin Cave; 23 Jun. 2020; T. Malabad, A. Malabad leg.; • 3♀; Mt. Rogers National Rec Area, 0.5 mi S of Hurricane CG on NF 84; 36.7186°N, -81.4911°W; 11 Aug. 2007; M. Hedin, R. Keith leg.; MCH 07_087; • ♀, 3 imm; Rowland Creek Cave; 10 Aug. 2018; T. Malabad leg.; • 8♂, 7♀, 1 imm; Whitetop Laurel Creek, Hwy 58, E of Damascus; 36.637°N, -81.75°W; 31 May. 2016; M. Hedin, S. Derkarabetian, J. Starrett, D. Proud leg.; MCH 16_034; – **Washington Co.** • 7♂, 9♀; Neal’s Sinks, 1.5 mi S Alvarado, Sweet Hollow Road; 18 Sep. 1992; M. Hedin, S. O’Kane leg.; – **Wythe Co.** • ♂, 4♀, 12 imm; Canyon Cave No. 1; 21 Sep. 2018; T. Malabad leg.; • ♂, ♀, 1 imm; Deep Spring Cave; 21 Sep. 2018; W. Orndorff leg.; • 6 imm; Deep Spring Cave; 7 Jul. 2018; T. Malabad leg.; • 2♂, 2♀, 14 imm; Mockleys Cave; 20 Sep. 2018; T. Malabad leg.; • ♂, ♀, 4 imm; Sinking Spring Cave No. 1; 21 Sep. 2018; T. Malabad leg.; • ♀, 7 imm; Sinking Spring Cave No. 2; 21 Sep. 2018; T. Malabad leg.; • 2♀, 6 imm; Speedwell Cave No. 1; 20 Sep. 2018; T. Malabad leg.

###### Diagnosis.

Strongly supported by both mitochondrial and UCE data as sister to *Nesticusholsingeri*. In *N.mimus* the basal dorsal process of the paracymbium is longer (Fig. 19A, B) and the tegular apophysis is relatively long and narrow, almost reaching the pronounced acute distal process of the median apophysis (Fig. 19B, C). Females possess a relatively elongate, narrow epigynum that is distinctive in the species group in possessing long, thin spermathecae that viewed ventrally bow upwards around the outer edge of the lateral pockets (Fig. 20A–H).

###### Description of previously undescribed ♀

**(MCH specimen #1398).** Carapace light cream colored, faint gray pigmentation behind ocular area leading to midline. Legs pale yellow / cream. Abdomen concolorous light cream. All eyes approximately equal in size, except for AMEs, ~ 1/4 width of ALEs. Eyes with rings of dark pigment. CL 1.37, CW 1.19, abdomen length 1.89, total body length 3.26. Leg I total length 8.68 (2.36, 0.58, 2.5, 2.22, 1.02), leg formula 1423, leg I / CW ratio 7.3. Epigynum relatively elongate and narrow. Lateral lobes well-defined with internal edges that extend posteriorly to the end of median septum or slightly further. Spermathecae extremely long and curved around fovea to anterior edge of epigynum. Large internal lobes (viewed dorsally) extend anteriorly with sclerotized rims. Medial margins parallel to each other but not touching along midline.

###### Variation.

No noteworthy variation in male or female genitalia was found across sample locations.

###### Distribution and natural history.

Known only from a small area of the Appalachian Valley and Ridge in southwestern Virginia and adjacent eastern Tennessee (Fig. 13). Found in both caves and moist, dark near-surface microhabitats. As an example of the near-surface natural history, eight males and seven females were collected along Whitetop Laurel Creek (MCH 16_034) from rock piles in a rich, rocky streamside forest.

###### Remarks.

Gertsch (1984) incorrectly identified specimens from the following locations as *Nesticustennesseensis*: Straley’s Cave No. 1 (2 immatures), Harris Cave (1 ♀), and Potter’s Cave (1 ♂, 1 ♀) – specimens from these populations belong to *N.mimus*. Gertsch (1984) also provisionally identified specimens from two montane locations as *N.mimus*: a single female from Table Rock Mountain (Burke County, NC), and a male specimen from Grandfather Mountain (cited as Watauga County, NC but label reads Avery County, NC). We contend that specimens from both locations correspond to *N.carolinensis* (see further comments below).

Specimens from Hedin (1997b) referred to as *Nesticus* “nov sp 1” (from Neal’s Sinks, Straley’s Cave No. 1, and Cow Shelter Cave) are actually *N.mimus*, and those referred to as *N.mimus* (Grandfather Mtn, Linville Gorge) are actually *N.carolinensis*.

##### ﻿ Nesticus tennesseensis

Taxon classificationAnimaliaAraneaeNesticidae

(Petrunkevitch, 1925)

268D87D2-F4DE-58C9-96F2-140C6FC87350

Figs 21A–C , 22A–G

Ivesia tennesseensis Petrunkevitch, 1925: 321, pl. 20, figs 4, 7, 10.
Yvesella tennesseensis : Arndt 1928: 84.
Ivesia tennesseensis : Bishop 1950: 10, figs 5–8.
Nesticus tennesseensis : Gertsch 1984: 23, figs 58–63, 82–84.

###### Material examined.

**New collections from type locality**: – **Tennessee, Grainger Co.** • 2♂, 2♀; Indian Cave, E of Blaine; 25 Sep. 1991; M. Hedin, K. Crandall leg.; • 3♂, 12♀; Indian Cave, E of Blaine; 21 Sep. 1992; M. Hedin, S. O’Kane leg.; • ♂, ♀, 5 imm; Indian Cave, TGA4; 22 Feb. 2014; M.L. Niemiller, A.S. Engel, S. Engel, A. Paterson leg.; MLN 14–010.7; **Non type material**: USA – **North Carolina, Surry Co.** • ♀, 1 imm; vic Fisher Peak lookout tower, SE of Blue Ridge Parkway; 36.559°N, -80.8276°W; 12 Aug. 2007; M. Hedin, R. Keith leg.; MCH 07_091; – **Tennessee, Carter Co.** • 3♀; off Hwy 167/321, near Watauga Lake, along Little Stony Creek; 36.309°N, -82.0732°W; 23 Aug. 2005; M. Hedin, R. Keith, J. Starrett, S. Thomas leg.; MCH 05_078; – **Tennessee, Roane Co.** • 5♂, ♀, 11 imm; Berry Cave, TRN3; 28 Jun. 2014; M.L. Niemiller, C.D.R. Stephen, A.S. Engel, et al. leg.; MLN 14–034.1; – **Virginia, Alleghany Co.** • ♂; Island Ford Cave, west of Low Moor, VA; 12 Jun. 2020; T. Malabad, P. Tegelman Malabad, K. Malabad, A. Malabad leg.; • 6♂, 7♀; Rumbold’s Cave, near Callaghan; 16 Sep. 1992; M. Hedin, S. O’Kane leg.; – **Virginia, Craig Co.** • 2♂, 4♀; Walkthrough Cave, sw of Newcastle; 10 Oct. 1993; M. Hedin, C. Phillips leg.; – **Virginia, Giles Co.** • 6♂, 7♀; Ballard’s Cave, just S Pearisburg; 9 Oct. 1993; M. Hedin, C. Phillips leg.; • 2♂, 4♀; Dead Doe Cave; 1 Jul. 2019; T. Malabad leg.; • ♀; Doe Mountain Cave; 16 Aug. 2019; T. Malabad leg.; • ♂; Hodges Cave, 3.5 miles southwest of Pearisburg, VA; 26 Nov. 2019; T. Malabad, K. Kosič Ficco leg.; • ♀; Little Stony Creek, NE of Pembroke; 37.3565°N, -80.5921°W; 10 Oct. 1993; M. Hedin, C. Phillips leg.; • ♂; Mountain Lake Biological Station, Jefferson Nat Forest; 37.3755°N, -80.5158°W; 11 Jul. 2013; C Richart leg.; • 4♂, 10♀; Starne’s Cave, SW Pearisburg, Wilburn Valley; 10 Oct. 1993; M. Hedin, C. Phillips leg.; • ♂5 imm; Starnes Cave; 3 Jun. 2019; T. Malabad leg.; • ♂, 4♀, 15 imm; Yer Cave; 9 Aug. 2019; T. Malabad leg.; – **Virginia, Montgomery Co.** • 4♀, 1 imm; Hancock Blowhole Cave; 14 Dec. 2014; E. Koertge leg.; – **Virginia, Scott Co.** • ♀, 6 imm; Coley Cave #2; 15 Sep. 2015; W. Orndorff leg.; • ♀; Herrons Echo Hall; 4 Aug. 2016; T. Malabad leg.; – Virginia, Tazewell Co. • 6♂, 11♀; Cassell’s Farm Cave, Burkes Garden; 9 Oct. 1993; M. Hedin, C. Phillips leg.; • 2♀, 2 imm; Cauliflower Cave; 24 Oct. 2018; T. Malabad leg.; • 2 imm; Corkscrew Cave; 4 Mar. 2017; K. Kosič Ficco leg.; • ♀, 4 imm; Corkscrew Cave; 27 Oct. 2018; T. Malabad leg.; • 4♂, 8♀; Fallen Rock Cave, Ward Cove, S Maiden Spring; 9 Oct. 1993; M. Hedin, C. Phillips leg.; • ♂; Gillespie Water Cave, southwest of Liberty, VA; 9 Sep. 2019; T. Malabad, K. Kosič Ficco, A. Futrell leg.; • ♂, 4♀, 9 imm; Glenwood Church Cave; 24 Oct. 2018; T. Malabad leg.; • ♀, 5 imm; Gulley Cave; 22 Jul. 2019; T. Malabad leg.; • 2♀, 3 imm; Little River Cave; 29 Nov. 2018; T. Malabad leg.; • 3♀, 4 imm; Lost Mill Cave 1; 22 Jul. 2019; T. Malabad leg.; • 2♀, 2 imm; Lost Mill Cave 2; 22 Jul. 2019; T. Malabad leg.; • 2♀; Stompbottom Cave, southeast of Claypool Hill, VA; 5 May. 2021; T. Malabad, K. Kosič Ficco, M. Ficco leg.; • ♂, 3♀; Stonley Cave; 10 Jan. 2019; T. Malabad leg.; – **West Virginia, Raleigh Co.** • ♂, 5♀; Grandview State Park, New River Gorge; 37.8321°N, -81.0614°W; 15 Sep. 1992; M. Hedin, S. O’Kane leg.

###### Diagnosis.

**Figure 21.**
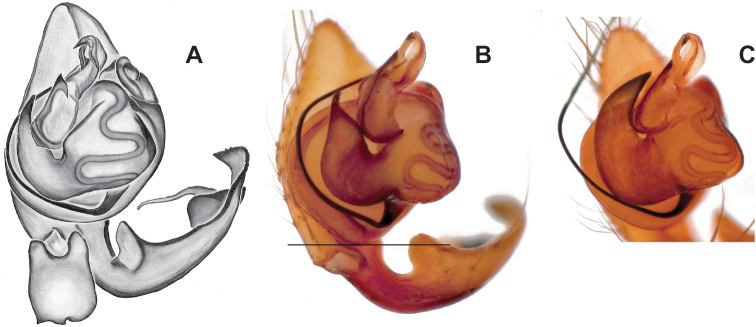
*Nesticustennesseensis* ♂ palps, ventral view **A** West Virginia, Raleigh Co., Grandview SP, MCH specimen #1360 **B** Virginia, Tazewell Co., Fallen Rock Cave, MCH specimen #1808 **C** Fallen Rock Cave, MCH specimen #1807. Scale bar: 0.5 mm.

**Figure 22.**
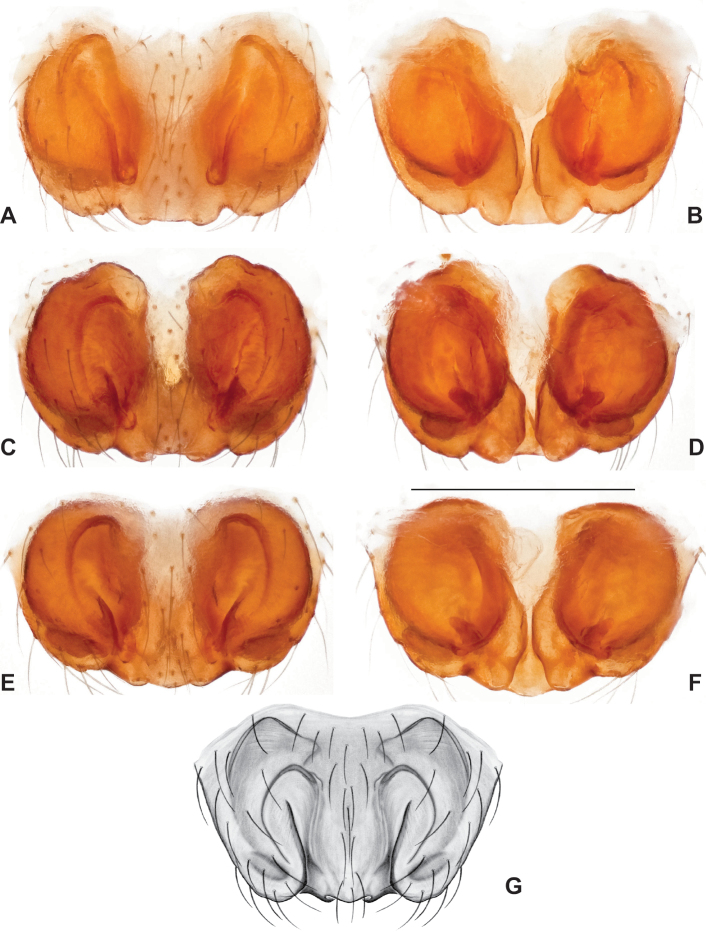
*Nesticustennesseensis* epigynal variation. Tennessee, Grainger Co., Indian Cave, MCH specimen #1010, ventral (**A**), dorsal (**B**). North Carolina, Surry Co., near Fisher Peak, MCH specimen #N1126, ventral (**C**), dorsal (**D**). Tennessee, Carter Co., near Watauga Lake, MCH 05_078, ventral (**E**), dorsal (**F**). West Virginia, Raleigh Co., Grandview SP, MCH specimen #1362, ventral (**G**).

*Nesticustennesseensis* and *N.dilutus* are morphologically similar sister species. Male differences are noted in the Diagnosis of *N.dilutus* below. Male *N.tennesseensis* may be differentiated from other members of this species group by the combination of palps with a paracymbium with a wide, broad ventral process, rectangular paradistal paracymbial process, a rectangular median apophysis with an anteriorly-pointed sclerotized edge, and a narrow, singularly-pointed tegular apophysis that extends to ~ half the length of the median apophysis (Fig. 21A–C). Females may be differentiated from other members of this species group by an overall rounded epigynum with short, somewhat globular spermathecae that extend perpendicular to the median septum (Fig. 22A–G). Viewed dorsally, circular pockets lie above extended parallel separated medial margins that diverge posteriorly.

###### Variation.

The shape of the tegular apophysis varies slightly across sample locations. Specimens from most locations (similar to type material from Indian Cave) possess a tegular apophysis with a broad, L-shaped base and an acute tip (see Gertsch, 1984: fig. 58), whereas other specimens have a narrower base with a gradually tapering tip (Fig. 21A, C). Different specimens from the Fallen Rock Cave population exhibit both conditions (Fig. 21B, C). We observed only minor variation in epigyna (Fig. 22A–G), even for geographically disjunct southeastern Surry and Carter County populations (Figs 13, 22C–F).

###### Distribution and natural history.

Known from both limestone caves (both shallow and deeper situations) and dark, cool, relatively moist near-surface habitats (e.g., rock piles, shallow cliff caves). Most known populations are from caves in the upper-central Appalachian Valley and Ridge, with a few peripheral montane surface populations (e.g., Surry County, NC; Carter County, TN; Raleigh County, WV). The southeastern Surry and Carter County populations appear disjunct, separated from the remainder of the species’ range by regions occupied by other taxa in the species group (Fig. 13).

###### Remarks.

Gertsch (1984: 24–25) incorrectly identified specimens from the following locations as *Nesticustennesseensis*: Sensabaugh Saltpeter Cave (3 imm) and “Cave by Clinch River” (one ♀) specimens belong to *N.paynei*. Straley’s Cave No. 1 (2 imm), Harris Cave (1 ♀), and Potter’s Cave (1 ♂, 1 ♀) specimens belong to *N.mimus*.

##### ﻿ Nesticus dilutus

Taxon classificationAnimaliaAraneaeNesticidae

Gertsch, 1984

C665A04B-6907-536B-B347-854FA7568153

Fig. 23A–C

Nesticus dilutus Gertsch, 1984: 27, figs 94–96; Hedin and Dellinger 2005: 10, figs 13, 14.

###### Material examined.

**New collections from type locality**: USA – **Tennessee, Rhea Co.** • 2♂, 10♀; Grassy Creek Cave, south of Old Washington; 23 Aug. 1992; M. Hedin, J. Hedin leg.; **Non type material**: – **Rhea Co.** • ♀; Starve Rock Cave (TRH7); 26 Mar. 2016; K.S. Zigler, M.L. Niemiller, N. Mann leg.; KSZ 15–566.

###### Diagnosis.

**Figure 23.**
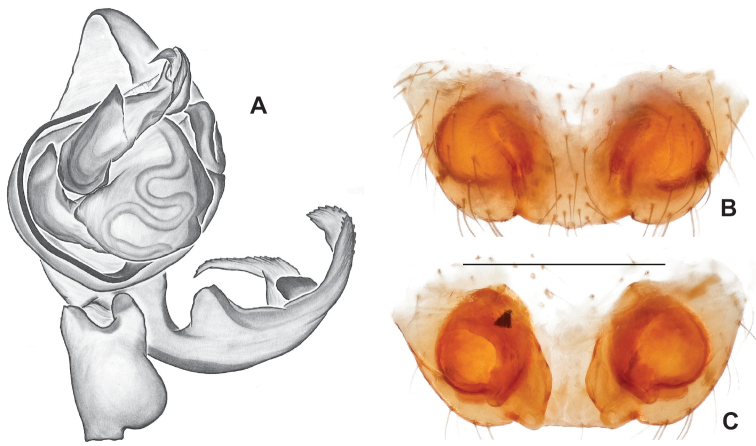
*Nesticusdilutus* ♂ and ♀. Tennessee, Rhea Co., Grassy Creek Cave, ♂ MCH specimen #1307, ventral (**A**). Tennessee, Rhea Co., Grassy Creek Cave, ♀ MCH specimen #1314, ventral (**B**), dorsal (**C**). Scale bar: 0.5 mm.

A close morphological and genetic relative of *Nesticustennesseensis*. This species differs most conspicuously from the former in that the basal, dorsal process of the paracymbium is absent (Hedin and Dellinger 2005, fig. 13). The tegular apophysis has a narrow, L-shaped base with a gradually tapering tip, although this condition is found in some northern populations of *N.tennesseensis* (see Fig. 21B). Epigyna very similar to *N.tennesseensis*, but possess more widely separated, pointed medial margins when viewed dorsally (Fig. 23B, C) rather than the extended parallel medial margins in *N.tennesseensis* (Fig. 22B, D, F), and the overall shorter (anterior to posterior) epigynal plate. More troglomorphic (depigmented, lacking median eyes, with proportionately long legs) than all known populations of *N.tennesseensis* (see Hedin and Dellinger 2005).

###### Variation.

The female specimen from Starve Rock Cave has an epigynum very similar to specimens from the type locality.

###### Distribution and natural history.

This troglomorphic taxon was previously known only from the type locality (Grassy Creek Cave), but is now known from two nearby caves in east-central Tennessee (Fig. 13). Starve Rock Cave is very near Grassy Creek Cave and may share a subterranean connection.

###### Remarks.

Sister to *Nesticustennesseensis* on UCE trees (Figs 3, 4), but *N.dilutus* sequences are embedded within a clade of *N.tennesseensis* sequences on the mitochondrial gene tree (Fig. 6). This latter pattern is attributed to either deep coalescence or gene tree estimation error.

##### ﻿ Nesticus carolinensis

Taxon classificationAnimaliaAraneaeNesticidae

(Bishop, 1950)

94CF9E4E-C8AC-51B6-82A1-E063826C8F24

**Figure 24.**
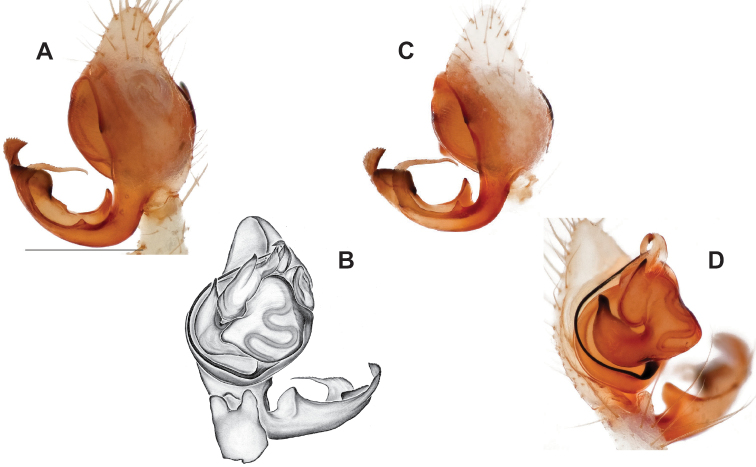
*Nesticuscarolinensis* ♂ palps. North Carolina, Avery Co., Elk River Cave, MCH 01_155, dorsal (**A**), ventral (**B**). North Carolina, McDowell Co., Linville Caverns, MCH specimen #1225, dorsal (**C**), ventral (**D**). Scale bar: 0.5 mm.

**Figure 25.**
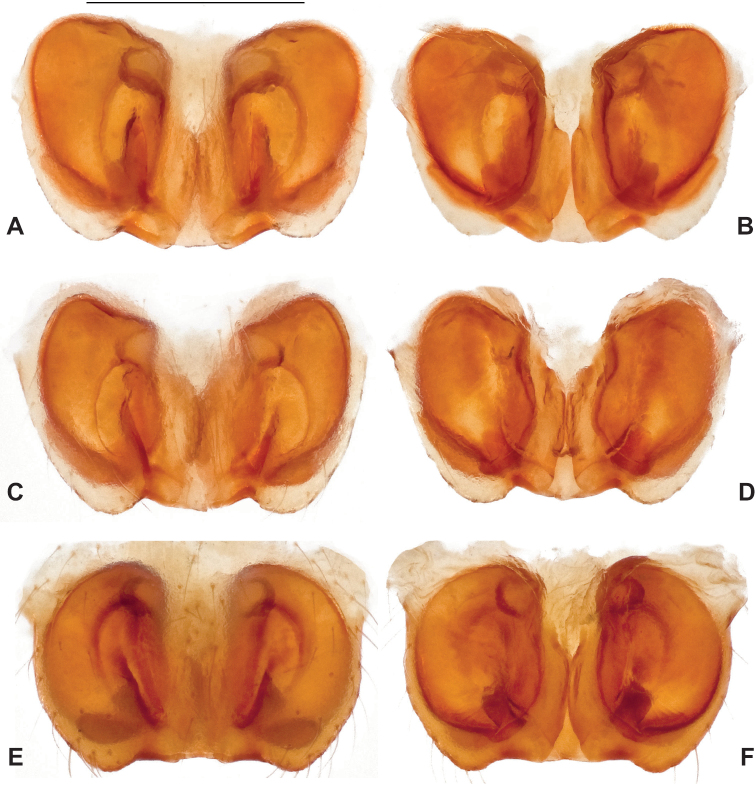
*Nesticuscarolinensis* epigynal variation. North Carolina, McDowell Co., Linville Caverns, MCH specimen #1227, ventral (**A**), dorsal (**B**). North Carolina, Avery Co., Elk River Cave, MCH 01_155, ventral (**C**), dorsal (**D**). North Carolina, Burke Co., Table Rock Mtn. (AMNH specimen), ventral (**E**), dorsal (**F**). Scale bar: 0.5 mm.

**Figure 26.**
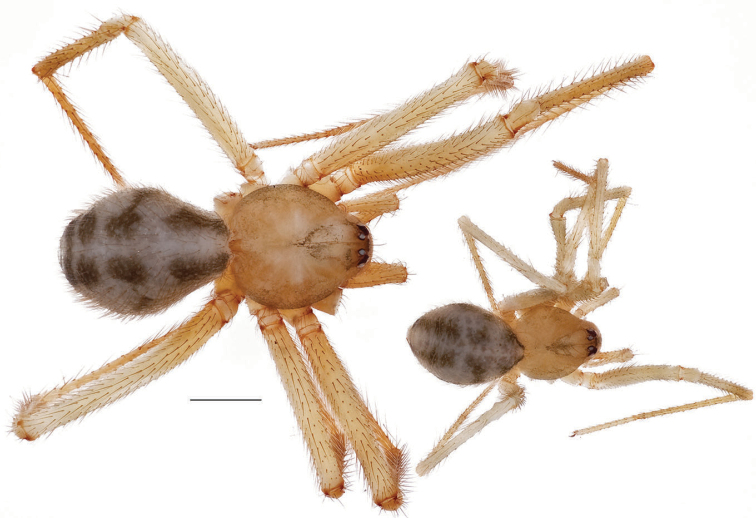
*Nesticuscarolinensis* habitus images, both adult females. North Carolina, Avery Co., Edgemont Road, MCH 01_163. Scale bar: 1 mm.

Figs 24A–D , 25A–F , 26

Ivesia carolinensis Bishop, 1950: 9, pl. 2, figs 1–4.
Nesticus carolinensis : Gertsch 1984: 25, figs 68–70, 88–90; Holler et al. 2020: 230.
Nesticus mimus : Gertsch 1984: 26, figs 85–87 (in part); Holler et al. 2020: 230.

###### Material examined.

**Type material: *Holotype***: USA – North Carolina, **McDowell Co.** • ♂ holotype; Linville Caverns, near Linville Falls; 6 Apr. 1947, S.C. Bishop leg.; AMNH; **Non type material**: – **Avery Co**. • ♂; upper slopes of Mt. Grandfather; 12 Oct. 1923; S.C. Bishop leg.; AMNH; • ♂, 6♀; Edgemont Road at Wilson Creek, 2 mi E Hwy 221; 36.0905°N, -81.8026°W; 24 Aug. 2005; M. Hedin, R. Keith, J. Starrett, S. Thomas leg.; MCH 05_080; • 11♀, 8 imm; Edgemont Road, 1 mile below Hwy 221; 36.0859°N, -81.815°W; 24 Aug. 2001; M. Hedin, M. Lowder leg.; MCH 01_163; • ♂, ♀, 3 imm; Elk River Cave, 1 mi S Elk River Falls; 36.1892°N, -81.9617°W; 22 Aug. 2001; M. Hedin, M. Lowder leg.; MCH 01_155; • 2♀, 7 imm; Roseboro Road past first crossing of Rockhouse Creek; 36.0192°N, -81.7813°W; 24 Aug. 2001; M. Hedin, M. Lowder leg.; MCH 01_164; • 3♂, 9♀; W side of Grandfather Mtn., 1 mi. NE Linville on Hwy 221; 36.0825°N, -81.8568°W; 16 Aug. 1992; M. Hedin leg.; – **Burke Co.** • ♀;Table Rock Mtn; 15 Jun. 1949, no collector information; AMNH; • 3♂, 4♀; Pine Gap Trail, W side of Linville Gorge, S of Linville Falls off Old NC 105; 35.9396°N, -81.9219°W; 16 Aug. 1992; M. Hedin leg.; • ♂, 4♀, 12 imm; Pine Gap Trail, W side of Linville Gorge, S of Linville Falls off Old NC 105; 35.9396°N, -81.9219°W; 25 Aug. 2001; M. Hedin, F. Coyle, M. Lowder, P. Paquin leg.; MCH 01_165; – **Caldwell Co.** • ♀, 1 imm; Burnt Field Branch Cave; 9 May. 1995; C. Holler, C. Holler leg.; • 4♂, 4♀, 3 imm; China Creek at FR 4071 crossing, SW of Blowing Rock; 36.1151°N, -81.6983°W; 24 Aug. 2001; M. Hedin, M. Lowder leg.; MCH 01_161; – **McDowell Co.** • 4♂, 10♀; Linville Caverns, S of Linville Falls, off Hwy 221N; 35.9189°N, -81.9393°W; 16 Aug. 1992; M. Hedin leg.; • 2♂, 9♀, 5 imm; Hwy 221N, N of Linville Caverns; 35.9268°N, -81.9385°W; 25 Aug. 2001; M. Hedin, F. Coyle, M. Lowder, P. Paquin leg.; MCH 01_166; • 2♀; off Hwy 221N, N Linville Caverns; 35.9317°N, -81.9391°W; 24 Aug. 2005; M. Hedin, R. Keith, J. Starrett, S. Thomas leg.; MCH 05_081; – **Watauga Co.** • 7 imm (identification based on mitochondrial evidence); Green Mountain, Hwy 221, crossing of Green Mountain Creek; 36.1142°N, -81.7782°W; 24 Aug. 2001; M. Hedin, M. Lowder leg.; MCH 01_162.

###### Diagnosis.

Males may be differentiated from other members of this species group by the combination of palps with a paracymbium with a wide, broad ventral process, the paradistal paracymbial process broad and triangular, a median apophysis that is a thin rectangle with an anterior sclerotized point, and a broad, singularly-pointed tegular apophysis that extends to ~ half the length of the median apophysis (Fig. 24A–D). Females may be differentiated from other members of this species group by epigyna with lateral lobes that are approximately equal to or slightly longer than the median septum, anteriorly-elongated epigynal pockets, and (viewed dorsally) touching parallel medial margins that diverge posteriorly (Fig. 25A–F).

###### Variation.

In males from different sample locations the distal tip of the tegular apophysis varies in shape from blunt (e.g., Grandfather Mtn, Edgemont Rd) to more fingerlike (e.g., N Linville Caverns, China Creek, Elk River Cave, etc.). This variation does not obviously follow geographic or phylogeographic (see below) lines. Females from different sample locations are relatively conservative in epigynal morphology (Fig. 25A–F), except for the AMNH specimen from Table Rock Mountain (see further comments below).

Fig. 26 shows an example of variation in adult female body size for specimens from a single collection location (from Edgemont Road, MCH 01_163), illustrating why we have not considered body size variation as particularly taxonomically important in this revision.

###### Distribution and natural history.

Previously known only from caves, but quite common and abundant in suitable near-surface habitats. Mostly from the uplands between the Linville and Grandfather Mountains of western North Carolina, northeast of the Asheville Basin (Fig. 13).

Strong phylogeographic structuring is observed in the mitochondrial data, with a well-supported subclade found east of the Linville Gorge (China Creek, Green Mountain, Elk River Cave, Rockhouse Creek, etc.; Fig. 6). This phylogeographic break also corresponds to a small sampling gap (Fig. 13), so isolation by distance (with incomplete sampling) vs. isolation by geography (e.g., the Linville Gorge) cannot be distinguished.

###### Remarks.

Gertsch (1984) provisionally attributed specimens from two montane locations to *Nesticusmimus*: a single female from Table Rock Mountain (Burke County, NC), which he described and illustrated, and a male specimen from Grandfather Mountain. The Grandfather Mountain male matches *N.carolinensis* specimens from our collections, for which we also collected DNA data. The female from Table Rock Mountain has a divergent epigynal morphology from *N.carolinensis* (wider than tall, short spermathecae, etc. Fig. 25E, F); we place the specimen here based mostly on geography, adjacent to our other Linville Gorge collections. It remains possible that the specimen is from north of Table Rock, closer to Watauga Lake (and locations for *N.tennesseensis*, see Fig. 22E, F).

Holler et al. (2020) cite new cave records from McDowell County. They also attribute Burnt Field Branch Cave specimens (Caldwell County) to *Nesticusmimus*, but we have examined females from this location and consider them to be *N.carolinensis*, lacking the unique spermathecae of *N.mimus*.

This species is supported as sister to *Nesticuspaynei* + *N.roanensis* with a 92% bootstrap and sCF value of 37.5 on the UCE concatenated maximum likelihood tree, and a lower local posterior probability value on the UCE ASTRAL species tree (Figs 3, 4).

##### ﻿ Nesticus paynei

Taxon classificationAnimaliaAraneaeNesticidae

Gertsch, 1984

360B8769-2AA9-572A-9170-0D35DE225415

Fig. 27A–K

Nesticus paynei Gertsch, 1984: 28, figs 153–155, 159–160.
Nesticus tennesseensis : Gertsch 1984: 26 (in part).

###### Material examined.

**Type material: *Holotype***: USA – **Tennessee, Anderson Co.** • ♂ holotype; Reeder’s Cave, 2 mi. N Clinton; 10 Mar. 1965; J.A. Payne leg.; AMNH. **Non type material**: USA – **North Carolina, Mitchell Co.** • 2♂, 3♀, 2 imm; Pigeonroost Creek, N of Nolichucky River; 36.0983°N, -82.2831°W; 21 Aug. 2001; M. Hedin, M. Lowder leg.; MCH 01_147; – **Tennessee, Anderson Co.** • 2♀; Norris Dam Cave, 2 mi. N Norris; 20 Sep. 1992; M. Hedin, S. O’Kane leg.; • 3♀; Norris Dam Cave; 6 Oct. 1993; M. Hedin, C. Phillips leg.; • ♂, 3♀, 2 imm; Rieders Lost Creek Cave, TAN36; 30 May. 2016; M.L. Niemiller, E.T. Carter, N.S. Gladstone leg.; MLN 16–027.13; • ♀, 5 imm; Springhill Saltpeter Cave, TAN3; 28 Oct. 2017; M.L. Niemiller, E.T. Carter, N.S. Gladstone, K.D.K. Niemiller, C. Kendall, L. Hayter, M.J. Ravesi leg.; MLN 17–012.5; • 3♀; Weaver Cave, TAN22; 22 Mar. 2016; M.L. Niemiller, C.D.R. Stephen leg.; MLN 16–022.10; • ♂, 7 imm; Weaver Cave, TAN22; 5 Mar. 2017; N.S. Gladstone leg.; NSG 17–TAN22.5; – **Tennessee, Carter Co.** • ♂, 6♀; Grindstaff Cave, near Braemar/Hampton; 18 Sep. 1992; M. Hedin, S. O’Kane leg.; • 5♀; Grindstaff Cave; 11 Oct. 1993; M. Hedin, C. Phillips leg.; • 2♂, 2♀; Grindstaff Cave; 22 Aug. 2001; M. Hedin, M. Lowder leg.; MCH 01_152; • ♂, ♀; Rockhouse Cave, TCR3; 14 May. 2014; A.S. Engel, A. Paterson, S.W. Jones, et al. leg.; ASE 14–CR3.4; • 15♀, 1 imm; Ingram Branch Road, W of Hwy 19E/37; 36.214°N, -82.1456°W; 9 Aug. 2004; M. Hedin, R. Keith, J. Starrett, S. Thomas leg.; MCH 04_033; • 8♀, 7 imm; Dennis Cove Road, first crossing of Black Mtn branch above Braemer; 36.2774°N, -82.1504°W; 22 Aug. 2001; M. Hedin, M. Lowder leg.; MCH 01_154; – **Tennessee, Hamilton Co.** • ♀; Clay Cave; 24 Feb. 2013; W.T. Coleman, L. Carver, K.S. Zigler leg.; – **Tennessee, Hancock Co.** • 2♂, 8♀; Cantwell Valley Cave, SW of Sneedville; 7 Oct. 1993; M. Hedin, C. Phillips leg.; • ♀; Hwy 31 on Clinch Mountain; 36.413°N, -83.2237°W; 21 Aug. 2005; M. Hedin, R. Keith, J. Starrett, S. Thomas leg.; MCH 05_068; – **Tennessee, Hawkins Co.** • 4♂, 4♀; Sensabaugh Saltpeter Cave, W of Kingsport; 7 Oct. 1993; M. Hedin, C. Phillips leg.; • 3 imm; Sensabaugh Saltpeter Cave; 15 Apr. 1967; J. Holsinger leg.; AMNH; – **Tennessee, Jefferson Co.** • ♂, 4♀; Tater Cave, TJF8; 3 Aug. 2015; M.L. Niemiller, E.T. Carter, A.S. Engel, L.E. Hayter, K.D. Kendall leg.; MLN 15–016.18; – **Tennessee, Knox Co.** • 3♀, 11 imm; Blowing Hole Cave; 16 May. 2013; K.S. Zigler, M.L. Niemiller leg.; MLN 13–003; • ♂, ♀, 12 imm; Keller Bend Cave; 16 May. 2013; K.S. Zigler, M.L. Niemiller leg.; MLN 13–004; • ♂, 2♀; Kirkpatrick Cave, TKN62; 9 Feb. 2014; M.L. Niemiller, A.S. Engel, S. Engel, C. Kerr leg.; MLN 14–009; • 2♀, 3 imm; Kirkpatrick Cave, TKN62; 6 Jul. 2014; M.L. Niemiller, A.S. Engel, A Paterson leg.; MLN 14–037.10; • ♀; Pedigoe Cave, TKN103; 14 Jul. 2018; N.S. Gladstone leg.; NSG 18–TKN103.8; • 3♂, 7♀; Roaring Springs Cave, W of Copper Ridge; 6 Oct. 1993; M. Hedin, C. Phillips leg.; • ♀; Watercress Cave, TKN153; 13 Jan. 2019; N.S. Gladstone leg.; NSG 19–TKN153.16; – **Tennessee, Loudon Co.** • 2♂, 7♀, 3 imm; Ghost Cave, TLN3; 30 Aug. 2014; M.L. Niemiller, C.D.R. Stephen leg.; MLN 14–043.13; – **Tennessee, Meigs Co.** • 3♀, 2 imm; Blythe Ferry Cave, TME; 26 Jan. 2018; M.L. Niemiller, D. Pelren, J. Traxley, C.L. Barber leg.; MLN 18–004.16; – **Tennessee, Roane Co.** • ♀; “Cave by Clinch River”, AEC controlled area; 31 Jan. 1953, AMNH; – **Tennessee, Sevier Co.** • 2♂, 5♀, 3 imm; Two County Cave, TSV36; 5 Jul. 2014; M.L. Niemiller, A.S. Engel, A. Paterson leg.; MLN 14–036.13; – **Tennessee, Sullivan Co.** • 4♀; Bays Mountain Park, W of Kingsport; 36.507°N, -82.6109°W; 10 Aug. 2007; M. Hedin, R. Keith leg.; MCH 07_085; • 2♂, 4♀; Eastman Recreation Area, Bays Mountain; 36.5029°N, -82.61°W; 8 Aug. 2004; M. Hedin, R. Keith, J. Starrett, S. Thomas leg.; MCH 04_029; • 4♀; Holston Mountain, Holston Mountain Road, 6 mi E Hwy 19E; 36.4328°N, -82.167°W; 23 Aug. 2005; M. Hedin, R. Keith, J. Starrett, S. Thomas leg.; MCH 05_077; – **Tennessee, Unicoi Co.** • 1 imm (identification based on mitochondrial evidence); road to Unaka Springs, along Nolichucky River, SW of Banner Hill; 36.0982°N, -82.4439°W; 22 Aug. 2007; M. Hedin, M. McCormack, S. Derkarabetian leg.; MCH 07_146; • 2♂, 4♀; Rock Creek Recreational Area, SE of Erwin; 36.1379°N, -82.3482°W; 9 Aug. 2004; M. Hedin, R. Keith, J. Starrett, S. Thomas leg.; MCH 04_032; • 2♂, 3♀; Unaka Mountains, Forest Road 230, NE Unaka Mountain; 36.1396°N, -82.2837°W; 9 Aug. 2004; M. Hedin, R. Keith, J. Starrett, S. Thomas leg.; MCH 04_035; – **Tennessee, Union Co.** • 2♀; Coppock Cave, near Central Valley School, S Ridenour; 25 Sep. 1991; M. Hedin, K. Crandall leg.; • 2♂, 9♀; Coppock Cave; 20 Sep. 1992; M. Hedin, S. O’Kane leg.; – **Virginia, Scott Co.** • ♀; Wininger Cave; D. Hubbard leg.; • 10♀; Wolfe Cave, off Rd 629, W Hwy 23, near Speer’s Ferry; 7 Oct. 1993; M. Hedin, C. Phillips leg.

###### Diagnosis.

**Figure 27.**
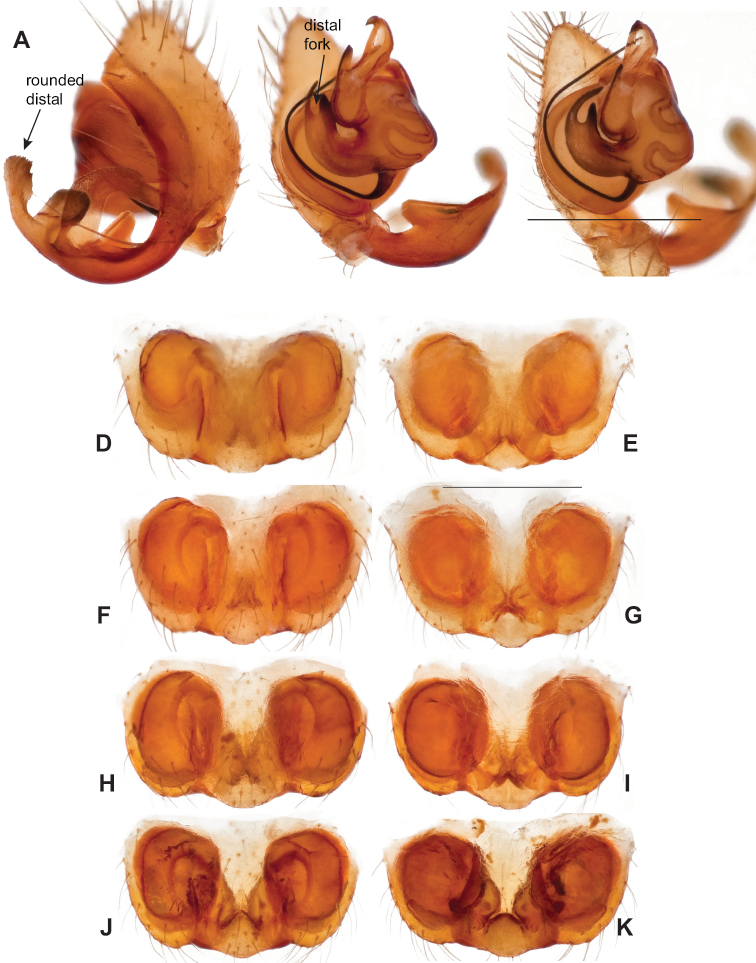
*Nesticuspaynei* genitalia. Tennessee, Hawkins Co., Sensabaugh Saltpeter Cave, MCH ♂ specimen #1762, dorsal (**A**), ventral (**B**) **C** Tennessee, Unicoi Co., Rock Creek Recreational Area, MCH 04_032, palp, ventral. Epigynal variation. Tennessee, Roane Co., cave by Clinch River (AMNH specimen), ventral (**D**), dorsal (**E**). Tennessee, Hawkins Co., Sensabaugh Saltpeter Cave, MCH specimen #1765, ventral (**F**), dorsal (**G**). Tennessee, Unicoi Co., Rock Creek Recreational Area, MCH 04_032, ventral (**H**), dorsal (**I**). Tennessee, Carter Co., Ingram Branch Road, MCH 04_033, ventral (**J**), dorsal (**K**). Scale bar: 0.5 mm.

Males are diagnosed from closely-related *Nesticusroanensis* by the distally split tegular apophysis (Fig. 27A, B), and from all other members of the species group by distal and dorsal paracymbial processes that are relatively rounded, vs. truncate. Females may be differentiated from other members of this species group (except *N.roanensis*) by epigyna with circular epigynal pockets, and internal plates (viewed dorsally) with touching medial margins (Fig. 27D–K).

###### Variation.

There is minor variation in the depth of the distally bifurcate tegular apophysis (e.g., slightly deeper in neighboring Pigeonroost Creek and Rock Creek Recreation Areas specimens), but in general populations are conspicuously homogeneous despite a large and fragmented geographic distribution (Fig. 13). Female epigynal variation is minimal (Fig. 27D–K).

###### Distribution and natural history.

Most sample locations are from limestone caves in the central part of the upper Tennessee River valley, near Knoxville, Tennessee, and extending northeast and southwest from there (Fig. 13). Some cave populations are highly disjunct, similar to the situation seen in *Nesticustennesseensis* and *N.carteri*. While all Gertsch (1984) records for *N.paynei* are from caves, many of the new records reported here are from rockpile habitats from the mountains along the North Carolina / Tennessee border near Johnson City, Tennessee (Fig. 13). Montane samples are early branching on the UCE ASTRAL tree (Fig. 4).

###### Remarks.

We identified spiders from Sensabaugh Saltpeter Cave (3 imm) and ‘Cave by Clinch River’ (one ♀) as *Nesticuspaynei*. These collections were originally identified by Gertsch (1984: 24–25) as *N.tennesseensis*.

As discussed directly below *Nesticuspaynei* is intermixed with *N.roanensis* on mitochondrial trees (Fig. 6), and these taxa are not strictly reciprocally monophyletic on concatenated UCE trees (Figs 3, 4). However, for reasons argued below we consider *N.roanensis* as distinct from *N.paynei*, consistent with our original “morphology first” hypothesis.

Because neither female morphology nor mitochondrial placement can strictly distinguish *Nesticuspaynei* from *N.roanensis*, our attribution for some female-only *N.paynei* collections from near Roan Mountain is necessarily tentative. This includes Ingram Branch and Dennis Cove Road collections from north of Roan Mountain (Fig. 13; Suppl. material 1); we provisionally place these as *N.paynei* as they occur at relatively low elevations. Male specimens and/or UCE data will be important to obtain for these locations.

##### ﻿ Nesticus roanensis sp. nov.

Taxon classificationAnimaliaAraneaeNesticidae

DA550429-7B4D-59F3-9D0B-439063F73C48

https://zoobank.org/AA97398C-1B2D-4E1D-B5DD-09C3DB072EFC

Fig. 28A–G

###### Material examined.

**Type material: *Holotype***: USA – **North Carolina, Mitchell Co.** • ♂; Roan Mountain, below Roan High Bluff; 36.0931°N, -82.1459°W; 22 Aug. 2001; M. Hedin, M. Lowder leg.; MCH 01_150 (SDSU_TAC000675); ***Paratype***: – **North Carolina, Mitchell Co.** • ♀; Roan Mountain, below Roan High Bluff; 36.0931°N, -82.1459°W; 22 Aug. 2001; M. Hedin, M. Lowder leg.; MCH 01_150 (SDSU_TAC000676); **Non type material**: USA – **North Carolina, Avery Co.** • ♂, 1 imm; Henson Creek at Henson Creek Baptist Church, on Henson Rd, N of Ingalls; 36.0374°N, -82.042°W; 21 Aug. 2007; M. Hedin, M. McCormack, S. Derkarabetian leg.; MCH 07_138; – **North Carolina, Mitchell Co.** • 2♂, 2♀; Roan Mountain, below Roan High Bluff; 36.0931°N, -82.1459°W; 22 Aug. 2001; M. Hedin, M. Lowder leg.; MCH 01_150; • ♂, 2♀, 6 imm; upper Roan Valley, Hwy 261; 36.0929°N, -82.0932°W; 21 Aug. 2001; M. Hedin, M. Lowder leg.; MCH 01_148; – **Tennessee, Carter Co.** • 5♂, 15♀; Hwy 143, NE Roan Mountain, 3 mi. N Carvers Gap; 36.1184°N, -82.0818°W; 9 Aug. 2004; M. Hedin, R. Keith, J. Starrett, S. Thomas leg.; MCH 04_034; • 3♂, 3♀; Hwy 143, NE Roan Mountain, 3 mi. N Carvers Gap; 36.1094°N, -82.0961°W; 31 May. 2016; M. Hedin, S. Derkarabetian, J. Starrett, D. Proud leg.; MCH 16_033.

###### Diagnosis.

**Figure 28.**
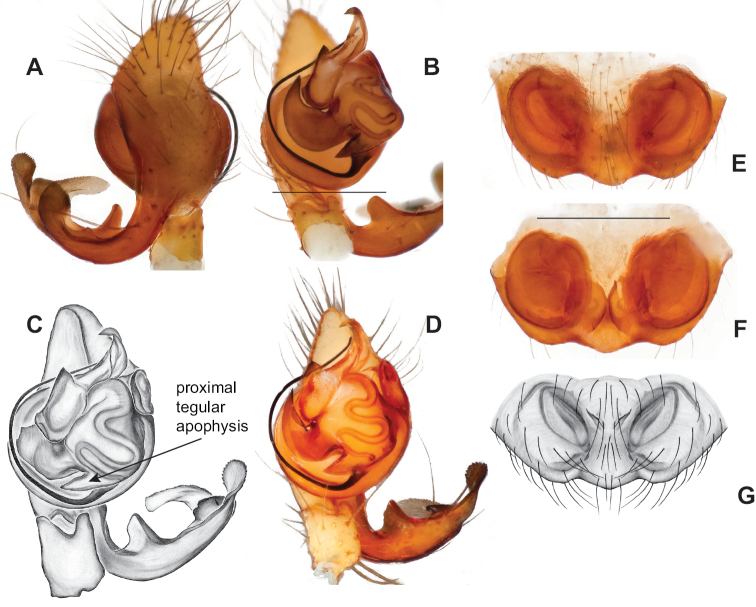
*Nesticusroanensis* sp. nov. genitalia. North Carolina, Mitchell Co., Roan Mountain, below Roan High Bluff, holotype male (SDSU_TAC000675) palp, dorsal (**A**), ventral (**B**) **C** North Carolina, Mitchell Co., upper Roan Valley, MCH 01_148, palp, ventral **D** North Carolina, Avery Co., Henson Creek at Henson Creek Baptist Church, MCH 07_138, palp, ventral. Epigynal variation. North Carolina, Mitchell Co., Roan Mountain, below Roan High Bluff, paratype ♀ (SDSU_TAC000675) epigynum, ventral (**E**), dorsal (**F**) **G** North Carolina, Mitchell Co., upper Roan Valley, MCH 01_148, ventral. Scale bar: 0.5 mm.

Male *Nesticusroanensis* possess a distinctive fork at the base of the tegulum unlike any other species in the species group (Fig. 28A–D). Like the sister species *N.paynei* the distal end of the paracymbial dorsal process is relatively rounded, vs. truncate. Females of *N.roanensis* are very similar to females of sister species *N.paynei*.

###### Description of ♂ holotype

**(SDSU_TAC000675).** Carapace dusky cream, faint dark pigment behind ocular area. Legs pale yellow / cream. Abdomen dirty pale cream with darker paired lateral pigmentation blotches. All eyes approximately equal in size, except for AMEs, ~ 1/4 width of ALEs. Eyes with rings of dark pigment. CL 1.6, CW 1.5, abdomen length 2.25, total body length 3.85. Leg I total length 11.35 (3.05, 0.75, 3.4, 2.95, 1.2), leg formula 1423, leg I / CW ratio 7.6. Paracymbium with a knob-shaped ventral process with a sclerotized retrolateral keel, a dorsal process with a rounded serrate, distal portion, a rectangular paradistal process, and a translucent, elongate, prolaterally-directed dorsal process. Median apophysis rectangular with an anteriorly directed edge coming to a point, translucent proximal spatulate edge lying above distal tegular process. Tegular process with arrowhead-like basal fork, distal process nearly as broad as long with apical point, nose-like bulge at the base of the distal process. Distal tip of conductor bent and directed prolaterally.

###### ♂ Variation.

Adult males from multiple collection events, including the lower elevation Henson Creek specimens, all closely approximate the holotype male. The distal portion of the tegular apophysis for the male from upper Roan Valley (MCH 01_148) is broken (Fig. 28C).

###### Description of ♀ paratype

**(SDSU_TAC000676).** Carapace dusky orange, conspicuous faint dark pigment behind ocular area. Legs pale orange. Abdomen dirty pale cream with darker paired lateral pigmentation blotches. All eyes approximately equal in size, except for AMEs, ~ 1/4 width of ALEs. Eyes with rings of dark pigment. CL 1.5, CW 1.4, abdomen length 2.35, total body length 3.85. Leg I total length 9.35 (2.65, 0.7, 2.65, 2.3, 1.05), leg formula 1423, leg I / CW ratio 6.7. Epigynum short, wider than long. Broad proximal median septum, narrowing slightly posteriorly. Lateral to proximal septum lie obliquely oriented, oval-shaped shallow pockets outlined by circular rings. Short, banana-shaped spermathecae visible lateral to distal septum, approximately perpendicular to septum. Viewed dorsally, circular internal lobes with interior margins bulging inwards and touching along the midline.

###### ♀ Variation.

The epigyna of females from multiple locations closely approximate the paratype female.

###### Distribution and natural history.

Restricted to Roan Mountain and immediate vicinity at elevations near or above 1800 meters, except for the Henson Creek location (~ 900 meters) on the southeastern flanks of Roan Mountain (Fig. 13). At high elevations spiders were found to be reasonably common under large stones in extensive north-facing talus habitat.

We have collected comprehensively in this region, finding the sister species *Nesticuspaynei* to the north and west, other *tennesseensis* group species to the east and southeast (Fig. 13), and other *Nesticus* further southwest. We believe that we have the small geographic distribution of *N.roanensis* well-circumscribed. The lower elevation Henson Creek sample location (~ 900 meters) has important conservation implications for this species, but more extensive regional sampling is needed to fully understand the distribution and abundance of this species.

###### Etymology.

Named after the highlands of Roan Mountain along the North Carolina / Tennessee border.

###### Remarks.

While male morphological evidence clearly supports this species as distinct in a “morphology first” framework (unique forked base of tegular apophysis), the UCE phylogenomic evidence is mixed. Concatenated likelihood supports the two sampled *Nesticusroanensis* populations as monophyletic (bootstrap = 100), but nested within a larger *N.paynei* clade (Fig. 3). However, this *N.paynei* paraphyly is weakly supported, with a bootstrap value of 59 and a sCF value of only 30.5. Collapsing this node results in a topology where *N.roanensis* shares a polytomous node with *N.paynei* populations (i.e., there is not strong support for *N.paynei* paraphyly). The ASTRAL topology more clearly favors reciprocal monophyly of *N.roanensis* and *N.paynei*, the former with a posterior probability of 0.99, the latter with a posterior probability of 1.0 (Fig. 4). This recovered monophyly, in combination with morphological diagnosability, would be consistent with our species criteria.

Mitochondrial data fail to support *Nesticusroanensis* as distinct from *N.paynei* (Fig. 6), with *N.roanensis* haplotypes intermixed with *N.paynei* haplotypes, and sometimes sharing nearly identical haplotypes. Because these taxa are closely parapatric it is possible that this reflects mitochondrial introgression at areas of contact on the northern slopes of Roan Mountain (Fig. 13). A combination of introgression and incomplete lineage sorting (or ILS alone) is also a possibility.

Overall, this taxonomic situation illustrates patterns of nuclear vs. mitochondrial vs. morphological discordance as also found elsewhere in Appalachian *Nesticus*. The male morphology of *N.roanensis* is as divergent as any taxon in the species group (Fig. 12C), female morphology and mitochondrial haplotypes are shared with *N.paynei*, while nuclear phylogenomic divergence is mixed. Rates of evolution in these different character classes appear to vary in this group of populations.

#### ﻿*nasicus* group, including:

*Nesticusnasicus* Coyle & McGarity, 1992

*Nesticusbrimleyi* Gertsch, 1984

*Nesticustempletoni* sp. nov.

*Nesticuscrosbyi* Gertsch, 1984

*Nesticusgertschi* Coyle & McGarity, 1992

*Nesticussecretus* Gertsch, 1984

*Nesticuscanei* sp. nov.

A species group strongly supported by nuclear phylogenomics (Figs 3, 4), with *Nesticusnasicus* sister to all other taxa in the group. This group is not recovered as monophyletic with mitochondrial data, with a monophyletic *N.nasicus* separate from a clade that includes remaining group members (Fig. 6). Four taxa, including *N.crosbyi*, *N.secretus*, *N.gertschi* and *N.canei* sp. nov. form a close-knit morphological and phylogenetic subgroup within the more inclusive species group. Mitochondrial relationships within this subgroup are discordant with nuclear and morphological evidence (see below).

**Figure 29.**
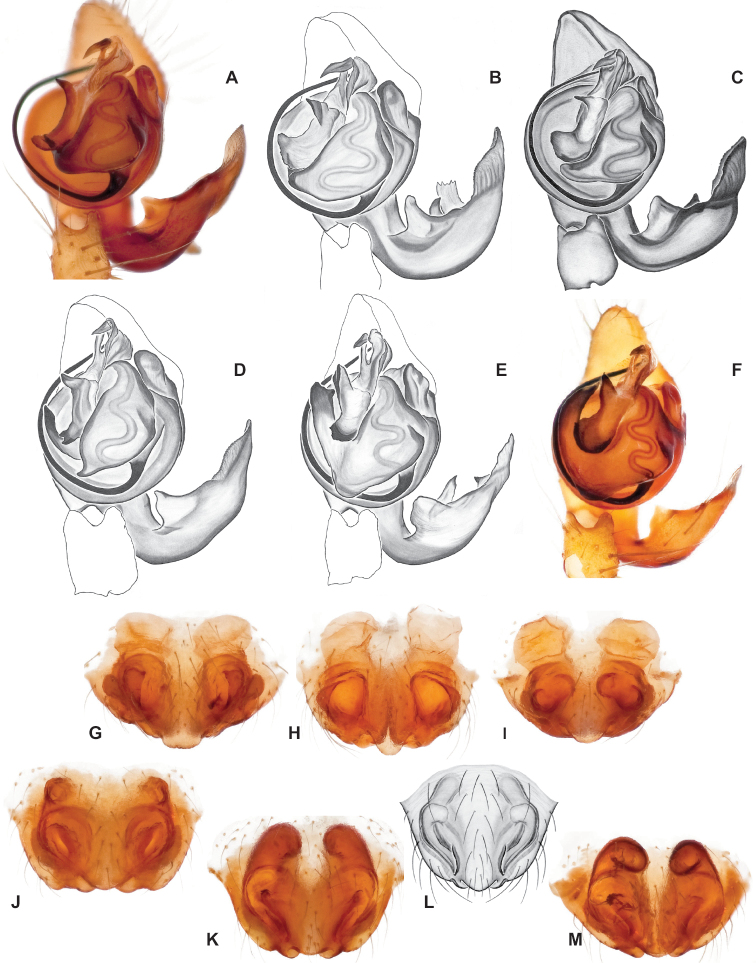
Comparative ♂♀ genitalia of *nasicus* group species; ♂ **A***Nesticusnasicus***B***N.brimleyi***C***N.templetoni***D***N.crosbyi***E***N.gertschi***F***N.canei*; ♀ **G***N.nasicus***H***N.brimleyi***I***N.templetoni***J***N.crosbyi***K***N.gertschi***L***N.secretus***M***N.canei*. All views ventral. See subsequent figures for specimen locations and voucher details.

Coyle and McGarity (1992) recognized a close morphological relationship between *Nesticusnasicus* and the previously described *N.brimleyi*, citing several shared male and female characters. They also commented on a possible relationship of these two species with *N.gertschi*, citing the “broad, translucent, spatulate, distal paracymbial process” as a possible defining feature for these three taxa (in a group they never formally named). Coyle and McGarity (1992) did not comment on the inclusion of previously described *N.secretus* Gertsch, 1984 or *N.crosbyi* in this group. We agree in recognizing the translucent, spatulate, distal paracymbial process as a defining feature for the entire species group (Fig. 29A–F). Also, the epigynum viewed ventrally is characterized by a protruding nose-like median septum bordered by prominent pockets (Fig. 29G–M).

**Figure 30.**
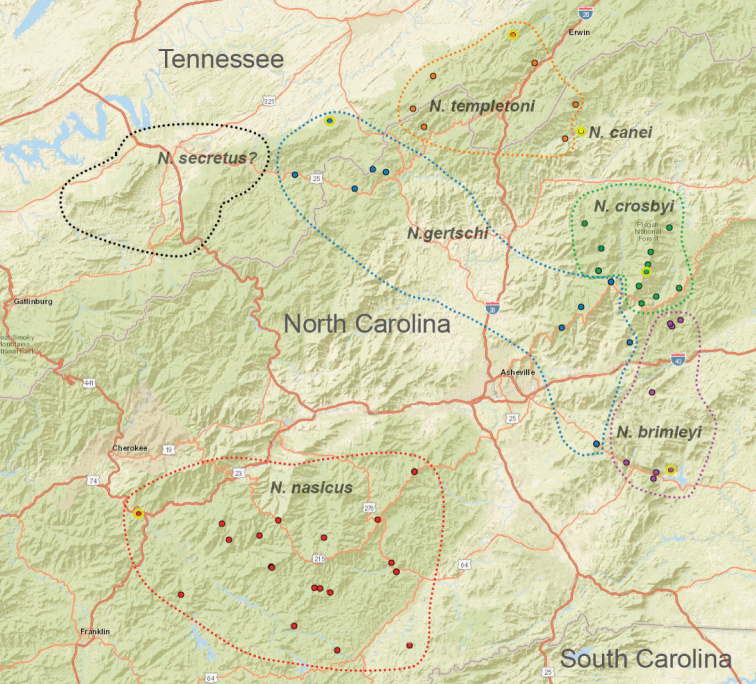
Distribution of *nasicus* group species. Type localities designated with yellow circles. Primary cities shown for geographic context. Dashed lines circumscribe known species distributions; possible distribution of *Nesticussecretus* is tentative, see text for details.

Species of the *nasicus* group are distributed in the montane southern Blue Ridge, both west (*N.nasicus*) and east (*N.brimleyi*, *N.templetoni*, *N.gertschi*, *N.canei*, *N.crosbyi*) of the Asheville Basin (Fig. 30). The geographic origin of *N.secretus*, perhaps not surprisingly, remains a secret. We hypothesize a possible geographic origin in the English or Green Mountains (see below and Fig. 30).

##### ﻿ Nesticus nasicus

Taxon classificationAnimaliaAraneaeNesticidae

Coyle & McGarity, 1992

5E146B38-C2B8-5BBB-A51E-B91AB2934E26

Figs 31A–D , 32A–H

Nesticus nasicus Coyle & McGarity, 1992: 162, figs 1–4, 7–14.

###### Material examined.

**Type material: *Holotype***: USA – **North Carolina, Jackson Co.** • ♂ holotype; 1 mile W of Dillsboro at Cowee Mountain Train Tunnel, rock bank; 28 Oct. 1990; T McGarity leg. AMNH; **New collections from type locality**: – **Jackson Co.** • ♂, 17♀; Cowee Mountain Train Tunnel, NW of Dillsboro; 35.3768°N, -83.268°W; 14 Aug. 1992; M. Hedin leg. **Non type material**: – **Buncombe Co.** • ♀; NE Mt. Pisgah, Hwy 151, head of McKinney Creek; 35.4448°N, -82.7225°W; 5 Sep. 2002; M. Hedin, M. Lowder, P. Paquin leg.; MCH 02_194; • ♀; NE Mt. Pisgah, Hwy 151, head of McKinney Creek; 35.4448°N, -82.7225°W; 22 Aug. 2004; M. Hedin, R. Keith, J. Starrett, S. Thomas leg.; MCH 04_074; – **Haywood Co.** • ♀; Blue Ridge Parkway, vicinity Richland Balsam; 35.3666°N, -82.9915°W; 4 Aug. 1992; F. Coyle leg.; • 4♂, 2♀, 7 imm; Hwy 215, along West Fork Pigeon River; 35.339°N, -82.9016°W; 4 Sep. 2002; M. Hedin, F. Coyle, P. Paquin leg.; MCH 02_190; • 2♂, 8♀, 5 imm; Hwy 276, N Pigeon Gap; 35.3677°N, -82.7958°W; 4 Sep. 2002; M. Hedin, M. Lowder, P. Paquin leg.; MCH 02_193; – **Jackson Co.** • 3♀, 1 imm; Coward Mountain near Jackie Spring Gap; 35.3352°N, -83.0897°W; 1 Sep. 2002; M. Hedin, P. Paquin leg.; MCH 02_178; • 4♀, 1 imm; Coward Mountain, E Wolfpen Gap; 35.3606°N, -83.1037°W; 1 Sep. 2002; M. Hedin, M. Lowder leg.; MCH 02_177; • 9♀; Mull Creek on Caney Fork Road, 11 mi. E Hwy 107; 35.3417°N, -83.0292°W; 11 Aug. 1992; M. Hedin leg.; • 2♀; Rich Mountain, SE Sugar Creek Gap; 35.2907°N, -83.004°W; 1 Sep. 2002; M. Hedin, P. Paquin leg.; MCH 02_179; • ♂; SW of Rich Mountain Bald, 0.5 mi E Sugar Creek Gap; 35.2915°N, -83.006°W; 26 Jun. 1992; B. Dellinger leg.; • ♀; SW of Rich Mountain Bald, 0.5 mi E Sugar Creek Gap; 35.2915°N, -83.006°W; 17 Apr. 1994; M. Hedin, B. Dellinger leg.; • 7♀; Wolf Creek at Cullowhee Creek, off Cullowhee Mountain Road; 35.2468°N, -83.1843°W; 11 Aug. 1992; M. Hedin leg.; – **Transylvania Co.** • ♀; along West Fork French Broad River, Silverstein Road, 2 mi. N Hwy 64; 35.1573°N, -82.8758°W; 19 Aug. 2007; M. Hedin, M. McCormack, S. Derkarabetian leg.; MCH 07_128; • ♀; below Connestee Falls, off Hwy 276 S Brevard; 35.1647°N, -82.7319°W; 2 Oct. 1992; B. Dellinger leg.; • ♂, 2♀; Hwy 215, 4.7 mi. NW Balsam Grove along Bald Knob branch; 35.2568°N, -82.9098°W; 13 Aug. 1992; M. Hedin leg.; • 5♀; Hwy 215, S Pinhook Gap; 35.2575°N, -82.9204°W; 22 Aug. 2004; M. Hedin, R. Keith, J. Starrett, S. Thomas leg.; MCH 04_073; • 2♀, 1 imm; Hwy 276 at Davidson River, opposite Stillwater Branch; 35.284°N, -82.7591°W; 28 Aug. 2001; M. Hedin, M. Lowder, P. Paquin leg.; MCH 01_180; • 4♀; Hwy 276 at Davidson River, opposite Stillwater Branch; 35.284°N, -82.7591°W; 4 Sep. 2002; M. Hedin, M. Lowder, P. Paquin leg.; MCH 02_192; • ♀; Hwy 276, Looking Glass Creek just N Looking Glass Falls; 35.2978°N, -82.7676°W; 19 Aug. 2007; M. McCormack, S. Derkarabetian leg.; MCH 07_129; • ♀; Hwy 281, E Owens Gap; 35.1957°N, -82.9608°W; 20 Aug. 2004; M. Hedin, R. Keith, J. Starrett, S. Thomas leg.; MCH 04_071; • ♀, 1 imm; N Fork French Broad, FR 140 off Hwy 215; 35.2503°N, -82.889°W; 4 Sep. 2002; M. Hedin, M. Lowder, P. Paquin leg.; MCH 02_189.

###### Diagnosis.

**Figure 31.**
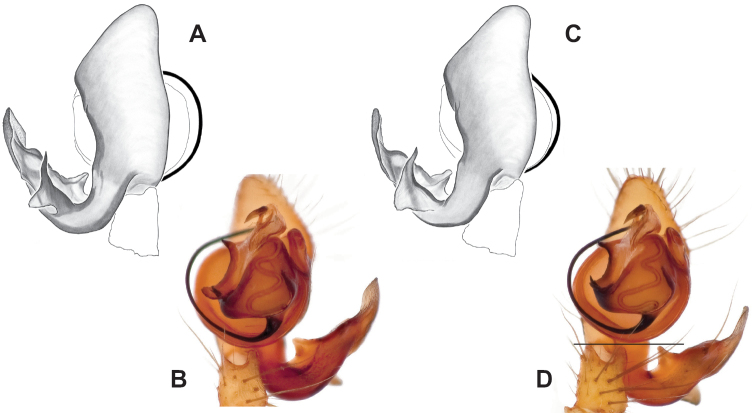
*Nesticusnasicus* ♂ palps. North Carolina, Transylvania Co., Hwy 215, NW of Balsam Grove, MCH specimen #1155, dorsal (**A**), ventral (**B**). North Carolina, Haywood Co., along West Fork Pigeon River, MCH 02_190, dorsal (**C**), ventral (**D**). Scale bar: 0.5 mm.

Males may be distinguished from other members of the species group by the palp with a uniquely shaped tegular apophysis (except in comparison to *Nesticusbrimleyi*), combined with a narrow-based paracymbial dorsal process (Fig. 31A, C) which differs greatly from *N.brimleyi*. The *N.nasicus* epigynum is very similar to *N.brimleyi* and *N.templetoni*. When viewed dorsally all possess “crinkled” sac-shaped structures above (anterior to) the main epigynal plate, which we hypothesize are homologous to vulval pockets (Vp) as seen in Japanese *Nesticus* (Suzuki and Ballarin 2020). Further diagnostic features for *N.nasicus* are discussed in Coyle and McGarity (1992).

###### Variation.

Notable variation exists in the shape of the dorsal process of the paracymbium, which is sometimes narrow and finger-like (Coyle and McGarity 1992: figs 1, 2), or fishtailed (Fig. 31A–D), with variation in the shape of the end of the process. Both the distomedial and dorsomedial paracymbial processes vary in presence across populations, with a distomedial process found in West Fork Pigeon River and Cowee Mountain males (Coyle and McGarity 1992: fig. 2), and a dorsomedial process found only in Cowee Mountain males; these processes are lacking in males from other populations. As discussed below similar population-level variation in these processes is observed in *Nesticustempletoni*. The shape of both the lateral and apical processes of the median apophysis also varies across populations (Fig. 31A–D).

**Figure 32.**
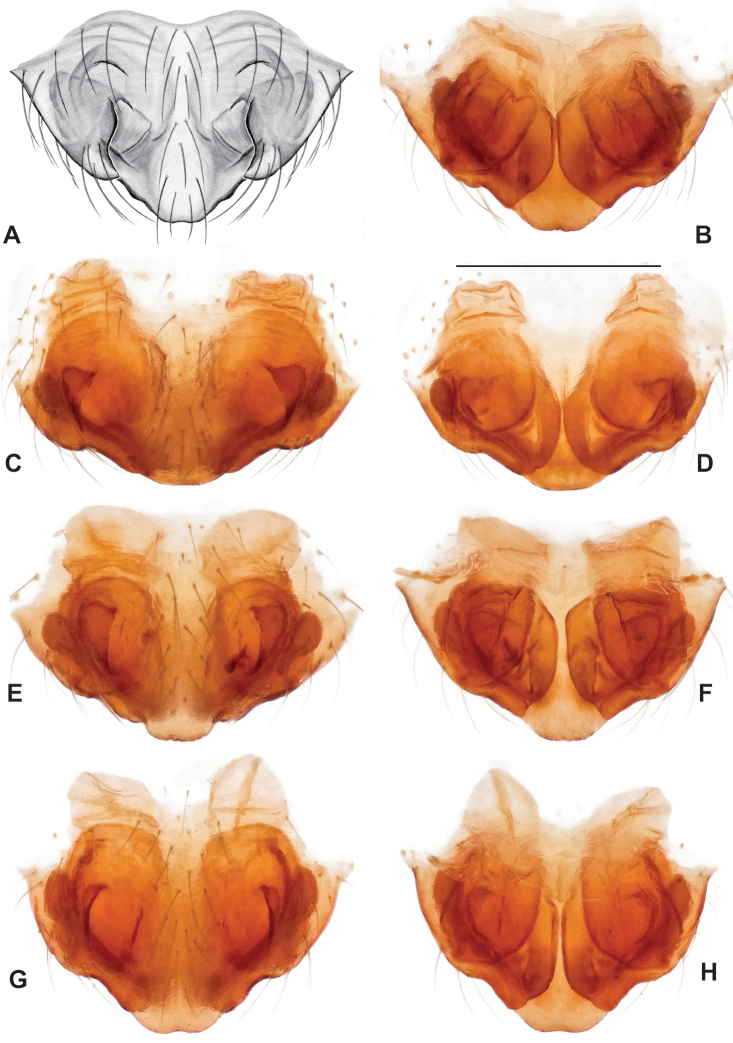
*Nesticusnasicus* epigynal variation. North Carolina, Haywood Co., along West Fork Pigeon River, MCH 02_190, ventral (**A**), dorsal (**B**). North Carolina, Jackson Co., Coward Mountain, E of Wolfpen Gap, MCH 02_177, ventral (**C**), dorsal (**D**). North Carolina, Transylvania Co., Hwy 276 at Davidson River, MCH 02_192, ventral (**E**), dorsal (**F**). North Carolina, Transylvania Co., Hwy 215, NW of Balsam Grove, MCH specimen #1157, ventral (**G**), dorsal (**H**). Scale bar: 0.5 mm.

Epigyna vary across sample locations in the length of the projection of the median septum, the shape of the epigynal pockets (though generally spherical), the width of epigynal pocket lateral hoods, and the length of the spermathecae (Fig. 32A–H).

###### Distribution and natural history.

Previously known only from two locations but now known to be reasonably widespread in the Great Balsam and Pisgah Mountains southwest of Asheville North Carolina, west of the Asheville Basin (Fig. 30). The southeastern Connestee Falls population, found east of the French Broad River, is a geographic outlier.

###### Remarks.

No obvious phylogeographic trends are apparent in the mitochondrial data, with geographically separate locations seemingly less genetically divergent than in other similarly widespread taxa (Fig. 6). This is particularly striking considering the notable male morphological variation observed across populations.

##### ﻿ Nesticus brimleyi

Taxon classificationAnimaliaAraneaeNesticidae

Gertsch, 1984

11B9B86F-C80A-555D-BD28-2E37B397C175

Figs 33A–C , 34A–H

Nesticus brimleyi Gertsch, 1984: 30, figs 126–128, 138–140; Coyle and McGarity 1992: figs 5, 6; Holler et al. 2020: 230.

###### Material examined.

**Type material: *Holotype***: USA – **North Carolina, Rutherford Co.** • ♂ holotype; Rumbling Bald Cave, Lake Lure, Rumbling Bald Mountain; 2 Jul. 1977; P. Hertl leg; AMNH; **New collections from near type locality.** – **Rutherford Co.** • 2♂, 6♀; SE side of Rumbling Bald Mountain, N of Lake Lure; 35.4487°N, -82.2167°W; 18 Aug. 1992; M. Hedin leg. **Non type material**: – **Henderson Co.** • ♀; Hwy 74 along Hickory Creek, E of Bearwallow; 35.4591°N, -82.3035°W; 20 Aug. 2007; M. Hedin, M. McCormack, S. Derkarabetian leg.; MCH 07_134; – **McDowell Co.** • ♀; headwaters of Crooked Creek, Mt. Hebron Road, N of Cross Mountain; 35.5726°N, -82.2532°W; 20 Aug. 2007; M. Hedin, M. McCormack, S. Derkarabetian leg.; MCH 07_135; • ♀; near Curtis Creek campground, FR 482, N of Old Fort; 35.6889°N, -82.1976°W; 20 Aug. 2007; M. Hedin, M. McCormack, S. Derkarabetian leg.; MCH 07_136; • 2♀; Newberry Creek above Horse branch, N of Old Fort; 35.6825°N, -82.217°W; 20 Aug. 2001; M. Hedin, M. Lowder, R. McClanahan leg.; MCH 01_141; • ♂, 11♀; Newberry Creek, N of Old Fort; 35.6789°N, -82.214°W; 22 Aug. 2004; M. Hedin, R. Keith J. Starrett, S. Thomas leg.; MCH 04_075; – **Rutherford Co.** • ♂, ♀; Chimney Rock Park, Moonshiner’s Cave; 5 May. 1999; M. Hedin, B. Dellinger leg.; MCH 99_014; • ♀; S side Round Top Mountain, just N of Chimney Rock; 35.4439°N, -82.2451°W; 5 May. 1999; M. Hedin, B. Dellinger leg.; MCH 99_015.

###### Diagnosis.

**Figure 33.**
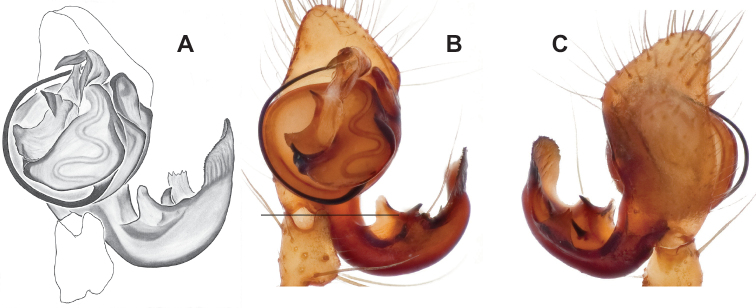
*Nesticusbrimleyi* ♂ palps **A** North Carolina, Rutherford Co., SE side of Rumbling Bald Mountain, MCH specimen #1247, ventral. North Carolina, Henderson Co., Newberry Creek, MCH 04_075, palp, ventral (**B**), dorsal (**C**). Scale bar: 0.5 mm.

**Figure 34.**
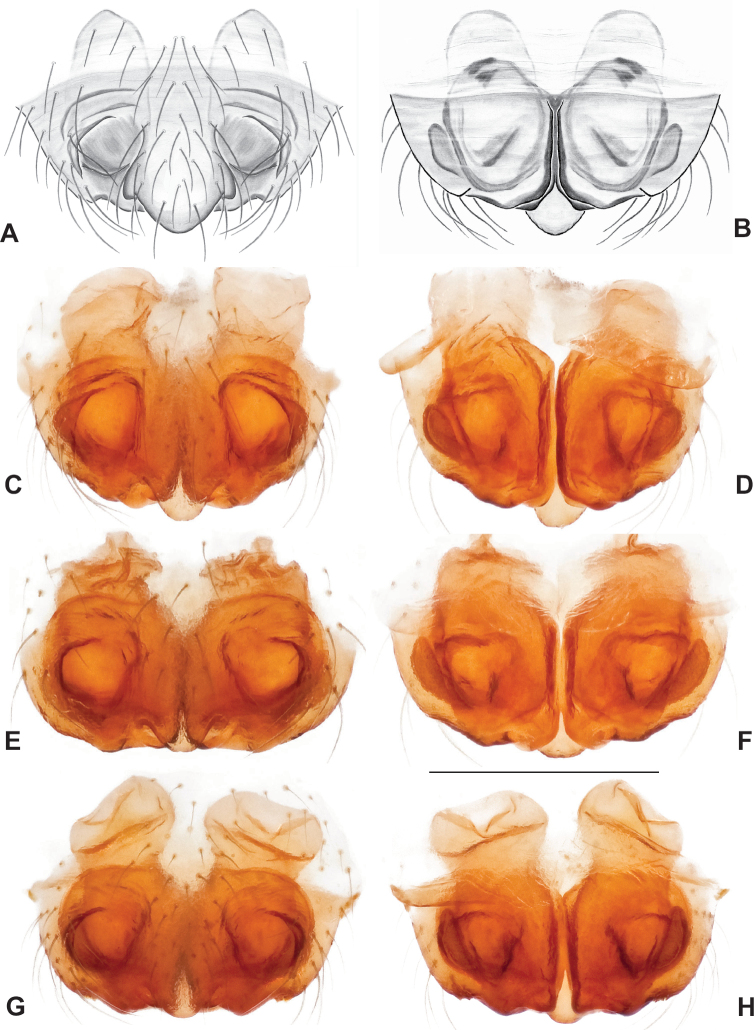
*Nesticusbrimleyi* epigynal variation. North Carolina, Rutherford Co., SE side of Rumbling Bald Mountain, MCH specimen #1254, ventral (**A**), dorsal (**B**). North Carolina, Henderson Co., W of Bat Cave, MCH 07_134, ventral (**C**), dorsal (**D**). North Carolina, McDowell Co., headwaters of Crooked Creek, MCH 07_135, ventral (**E**), dorsal (**F**). North Carolina, Henderson Co., Newberry Creek, MCH 04_075, ventral (**G**), dorsal (**H**). Scale bar: 0.5 mm.

Male paracymbium with three medial processes that lie between the ventral and dorsal processes, including ventromedial, distomedial, and dorsomedial processes (Fig. 2D.; see also Coyle and McGarity 1992: figs 4, 5). We have not seen populations of other species that simultaneously include all three processes. Also, a distally-thin tegular apophysis projects beneath the median apophysis (Fig. 33A–C). The epigynum is very similar to that of *Nesticustempletoni* (compare Fig. 34A–H to Fig. 36A–J).

###### Variation.

In the northern Newberry Creek population the male distomedial process is reduced (but present as low spikes), and the base of the dorsal paracymbial processes is wider then narrows to a forked tip (Fig. 33A–C). Epigynal variation is limited, even across northern vs. southern disjunct populations (Fig. 34A–H). Described by Gertsch as a “*pale cavernicole*”, but many populations are from boulderfield void spaces, and most specimens are not pale.

###### Distribution and natural history.

Previously known only from fissure caves, including those summarized by Holler et al. (2020) from Polk and Rutherford counties. Included here are many new records from near surface populations, including new northern records from Henderson and McDowell Counties (Fig. 30). For example, at Newberry Creek (MCH 04_075), spiders were “common in a ... well shaded hemlock/ rhododendron” boulderfield. The northwards distributional extension, and demonstration of an overall larger geographic and microhabitat distribution, has important conservation implications for this species.

###### Remarks.

*Nesticusbrimleyi* is strongly supported by nuclear phylogenomics as sister to *N.templetoni* but is geographically separated from this species by highlands occupied by other members of the species group (*N.gertschi*, *N.crosbyi*, and *N.canei*; Fig. 30). The mitochondrial gene tree includes two strongly supported geographic subclades (Fig. 6), corresponding to southern (Broad River drainage) versus northern *N.brimleyi* populations (Fig. 30).

##### ﻿ Nesticus templetoni sp. nov.

Taxon classificationAnimaliaAraneaeNesticidae

F5500973-72AC-55DC-BD44-7364473878B5

https://zoobank.org/5AB0E873-4666-4B6A-A213-72A3B24D978C

Figs 35A–H , 36A–J

###### Material examined.

**Type material: *Holotype***: USA – **Tennessee, Unicoi Co.** • ♂ holotype; Rich Mountain, Clarks Creek; 36.1457°N, -82.5278°W; 10 Aug. 2004; M. Hedin, R. Keith, J. Starrett, S. Thomas leg.; MCH 04_036 (SDSU_TAC000669); ***Paratypes*.**– **Tennessee, Unicoi Co.** • ♂, ♀; Rich Mountain, Clarks Creek; 36.1457°N, -82.5278°W; 10 Aug. 2004; M. Hedin, R. Keith, J. Starrett, S. Thomas leg.; MCH 04_036; **Non type material**: – **North Carolina, Madison Co.** • ♂, 2♀; East Prong Hickory Fork Creek, off Hwy 212; 35.999°N, -82.7033°W; 21 Aug. 2001; M. Hedin, M. Lowder leg.; MCH 01_144; – **North Carolina, Yancey Co.** • ♀; E Spivey Gap, Hwy 19W, along Big Creek, NW of Sioux; 36.0342°N, -82.4043°W; 21 Aug. 2001; M. Hedin, M. Lowder leg.; MCH 01_146; • 4♂, 2♀; Scronce Creek Road, W of Bee Log; 35.9805°N, -82.4245°W; 22 Oct. 2012; M. Hedin, J. Bond, F. Coyle leg.; MCH 12_141; – **Tennessee, Greene Co.** • 2♂, 6♀; Bald Mountain Road, NW Camp Creek Bald; 36.0284°N, -82.7253°W; 10 Aug. 2004; M. Hedin, R. Keith, J. Starrett, S. Thomas leg.; MCH 04_038; • 7♀, 10 imm; Bald Mountains, E Greystone Mountain, Round Knob Road; 36.0799°N, -82.6859°W; 10 Aug. 2004; M. Hedin, R. Keith, J. Starrett, S. Thomas leg.; MCH 04_037; – **Tennessee, Unicoi Co.** • ♂, 2♀; along Mill Creek, Mill Creek Road on Rich Mountain, NE of Ernestville; 36.1018°N, -82.4859°W; 22 Aug. 2007; M. Hedin, M. McCormack, S. Derkarabetian leg.; MCH 07_147; • 13♀, 3 imm; Rich Mountain, Clarks Creek; 36.1457°N, -82.5278°W; 10 Aug. 2004; M. Hedin, R. Keith, J. Starrett, S. Thomas leg.; MCH 04_036.

###### Diagnosis.

**Figure 35.**
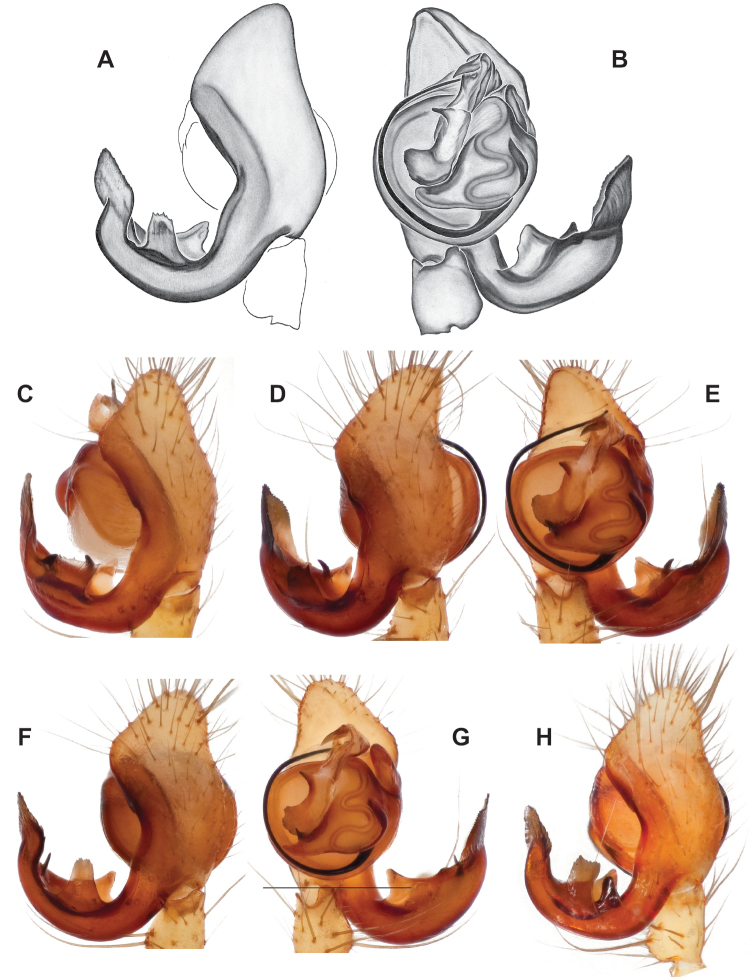
*Nesticustempletoni* sp. nov. ♂ palps. North Carolina, Unicoi Co., Rich Mountain, Clark Creek, MCH 04_036 (SDSU_TAC000669), dorsal (**A**), ventral (**B**) **C** Tennessee, Greene Co., Bald Mountain Road, MCH 04_038, dorsal. Tennessee, Unicoi Co., along Mill Creek, MCH 07_147, dorsal (**D**), ventral (**E**). North Carolina, Madison Co., East Prong Hickory Fork Creek, MCH 01_144, dorsal (**F**), ventral (**G**). North Carolina, Yancey Co., Scronce Creek Road, MCH 12_141, dorsal (**H**). Scale bar: 0.5 mm.

In comparison to its sister species *Nesticusbrimleyi* (see above), males of *N.templetoni* can be diagnosed by a shortened tegular apophysis (of variable shape) with a small, sclerotized extension lying behind the lateral process of the median apophysis, and never possessing all three medial paracymbial processes (Fig. 35A–H). The epigynum is very similar to that of *N.brimleyi*, with epigynal pockets in the latter generally more circular with stronger lateral hoods (Fig. 34A–H).

###### Description of ♂ holotype

**(SDSU_TAC000669).** Carapace cream-colored, very faint pigment in ocular area. Legs pale yellow to cream. Abdomen mostly pale cream, faint paired lateral pigmentation blotches. All eyes approximately equal in size, except for AMEs, ~ 1/4 width of ALEs. Eyes with rings of dark pigment. CL 1.25, CW 1.1, abdomen length 1.6, total body length 2.85. Leg I total length 9.85 (2.75, 0.5, 3, 2.6, 1), leg formula 1423, leg I / CW ratio 9.0. Palp with shoe-shaped tegular apophysis, with small dark sclerotized extension lying behind lateral process of median apophysis. Lateral process of median apophysis itself concave, broadening and well-sclerotized along edge, distal process drawn into thin tip. Ventral process of paracymbium translucent and triangular, distal process spatulate (consistent with species group), dorsal process wide at base, translucent, relatively short. Short, dark, conspicuous ventromedial process (Fig. 35A).

###### ♂ Variation.

Extensive population-level variation is seen in the male palps across relatively short geographic distances in this species. This includes variation in the shape of the shoe-shaped tegular apophysis and the sclerotized extension, the presence and shape of the paracymbial ventromedial and distomedial processes, and the shape of the dorsal paracymbial process (Fig. 35A–H). Mill Creek males approximate type males (Fig. 35D, E). Western Bald Mountain Road males possess a dorsal process that is nearly square in shape and includes a unique distal spike, with both ventromedial and distomedial paracymbial processes (Fig. 35C). Northwestern Hickory Fork Creek males only possess a distomedial paracymbial process (Fig. 35F, G). Southern Scronce Creek males lack ventro- and distomedial processes altogether and possess a dorsal process that is particularly wide at the base with a unique basal sclerotized extension (Fig. 35H), perhaps representing a dorsomedial process that has migrated to the edge of the paracymbium.

###### Description of ♀ paratype

**(SDSU_TAC000670).** Carapace color as in male. Legs pale yellow to cream. Abdomen with paired, lateral darker markings on dirty gray background. Eye development as in male, eyes with rings of dark pigment. CL 1.3, CW 1.25, abdomen length 1.8, total body length 3.1. Leg I total length 10.75 (3, 0.75, 3.1, 2.7, 1.2), leg formula 1423, leg I / CW ratio 8.6. Epigynum, viewed laterally, with a prominent nose-like cream-colored median septum, like other members of the species group. Viewed ventrally, oval-shaped epigynal pockets lateral to median septum, angled outwards from top to bottom. Dorsal view showing spermathecae below epigynal pockets, angled upwards obliquely, approximately avocado-shaped. With sac-shaped structures anterior to epigynal pockets, hypothesized as vulval pockets (Vp). Epigynal plates meeting along midline, parallel from top to bottom.

###### ♀ Variation.

**Figure 36.**
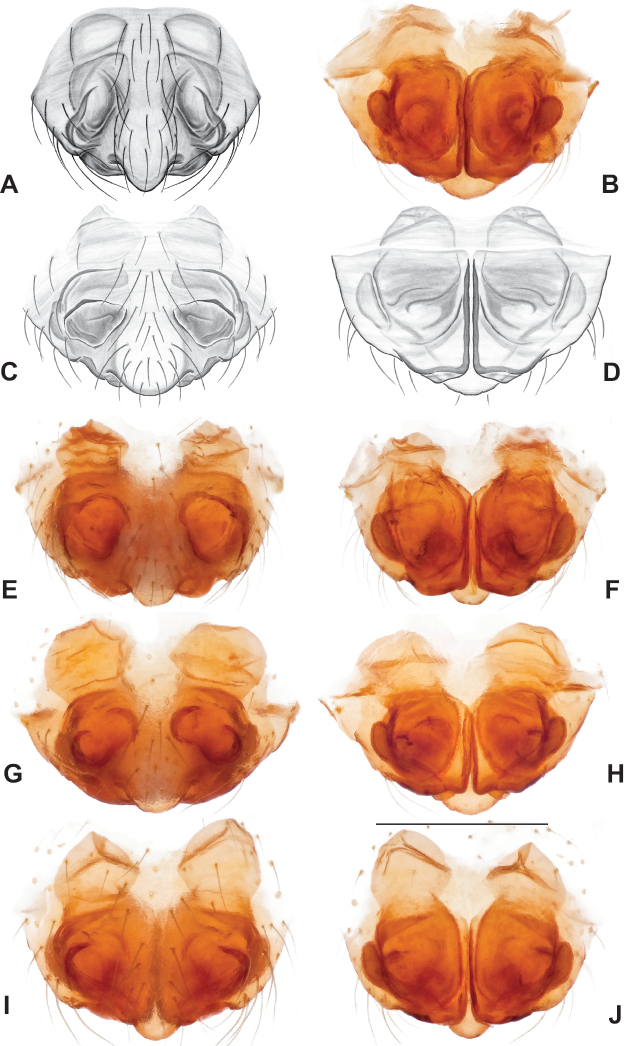
*Nesticustempletoni* sp. nov. epigynal variation. North Carolina, Unicoi Co., Rich Mountain, Clark Creek, MCH 04_036 (SDSU_TAC000670), ventral (**A**), dorsal (**B**). North Carolina, Madison Co., East Prong Hickory Fork Creek, MCH 01_144, ventral (**C**), dorsal (**D**). Tennessee, Greene Co., Bald Mountains, MCH 04_037, ventral (**E**), dorsal (**F**). Tennessee, Unicoi Co., along Mill Creek, MCH 07_147, ventral (**G**), dorsal (**H**). North Carolina, Yancey Co., E of Spivey Gap, MCH 01_146, ventral (**I**), dorsal (**J**). Scale bar: 0.5 mm.

Variation exists in the shape of the lateral epigynal pockets (ventral view), but the overall vulval pocket morphology, spermathecal shape, and parallel epigynal plates is fairly conserved across populations (Fig. 36A–J).

###### Distribution and natural history.

Populations have been collected from the larger Bald Mountains area along the North Carolina / Tennessee border, southwest of Erwin, Tennessee (Fig. 30). Most populations have been collected from boulderfield void spaces. For example, at the type locality, spiders were found to be “common” in a “deep, moist boulderfield”. At Bald Mountain Road (MCH 04_038) spiders were found near and under rock accumulations adjacent to a small stream.

###### Etymology.

This species is named to recognize and honor Dr. Alan Templeton, Charles Rebstock Professor Emeritus of Biology, Washington University. A brilliant evolutionary, speciation, and conservation biologist, with a deep love for all biodiversity. PhD dissertation advisor of MH, honored here for his inspiration and support during the first author’s formative years as an evolutionary biologist.

###### Remarks.

Two strongly supported geographic subclades are recovered with mitochondrial data (Fig. 6), corresponding to eastern / southern (Clarks Creek, Mill Creek, Scronce Creek) versus western (Bald, Bald Mtn Road, Hickory Fork) sample locations (Fig. 30). Increased nuclear phylogenomic sampling might ultimately reveal these geographic populations as reciprocally monophyletic.

##### ﻿ Nesticus crosbyi

Taxon classificationAnimaliaAraneaeNesticidae

Gertsch, 1984

787A7E46-5C9B-55CA-AAA9-E920E100F637

Figs 37A–D , 38A–H

Nesticus crosbyi Gertsch, 1984: 33, figs 173, 174.

###### Material examined.

**Type material: *Holotype***: USA – **North Carolina, Yancey Co.** • ♀ holotype; Commissary Ridge Trail, 100 yards west of main peak of Mt. Mitchell; 22 Aug. 1960; T.C. Barr leg.; AMNH; **New collections from near type locality**: – **Yancey Co.** • 2♂, 5♀; Mt. Mitchell SP, just NE summit parking lot; 35.7671°N, -82.2641°W; 15 Aug. 1992; M. Hedin leg.; • ♂, 2♀; Mt. Mitchell SP, just NE summit parking lot; 35.7671°N, -82.2641°W; 4 May. 1999; M. Hedin, B. Dellinger leg.; MCH 99_012; • 2♀; Mt. Mitchell SP, just NE summit parking lot; 35.7671°N, -82.2641°W; 25 Aug. 2005; M. Hedin, R. Keith, J. Starrett, S. Thomas leg.; MCH 05_083; **Non type material**: – **Buncombe Co.** • 2♀, 3 imm; Walker branch of Dillingham Creek, drainage N of Walker Falls branch, Little Andy Creek; 35.7677°N, -82.3594°W; 25 Aug. 2001; M. Hedin, M. Lowder, P. Paquin leg.; MCH 01_168; • ♂, 2♀; Walker branch of Dillingham Creek, drainage N of Walker Falls branch, Little Andy Creek; 35.7677°N, -82.3594°W; 5 Sep. 2002; M. Hedin, M. Lowder, P. Paquin leg.; MCH 02_196; • ♀, 1 imm; SW of Cane River Gap, Hwy 197, 5 mi ENE Barnardsville; 35.8036°N, -82.3536°W; 25 Aug. 2001; M. Hedin, M. Lowder, P. Paquin leg.; MCH 01_167; – **Yancey Co.** • ♂, ♀; Black Mountains, near Cattail Peak; 35.7977°N, -82.2564°W; 4 May. 1999; M. Hedin, B. Dellinger leg.; MCH 99_012a; • 1 imm (identification based on geography and mitochondrial evidence); Blue Ridge Parkway at Bald Knob Ridge Trail, near entrance to Mt. Mitchell SP; 35.715°N, -82.2736°W; 21 Aug. 2007; M. Hedin, M. McCormack, S. Derkarabetian leg.; MCH 07_141; • 2♂, 2♀; FR 472 along South Toe River, below Chestnut knob; 35.7265°N, -82.2452°W; 20 Aug. 2001; M. Hedin, M. Lowder, R. McClanahan leg.; MCH 01_143; • 2♀; Mt. Mitchell SP, near Mt Craig; 35.7776°N, -82.2616°W; 4 May. 1999; M. Hedin, B. Dellinger leg.; MCH 99_012a; • ♀, 1 imm; Mt. Mitchell SP, off Hwy 128, between Mt Gibbes and Stepps Gap; 35.7432°N, -82.2788°W; 26 Aug. 2001; M. Hedin, M. Lowder, P. Paquin leg.; MCH 01_170; • 1 imm (identification based on UCE and mitochondrial evidence); Shuford Creek, off Whiteoak Rd., SW of Celo; 35.8382°N, -82.2193°W; 21 Aug. 2007; M. Hedin, M. McCormack, S. Derkarabetian leg.; MCH 07_139; • 2♀; south of Big Laurel Mountain, N off Blue Ridge Parkway; 35.7401°N, -82.1991°W; 20 Aug. 2001; M. Hedin, M. Lowder, R. McClanahan leg.; MCH 01_142; • ♂, 2♀, 1 imm; Prices Creek Road at Price Creek; 35.8448°N, -82.3869°W; 22 Aug. 2007; M. Hedin, M. McCormack, S. Derkarabetian leg.; MCH 07_149.

###### Diagnosis.

**Figure 37.**
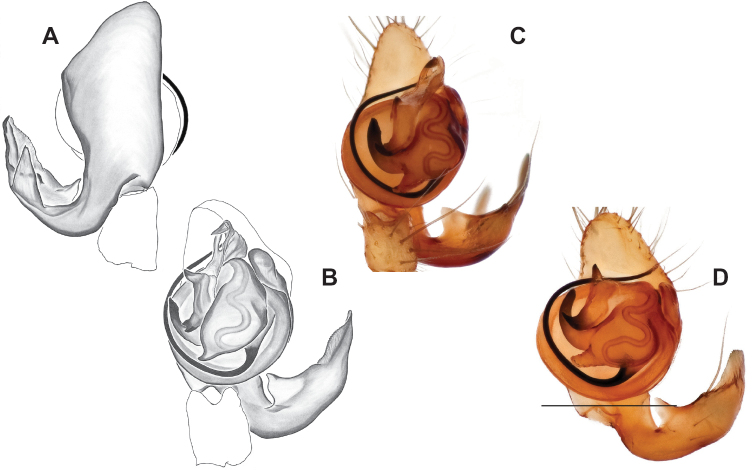
*Nesticuscrosbyi* ♂ palps. North Carolina, Yancey Co., Mt. Mitchell SP, just NE of summit parking lot, MCH specimen #1201, dorsal (**A**), ventral (**B**) **C** North Carolina, Buncombe Co., Prices Creek Road at Price Creek, MCH 07_149, ventral **D** North Carolina, Buncombe Co., Walker branch of Dillingham Creek, MCH 02_196, ventral. Scale bar: 0.5 mm.

**Figure 38.**
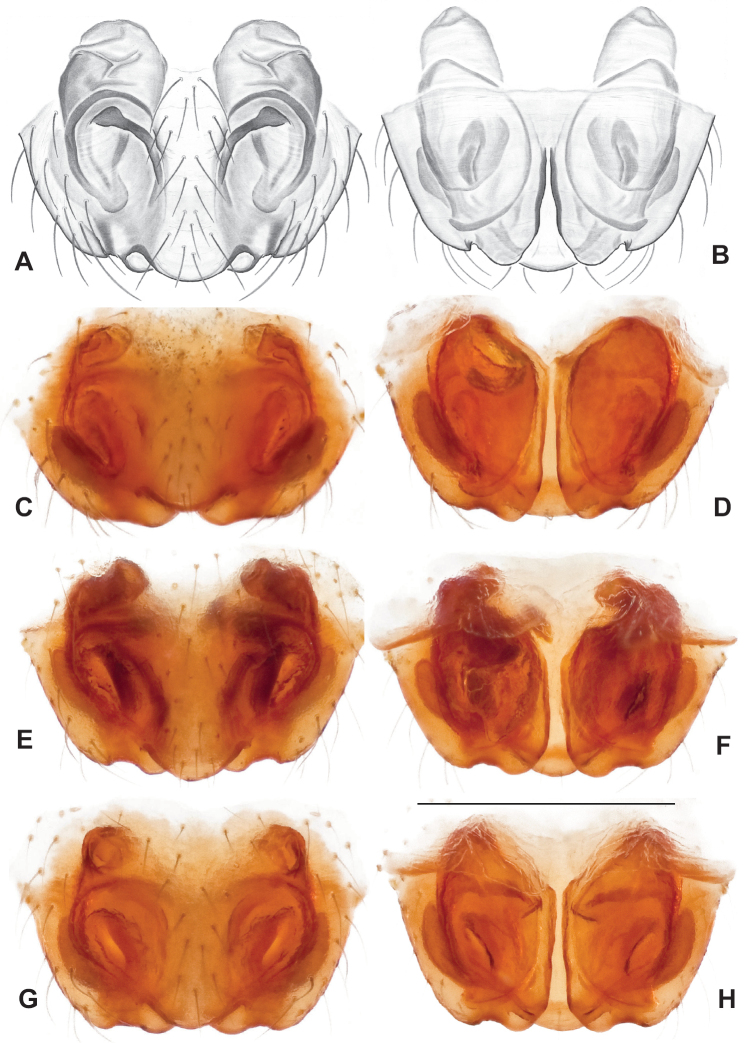
*Nesticuscrosbyi* epigynal variation. North Carolina, Yancey Co., Mt. Mitchell SP, MCH specimen #1204, ventral (**A**), dorsal (**B**). North Carolina, Buncombe Co., SW of Cane River Gap, MCH 01_167, ventral (**C**), dorsal (**D**). North Carolina, Buncombe Co., Prices Creek Road at Price Creek, MCH 07_149, ventral (**E**), dorsal (**F**). North Carolina, Buncombe Co., Walker branch of Dillingham Creek, MCH 02_196, ventral (**G**), dorsal (**H**). Scale bar: 0.5 mm.

Male palps differ in many ways from other members of the species group (including closest relatives), with a forked base of the tegulum, a narrow, curved tegular apophysis, a beak-like basal process of the median apophysis, and a translucent dorsal paracymbial process with a relatively wide base (Fig. 37A–D). Females have genitalia similar to members of the close-knit morphological and phylogenetic subgroup, also including *Nesticusgertschi*, *N.secretus*, and *N.canei*, but can be diagnosed by epigynal internal anterior plates/lobes that differ in shape (Fig. 38A–H) and appear to lack the hypothesized vulval pockets (Vp) seen in other members of the species group (Fig. 29G–M).

###### Description of ♂ from near type locality

**(MCH specimen #1201).** Carapace dusky cream to orange, conspicuous faint dark pigment behind ocular area and along carapace margins bleeding inwards. Legs pale yellow to cream. Abdomen mostly pale cream with crisp paired lateral pigmentation blotches. All eyes approximately equal in size, except for AMEs, ~ 1/4 width of ALEs. Eyes with rings of dark pigment. CL 1.6, CW 1.45, abdomen length 2.15, total body length 3.75. Leg I total length 9.85 (2.65, 0.65, 2.95, 2.5, 1.1), Leg formula 1423, leg I / CW ratio 6.8. Palp with forked base of the tegulum, including a short basal branch and a narrow, curved, dark, thin tegular apophysis. Median apophysis with lateral process well-sclerotized and beak-like, thin apical process. Paracymbium with well-developed triangular translucent ventral process, distal process typical for the species group, paradistal process reduced to a sclerotized low ridge, and a translucent dorsal process with a relatively wide base, mostly lacking distal serrations.

###### Variation.

Male variation was observed in the shape of the median apophysis lateral process, the paracymbial ventral process, and the proximal fork of the tegulum (Fig. 37A–D). Female variation was observed in the shape of the epigynal internal anterior lobes (Fig. 38A–H).

###### Distribution and natural history.

Previously known only from the type location (Mt. Mitchell), corresponding to the highest uplands east of the Mississippi River in North America, above 2000 meters in elevation. Our new records indicate that this species is more widespread in the Black Mountains (both to the north and southeast), and we include here new records from west of the Blacks, in the Great Craggy Mountains (Fig. 30). This demonstration of an overall larger geographic distribution, with populations also at lower elevations (e.g., Prices Creek at 930 m), has important conservation implications for this species.

Most collections have resulted in a relatively modest number of specimens taken. For example, at an apparently pristine boulderfield along the South Toe River (MCH 01_143), three persons each searching for 30 minutes collected four total adult specimens.

###### Remarks.

Strongly supported as a clade by UCE data (Figs 3, 4), but not recovered as monophyletic on the mitochondrial gene tree (Fig. 6), where sequences are intermixed with sequences from close relatives *Nesticusgertschi* and *N.canei*. Mitochondrial introgression and/or incomplete lineage sorting could explain this result, as all three species occur in the same geographic region (Fig. 30), making lineage contact and introgression possible.

##### ﻿ Nesticus gertschi

Taxon classificationAnimaliaAraneaeNesticidae

Coyle & McGarity, 1992

A9CF0E7E-BFAE-5D99-B568-C61E1961C363

Fig. 39A–F

Nesticus gertschi Coyle & McGarity, 1992: figs 15–20.

###### Material examined.

**Type material: *Holotype***: USA – **Tennessee, Greene Co.** • ♂ holotype; Cedar Creek Cave, 100 m into cave; 16 Mar. 1991; T. McGarity leg; AMNH; **New collections from type locality**: – **Tennessee, Greene Co.** • 2♂, 9♀; Cedar Creek Cave, 1 mi. S Cedar Creek; 21 Sep. 1992; M. Hedin, S. O’Kane leg. **Non type material**: – **North Carolina, Buncombe Co.** • ♂; 0.1 mi. NW Hickory Nut Gap Hwy 74, NW of Gerton; 35.4898°N, -82.3627°W; 5 May. 1999; M. Hedin, B. Dellinger leg.; MCH 99_013; • ♂, ♀, 1 imm; 0.1 mi. NW Hickory Nut Gap Hwy 74, NW of Gerton; 35.4898°N, -82.3627°W; 27 Aug. 2001; M. Hedin, M. Lowder, P. Paquin leg.; MCH 01_173; • 4♂, 7♀; Blue Ridge Parkway, Mile 370, 3 mi. SW Craggy Gardens turnoff; 35.6768°N, -82.4322°W; 15 Aug. 1992; M. Hedin leg.; • 2♂, 8♀; Flat Creek at NE edge of Montreat; 35.6528°N, -82.2972°W; 12 Aug. 1992; M. Hedin leg.; • 6♀; FR 63 along Mineral Creek, S of Dillingham; 35.7093°N, -82.3939°W; 21 Aug. 2007; M. Hedin, M. McCormack, S. Derkarabetian leg.; MCH 07_143; – **North Carolina, Madison Co.** • ♂, ♀; Anthodite Cave; 5 Jan. 2002; J.D. Mayes leg.; • ♂, 4♀; FR 467 to Rich Mountain, 0.5 mi. to jnct w/ Hwy 25/70, W of Hurricane; 35.9274°N, -82.7792°W; 22 Aug. 2007; M. Hedin, M. McCormack, S. Derkarabetian leg.; MCH 07_144; • 4♀, 1 imm; Rich Mountain, 0.5 mi. N Rich Mountain lookout; 35.9313°N, -82.806°W; 19 Aug. 2001; M. Hedin, M. Lowder leg.; MCH 01_140; – **North Carolina, Yancey Co.** • 3♀, 2 imm; Blue Ridge Parkway at Balsam Gap, just down Big Butt trail; 35.7495°N, -82.3343°W; 26 Aug. 2001; M. Hedin, M. Lowder, P. Paquin leg.; MCH 01_169; • 2♂, 3♀; Blue Ridge Parkway at Balsam Gap, just down Big Butt trail; 35.7495°N, -82.3343°W; 5 Sep. 2002; M. Hedin, M. Lowder, P. Paquin leg.; MCH 02_195; – **Tennessee, Cocke Co.** • 5♀; along French Broad River, north of Wolf Creek Bridge, FR 209; 35.9228°N, -82.9585°W; 12 Aug. 2004; M. Hedin, R. Keith, J. Starrett, S. Thomas leg.; MCH 04_044.

###### Diagnosis.

**Figure 39.**
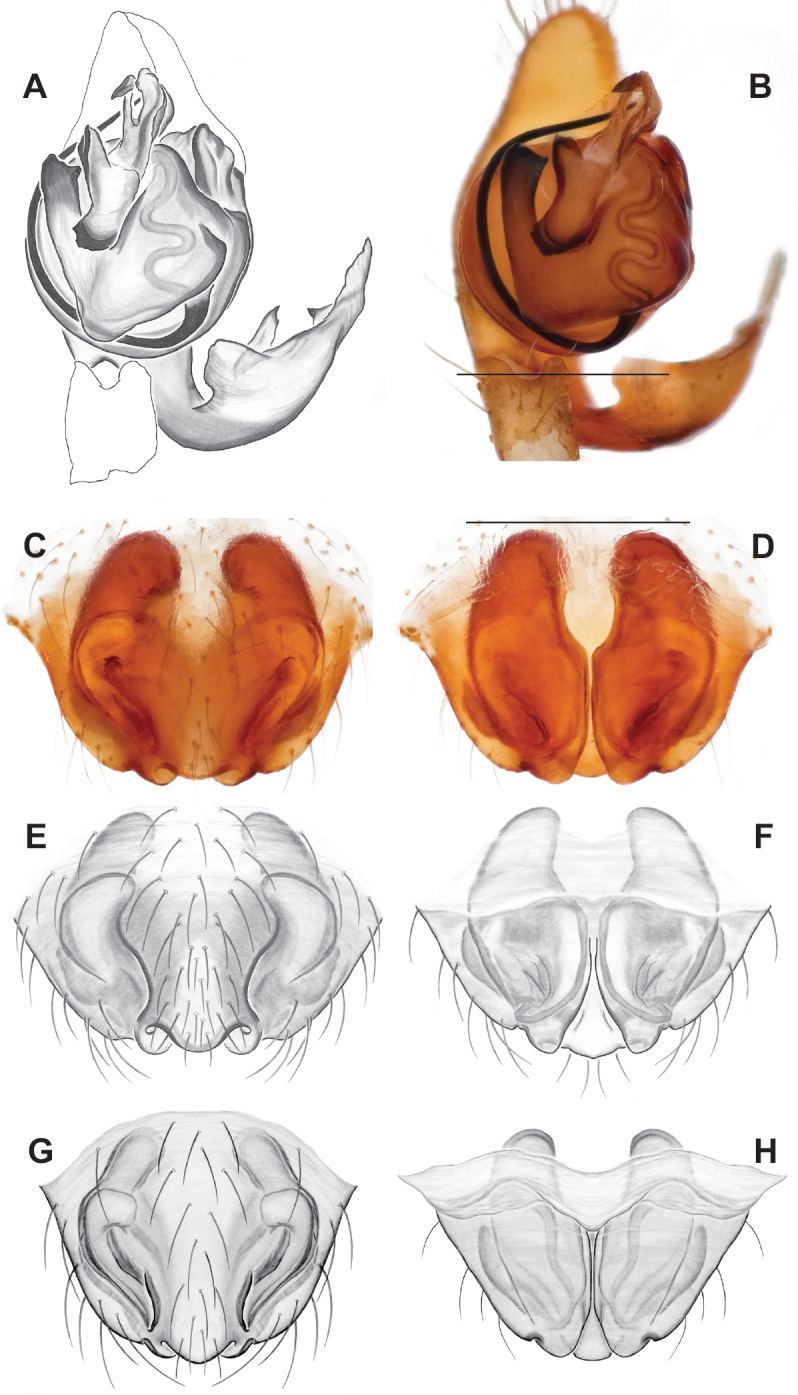
*Nesticusgertschi* and *N.secretus* genitalia. *N.gertschi***A** North Carolina, Yancey Co., Blue Ridge Parkway at Balsam Gap, MCH 02_195, ♂ palp, ventral **B** North Carolina, Buncombe Co., NW of Hickory Nut Gap, Hwy 74, MCH 01_173, ♂ palp, ventral. North Carolina, Buncombe Co., NW of Hickory Nut Gap, Hwy 74, MCH 01_173, epigynum, ventral (**C**), dorsal (**D**). North Carolina, Yancey Co., Blue Ridge Parkway at Balsam Gap, MCH 02_195, epigynum, ventral (**E**), dorsal (**F**). Scale bar: 0.5 mm. *N.secretus*Gertsch 1984 epigynum. Tennessee, Great Smoky Mountains National Park, ♀ holotype, ventral (**G**), dorsal (**H**).

See Coyle and McGarity (1992) for diagnosis comparing *Nesticusgertschi* to other members of the species group; here revised to recognize the close relationship to *N.canei* sp. nov. Males can be distinguished from the latter by the tegular apophysis tip (beyond bend) broad and truncate (Fig. 39A, B), and paracymbial distal process with subdistal processes. Females are very similar to *N.secretus* and *N.canei* sp. nov., with internal anterior plates of epigyna not projecting inwards and ventrally as strongly as in the latter species (Fig. 39C–F).

###### Variation.

This species shows surprisingly little genitalic variation despite a relatively large geographic distribution (e.g., compare ♂ Fig. 39A, B. to Coyle and McGarity (1992) figs 15–17), and obvious lowland geographic barriers (Fig. 30). Specimens from surface-dwelling populations are generally smaller in body size than cave-dwelling specimens from the type locality. Two female specimens (of five total) from surface collections along the French Broad River (MCH 04_044) lack eye pigmentation, while cave-dwelling specimens from both Cedar Creek Cave and Anthodite Cave possess eye pigmentation.

###### Distribution and natural history.

Previously known only from the type locality (Cedar Creek Cave), this species is a fairly widespread surface-dwelling species (Fig. 30). The geographic distribution is apparently fragmented with northern, central, and southern populations, with all but one known population from east of the French Broad River (Anthodite Cave being the exception).

Surface collections are mostly from shaded boulderfields, with field notes suggesting spiders to be “fairly common” under rocks in void spaces. Montreat specimens were found in dark cracks and crevices of a man-made rock wall within 3 meters of a stream.

###### Remarks.

As discussed below, possibly synonymous with *Nesticussecretus*.

Strongly supported as a clade by UCE data, with nuclear subclades corresponding to northern vs. central + southern collection locations (Figs 3, 4), separated by the Mars Hill lowland gap. Not recovered as monophyletic on the mitochondrial gene tree (Fig. 6), where sequences are intermixed with those of closely related *Nesticuscrosbyi* and *N.canei* sp. nov.

Gertsch (1984, p. 30) cites a record for *Nesticusreclusus* as (“*McDowell County, Montreat, 16 October, 1923, female*”). However, our 1992 collections from Montreat (now in Buncombe County) only include *N.gertschi*, which is the locally prevalent species (Fig. 30). Also, members of the *reclusus* group are not known from east of the Asheville Basin (Fig. 53). We have not seen the 1923 specimen but suspect either mislabeling or misidentification.

##### ﻿ Nesticus secretus

Taxon classificationAnimaliaAraneaeNesticidae

Gertsch, 1984

77D1FFC1-C1C7-59EF-AB01-5D8F80299961

Fig. 39G, H

Nesticus secretus Gertsch, 1984: 33, figs 173, 174.

###### Material examined.

**Type material: *Holotype***: USA – **Tennessee** • ♀ holotype; Great Smoky Mountains National Park; 8 Jul. 1933; W.J. Gertsch leg.; AMNH.

###### Remarks.

Gertsch cites the type data for this “*small, dusky epigean species with short legs*” as “female holotype from Little Pigeon River, Great Smoky Mountains National Park, Sevier County, Tennessee, 8 July 1933 (W.J. Gertsch)”. However, the label associated with the holotype female (see above) includes neither specific locality nor county information.

The type female is clearly a representative of the *nasicus* group, and is potentially synonymous with *Nesticusgertschi* (see Fig. 39C, D). However, essentially all eastern *nasicus* group populations are known from east of the French Broad River, while the Great Smoky Mountains National Park lies west of this (Fig. 30). Also, extensive collections from the eastern portion of the Great Smoky Mountains National Park have only ever resulted in the collection of members of the *tennesseensis* group (*N.cherokeensis* and *N.silvanus*, Fig. 13), or members of the *reclusus* group (*N.binfordae* and *N.reclusus*, Fig. 53). Both *N.binfordae* and *N.reclusus* have been collected from along the Little Pigeon River.

A possible region to search for *Nesticussecretus* would be the English or Green Mountains, west of the French Broad River, but not too distant from records for *N.gertschi* (Fig. 30). Because of this possibility we retain *N.secretus* as a valid taxon, pending further focused collection efforts.

##### ﻿ Nesticus canei sp. nov.

Taxon classificationAnimaliaAraneaeNesticidae

83A165F8-C232-53EC-9984-BFEC791675FE

https://zoobank.org/F17F97DD-6F1D-4A48-B9B7-122075D9EAE6

Fig. 40A–D

###### Material examined.

**Type material: *Holotype***: USA – **North Carolina, Yancey Co.** • holotype ♂; Hwy 19W along Cane River, near Egypt-Ramseytown Fire Station, near Lewisburg; 35.9921°N, -82.3927°W; 11 Aug. 2004; M. Hedin, R. Keith, J. Starrett, S. Thomas leg.; MCH 04_043 (SDSU_TAC000671); ***Paratypes***: – **Yancey Co.** • ♂, ♀; Hwy 19W along Cane River, near Egypt-Ramseytown Fire Station, near Lewisburg; 35.9921°N, -82.3927°W; 11 Aug. 2004; M. Hedin, R. Keith, J. Starrett, S. Thomas leg.; MCH 04_043; **Non type material**: – **Yancey Co.** • 9♀, 17 imm; Hwy 19W along Cane River, near Egypt-Ramseytown Fire Station, near Lewisburg; 35.9921°N, -82.3927°W; 11 Aug. 2004; M. Hedin, R. Keith, J. Starrett, S. Thomas leg.; MCH 04_043.

###### Diagnosis.

**Figure 40.**
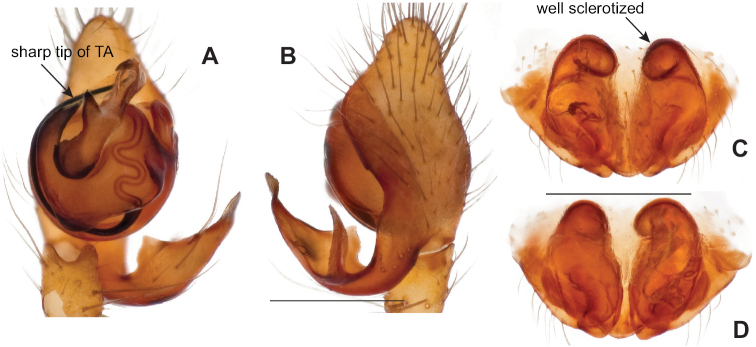
*Nesticuscanei* sp. nov. genitalia. North Carolina, Yancey Co., Hwy 19W, along Cane River, near Egypt–Ramseytown Fire Station, MCH 04_043 (SDSU_TAC000671), ♂ palp, ventral (**A**), dorsal (**B**). North Carolina, Yancey Co., Hwy 19W, along Cane River, near Egypt–Ramseytown Fire Station, MCH 04_043 (SDSU_TAC000672), epigynum, ventral (**C**), dorsal (**D**). Scale bar: 0.5 mm.

The male palp is like that of *Nesticusgertschi* (Fig. 39A, B) but the distal end of the tegular apophysis is acute rather than blunt, and the basal portion of median apophysis is relatively more expanded (Fig. 40A, B). The distal paracymbial process lacks the subdistal processes as found in *N.gertschi*. Female with dorsal portion of internal anterior lobes/plates well sclerotized, rounded anteriorly and curving ventrally (Fig. 40C, D).

###### Description of ♂ holotype

**(SDSU_TAC000671).** Carapace dusky cream to orange, conspicuous faint dark pigment behind ocular area, along carapace margin bleeding inwards. Legs pale yellow to cream. Abdomen mostly pale cream, with crisp paired lateral pigmentation blotches. All eyes approximately equal in size, except for AMEs, ~ 1/4 width of ALEs. Eyes with rings of dark pigment. CL 1.6, CW 1.3, abdomen length 2, total body length 3.6. Leg I total length 10.45 (3, 0.65, 3.1, 2.55, 1.15), leg formula 1423, leg I / CW ratio 8.0. Palp tegular apophysis with a 90-degree bend, distal end acute and blade-like. Lateral process of median apophysis concave, broadening and well-sclerotized along edge, distal process drawn into thin tip that closely parallels tegular apophysis tip. Paracymbium with strong ventral process, distal process consistent with species group (spatulate) and without other processes, dorsal process translucent blade of medium width, reaching above ventral process, weakly serrated at tip.

###### ♂ Variation.

The palp of the paratype male is similar to the holotype.

###### Description of ♀ paratype

**(SDSU_TAC000672).** Carapace dusky cream, very faint dark pigment behind ocular area, along carapace margin bleeding inwards. Legs pale yellow to cream. Abdomen with paired, lateral darker marking on a dirty gray background. Eyes approximately equal in size, except for AMEs, ~ 1/4 width of ALEs. Eyes with rings of dark pigment. CL = 1.2, CW 1.05, abdomen length 1.55, total body length 2.75. Leg I total length 8.1 (2.3, 0.55, 2.3, 2, 0.95), leg formula 1423, leg I / CW ratio 7.7. Epigynum, viewed laterally, with a prominent nose-shaped, cream-colored median septum, like other members of the species group. Viewed dorsally, dorsal-projecting portion of internal anterior lobes well sclerotized, rounded anteriorly and curving ventrally. Sclerotization making these appear as dark circles sitting above epigynum when viewed ventrally. Viewed dorsally, spermathecae below epigynal pocket, angled obliquely upwards, approximately banana-shaped.

###### ♀ Variation.

Adult females from the type locality vary in body size and in carapace and abdomen color (dark vs. light) but share a similar epigynum.

###### Distribution and natural history.

Known only from the type locality from along the Cane River, a tributary of the Nolichucky River. Adjacent collections have thus far only resulted in the collection of non-sister *Nesticustempletoni* (Fig. 30) and *N.paynei* further east (Fig. 13). We hypothesize that *N.canei* has a very small geographic distribution. More collecting effort in the immediate vicinity of the type locality is needed to understand the geographic extent of this apparently microendemic species.

Specimens from the type collection were found to be relatively common in void spaces beneath rocks in a small shaded boulderfield in roadside forest, at approximately 700 meters in elevation.

###### Etymology.

Named after the Cane River, a small north-flowing river found only in Yancey County, North Carolina.

###### Remarks.

Morphologically very similar to *Nesticusgertschi* and sister to this taxon on UCE trees (Figs 3, 4). This species is of conservation importance because of an apparently naturally small geographic distribution.

#### ﻿*barrowsi* group, including:

*Nesticusbondi* sp. nov.

*Nesticusbarrowsi* Gertsch, 1984

*Nesticuslowderi* sp. nov.

This small species group is strongly supported as monophyletic on both concatenated and coalescent phylogenomic trees (Figs 3, 4). Furthermore, the three species within this group are each strongly supported by nuclear phylogenomic data, with high gene and site CF values suggesting minimal gene tree variance within the group (Fig. 5). Each species is recovered with high support on the mitochondrial tree (Fig. 6), but the species group itself is polyphyletic, fragmented into three distantly related mitochondrial clades (Fig. 6).

**Figure 41.**
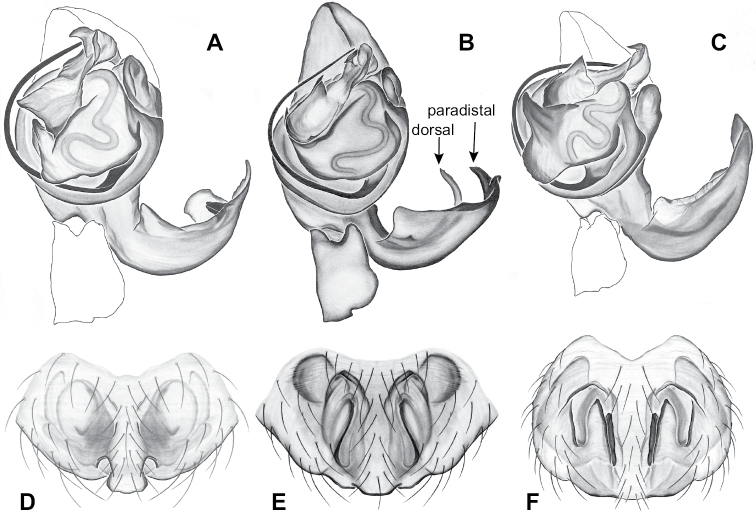
Comparative ♂♀ genitalia of *barrowsi* group; ♂ **A***Nesticusbondi***B***N.barrowsi***C***N.lowderi*; ♀ **D***N.bondi***E***N.barrowsi***F***N.lowderi*. All views ventral. See subsequent figures for specimen locations and voucher details.

Consistent with phylogenomic data, each species in this group is morphologically distinctive, easily separated by diagnostic features of both male and female genital morphology (Fig. 41). Overall, species delimitation within this group is straightforward, likely reflecting a relatively more ancient history (and extinction of intervening lineages) within the group. This situation parallels the *archeri* group, but is unique for a montane lineage of Appalachian *Nesticus*.

We do not identify diagnostic morphological features for the entire species group, as many aspects of both male and female morphology occur elsewhere in the combined lineages sister to the *barrowsi* group (i.e., larger clade including *barri*, *carteri*, and *reclusus* groups; Figs 3, 4).

**Figure 42.**
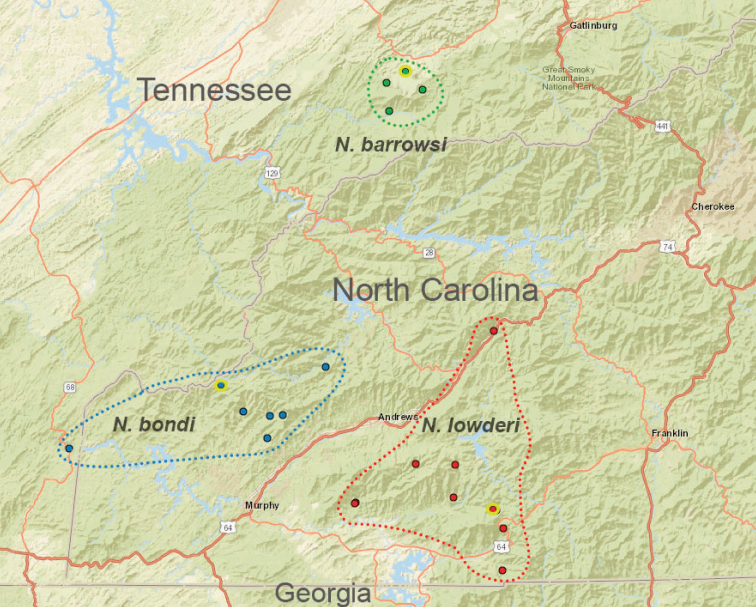
Distribution of the *barrowsi* group. Type localities designated with yellow circles. State boundaries and major cities shown for geographic context. Dashed lines circumscribe known species distributions.

Each of the species in this species group occupies a relatively small geographic distribution in three disjunct pockets of the far western Blue Ridge (Fig. 42). These disjunct pockets are separated by montane habitats occupied by members of the *reclusus* group (Fig. 53). As discussed below, all species in the *barrowsi* group appear to be naturally rare, which again parallels the *archeri* group.

##### ﻿ Nesticus bondi sp. nov.

Taxon classificationAnimaliaAraneaeNesticidae

7D139D38-87C9-5155-9169-829CF05946D8

https://zoobank.org/4E1DA202-2E5A-41DC-9936-323CFA01BE53

Fig. 43A–J

###### Material examined.

**Type material: *Holotype***: USA – **North Carolina, Cherokee Co.** • holotype ♂; along Tipton Creek, 1.2 mi. S NC/TN state line; 35.2503°N, -84.0724°W; 26 Aug. 2002; M. Hedin, M. Lowder, P. Paquin leg.; MCH 02_158; SDSU_TAC000665; ***Paratypes*.** – **North Carolina, Cherokee Co.** • 6♀; along Tipton Creek, 1.2 mi. S NC/TN state line; 35.2503°N, -84.0724°W; 26 Aug. 2002; M. Hedin, M. Lowder, P. Paquin leg.; MCH 02_158; **Non type material**: – **North Carolina, Cherokee Co.** • 5 imm; along Tipton Creek, 1.2 mi. S NC/TN state line; 35.2503°N, -84.0724°W; 26 Aug. 2002; M. Hedin, M. Lowder, P. Paquin leg.; MCH 02_158; • ♂, 5♀, 2 imm; Davis Creek Road, along Davis Creek, Snowbird Mountains, N of Grandview; 35.2151°N, -84.0368°W; 16 Aug. 2007; M. Hedin, M. McCormack, S. Derkarabetian leg.; MCH 07_110; • ♂, 2♀, 1 imm; Dinkin Cove Road, N of Hanging Dog Mountain; 35.1809°N, -83.9988°W; 16 Aug. 2007; M. Hedin, M. McCormack, S. Derkarabetian leg.; MCH 07_109; • ♂, 5♀; Hanging Dog Creek, below Hanging Gap; 35.2112°N, -83.9739°W; 17 Aug. 2004; M. Hedin, R. Keith, J. Starrett, S. Thomas leg.; MCH 04_055; • ♀; Hanging Dog Creek, E Boiling Springs; 35.2094°N, -83.9945°W; 17 Aug. 2004; M. Hedin, R. Keith, J. Starrett, S. Thomas leg.; MCH 04_056; USA – **North Carolina, Graham Co.** • ♂, 2 imm; along Snowbird Creek, near Wilson Cabin; 35.2733°N, -83.9051°W; 27 Aug. 2002; M. Hedin, M. Lowder, P. Paquin leg.; MCH 02_161; – **Tennessee, Polk Co.** • 1 imm (identification based on UCE and mitochondrial evidence); Hwy 68, vic Apalachia, just S Hiwassee River; 35.1676°N, -84.3159°W; 17 Aug. 2007; M. Hedin, M. McCormack, S. Derkarabetian leg.; MCH 07_111.

###### Diagnosis.

**Figure 43.**
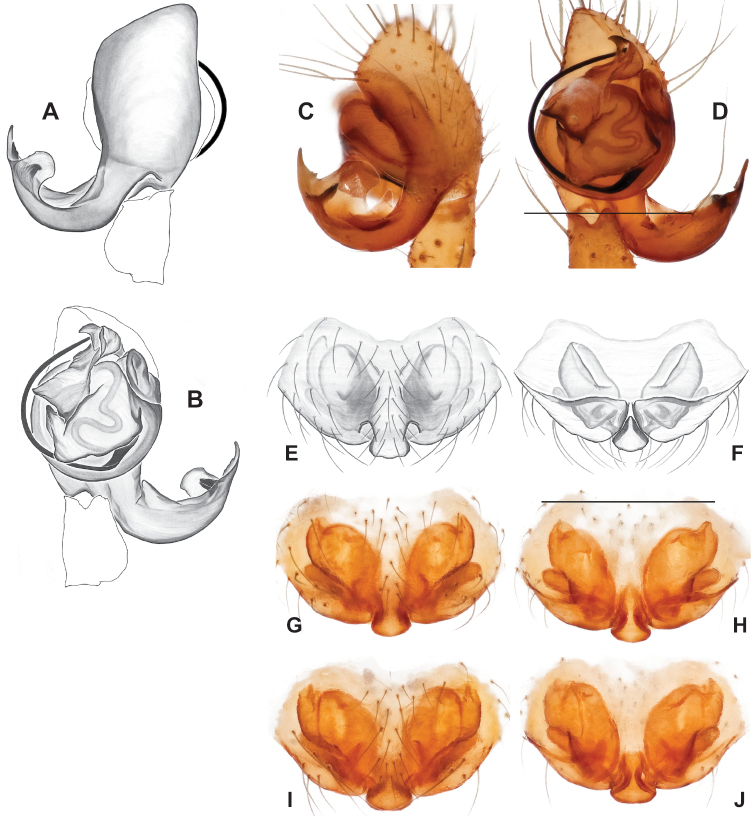
*Nesticusbondi* sp. nov. ♂ palp and ♀ epigynal variation. North Carolina, Cherokee Co., along Tipton Creek, MCH 02_158 (SDSU_TAC000665), ♂ palp, dorsal (**A**), ventral (**B**). North Carolina, Graham Co., along Snowbird Creek, MCH 02_161, ♂ palp, dorsal (**C**), ventral (**D**). Scale bar: 0.5 mm. North Carolina, Cherokee Co., along Tipton Creek, MCH 02_158 (SDSU_TAC000666), epigynum, ventral (**E**), dorsal (**F**). North Carolina, Cherokee Co., Dinkin Cove Road, MCH 07_109, epigynum, ventral (**G**), dorsal (**H**). North Carolina, Cherokee Co., Davis Creek Road, MCH 07_110, epigynum, ventral (**I**), dorsal (**J**). Scale bar: 0.5 mm.

Males are easily distinguished from other members of the species group by the unique shape of the median apophysis, the shape of the tegular apophysis and tegular keel, the shape of the dorsal paracymbial process, and possession of a thorn-shaped distomedial paracymbial process (Fig. 43A–D). Epigynal morphology, particularly the shape of the posterior extension of the median septum, is distinctive for the entire Appalachian clade (Fig. 43E–J).

###### Description of ♂ holotype

**(SDSU_TAC000665).** Carapace dusky cream to orange, faint gray pigmentation behind ocular area leading to midline and around edges. Legs pale yellow / cream. Abdomen with paired dark gray blotches on a light gray background. All eyes approximately equal in size, except for AMEs, ~ 1/4 width of ALEs. Eyes with rings of dark pigment. CL 1.3, CW 1.17, abdomen length 1.35, total body length 2.65. Leg I total length 9.8 (2.72, 0.53, 2.87, 2.58, 1.1), leg formula 1423, leg I / CW ratio 8.4. Paracymbium possesses a well-sclerotized thorn-shaped distomedial process. Paracymbial dorsal process a large transparent lobe that lacks a basal process, approximately contiguous with the distal paracymbial process, itself conspicuously weakly sclerotized, narrow, pointed, and weakly serrate along dorsal edge. Ventral paracymbial process triangular. Median apophysis somewhat triangular with a sclerotized point directed prolaterally. Tegulum with posterior keel; tegular process short, beak-like, narrows distally, and directed anteriorly. Distal tip of conductor bent and directed prolaterally.

###### ♂ Variation.

Males from different geographic locations show very minor variation in the width (at base) of the dorsal paracymbial process and depth of indentation between dorsal and distal processes (Fig. 43A–D).

###### Description of ♀ paratype

**(SDSU_TAC000666).** Carapace dusky cream to orange, gray pigmentation behind ocular area leading to midline and around edges. Legs pale yellow / cream. Abdomen with paired dark gray / black blotches on a light gray background. All eyes approximately equal in size, except for AMEs, ~ 1/4 width of ALEs. Eyes with rings of dark pigment. CL 1.4, CW 1.24, abdomen length 1.75, total body length 3.15. Leg I total length 8.32 (2.38, 0.54, 2.39, 2.01, 1), leg formula 1423, leg I / CW ratio 6.7. Epigynum with well-defined orifices lateral to a posterior extension of the median septum, itself widening posteriorly with a flattened posterior edge. Spermathecae elongated and directed anterolaterally. Posterolateral edges of epigynum folded over dorsally to form dorsal posterior flaps. Viewed dorsally, large, internal lobes extend anterolaterally with sclerotized rims.

###### ♀ Variation.

Females from different geographic locations show very minor variation in the shape of the anterior internal sclerotized epigynal lobes (Fig. 43E–J).

###### Distribution and natural history.

Most populations are from the southwestern flanks of the Snowbird Mountains of western North Carolina (Fig. 42). A single immature specimen is known from further west at Apalachia (placement based on UCE and mitochondrial evidence), suggesting that additional populations likely reside in the intervening montane habitats (Fig. 42).

At the type locality of Tipton Creek, *Nesticusbondi* (♂, 6♀) was found in syntopy with *N.sheari* (4♀); field notes read “*Nesticus* in boulderfield above road, north-facing, concentrated in small drainage”. Because we did not identify specimens directly in the field, it remains unclear if these different species were found side-by-side or were perhaps somehow segregated by microhabitat at this location. At Davis Creek (MCH 07_110), *Nesticus* were found “under rocks at streamside – many from webs under a large rock shelter cave”.

###### Etymology.

Named after Dr. Jason Bond, Professor and Schlinger Chair of Insect Systematics at the University of California Davis. Jason was born in the southern Appalachians, schooled in the mountains of western North Carolina, and perhaps sometimes paddled in the Snowbird Mountains. Jason has been a longtime close friend and arachnological colleague of MH and is for him forever a source of scientific (and life) inspiration.

###### Remarks.

The immature specimens from Tipton Creek are here attributed to *Nesticusbondi*, but some (or all) could be *N.sheari*.

##### ﻿ Nesticus barrowsi

Taxon classificationAnimaliaAraneaeNesticidae

Gertsch, 1984

8D969825-BFA8-5396-B06E-5A3E57D02852

Figs 44A, B , 45A–H

Nesticus barrowsi Gertsch, 1984: 35, figs 103–105, 118–120; Reeves 2000: 338.

###### Material examined.

**Type material: *Holotype***: USA – **Tennessee, Blount Co.** • ♂ holotype; Tuckaleechee Caverns, Tuckaleechee Cove; 1 Nov. 1938; W.B. Jones leg.; AMNH. **New collections from type locality**: – **Blount Co.** • ♀; Tuckaleechee Caverns, Tuckaleechee Cove; 22 Sep. 1992; M. Hedin, S. O’Kane leg. **Non type material**: – **Blount Co.** • ♂, 7♀; Great Smoky Mountains NP, Gregory Cave, Cades Cove; 21 Aug. 1992; M. Hedin leg.; • 2♀; Great Smoky Mountains NP, Rich Mountain Blowhole, Calf Cave; 2 Aug. 2000; M. Hedin, J. Cokendolpher, W. Reeves leg.; MCH 00_147; • ♀; Great Smoky Mountains NP, White Oak Sinks, Rainbow Cave; 21 Aug. 1992; M. Hedin leg.

###### Diagnosis.

**Figure 44.**
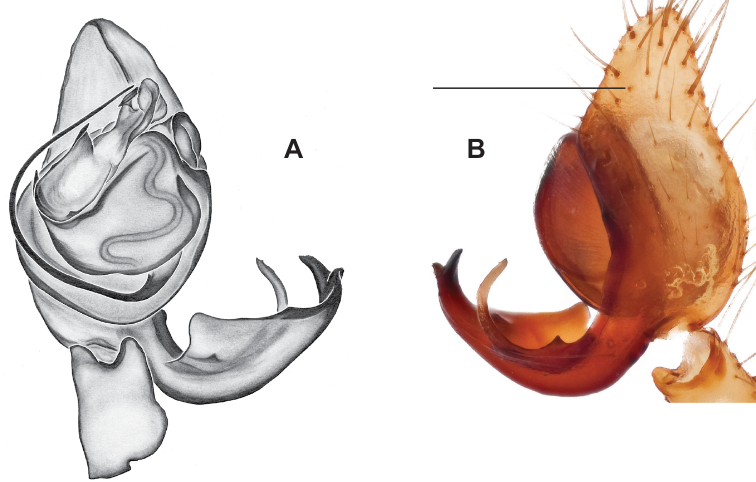
*Nesticusbarrowsi* ♂ palps. Tennessee, Blount Co., Great Smoky Mountains NP, Gregory Cave, MCH specimen #1295, ventral (**A**), dorsal (**B**). Scale bar: 0.5 mm.

**Figure 45.**
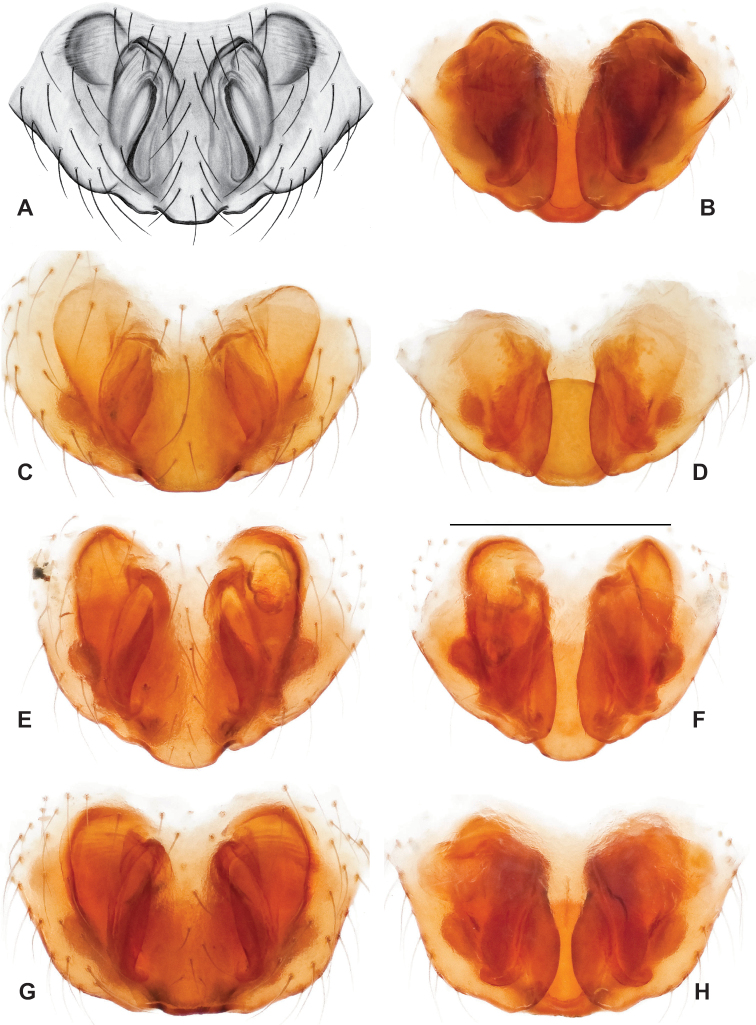
*Nesticusbarrowsi* epigynal variation. Tennessee, Blount Co., Great Smoky Mountains NP, Calf Cave, MCH 00_147, ventral (**A**), dorsal (**B**). Tennessee, Blount Co., Great Smoky Mountains NP, White Oak Sinks, Rainbow Cave, MCH specimen #1303, ventral (**C**), dorsal (**D**). Tennessee, Blount Co., Tuckaleechee Caverns, MCH specimen #1568, ventral (**E**), dorsal (**F**). Tennessee, Blount Co., Great Smoky Mountains NP, Gregory Cave, MCH specimen #1297, ventral (**G**), dorsal (**H**). Scale bar: 0.5 mm.

The diagnosis of Gertsch (1984) is revised here to recognize the phylogenetic affinities within the *barrowsi* group. *Nesticusbarrowsi* is troglomorphic (long-legged, pale, approximately eyeless, relatively large-bodied), unlike other species in the species group. The male tegular apophysis curves to lie behind a quadrate median apophysis, is sharply tipped, without a basal keel. The paracymbial dorsal process is translucent, skinny and finger-like, while the distal process includes a well-sclerotized pointed tip and a small ventral keel (Fig. 44A, B). Female *N.barrowsi* differ from other members of the species group in overall morphology of the epigynum, including the pear-shaped spermathecae (Fig. 45A–H).

###### Variation.

Minor variation is observed in the height and width of the paired epigynal plates across geographic locations (Fig. 45A–H).

###### Distribution and natural history.

This troglomorphic species is only known from caves in karst windows along the northwestern edge of Great Smoky Mountains National Park (Cades Cove, Tuckaleechee Cove; Fig. 42). Reeves (2000) reported *Nesticusbarrowsi* in sympatry with *N.stupkai* at two cave locations in Great Smoky Mountains National Park. We also collected these species in near syntopy at White Oak Sinks, with *N.barrowsi* found in the dark zone of caves and *N.stupkai* found closer to cave entrances (twilight zone).

##### ﻿ Nesticus lowderi sp. nov.

Taxon classificationAnimaliaAraneaeNesticidae

EB812E0C-B622-5172-894C-DE2D829B2BD9

https://zoobank.org/1F0FB7CB-C0AD-4284-B1CA-F44AC6C9E160

Figs 46A–D , 47A–H

###### Material examined.

**Type material: *Holotype***: USA – **North Carolina, Clay Co.** • ♂ holotype; Chunky Gal Mountain, Chestnut Branch of Barnard’s Creek; 35.0857°N, -83.6327°W; 6 May. 1999; M. Hedin, B. Dellinger leg.; MCH 99_016 (SDSU_TAC000667); ***Paratypes***: – **Clay Co.** • 3♀; data as for holotype; **Non type material**: – **Clay Co.** • ♂; along Barnard’s Creek, N side of Chunky Gal Mountain; 35.0868°N, -83.6372°W; 24 Apr. 1992; B. Dellinger leg.; • ♀; Eagle Fork Creek (Dave Barrett) SE of Shooting Creek N of Hightower Bald; 35.0075°N, -83.6225°W; 20 Aug. 2002; M. Hedin, F. Coyle, M. Lowder, P. Paquin leg.; MCH 02_143; • ♂, 6♀; Fires Creek Road, Picnic Area along Fires Creek; 35.0955°N, -83.8586°W; 16 Aug. 2007; M. Hedin, M. McCormack, S. Derkarabetian leg.; MCH 07_108; • 3♂, 5♀; Fires Creek, Long Branch, just up from Short Branch; 35.1467°N, -83.7618°W; 21 Aug. 2002; M. Hedin, F. Coyle, M. Lowder, P. Paquin leg.; MCH 02_144; • 2♀, 1 imm; Fires Creek, near Leatherwood Falls, just NE Fires Creek Picnic Area; 35.0961°N, -83.8566°W; 18 Aug. 2004; M. Hedin, R. Keith, J. Starrett, S. Thomas leg.; MCH 04_060; • 2♀; FR 440, along Big Tuni Creek, 2 mi. N Woods Road; 35.1025°N, -83.7007°W; 16 Aug. 2007; M. Hedin, M. McCormack, S. Derkarabetian leg.; MCH 07_107; • ♂, 7♀, 7 imm; FR 440, Big Tuni Creek, E Tusquitee Bald near Bob Allison Picnic Area; 35.1463°N, -83.6974°W; 30 Aug. 2002; M. Hedin, M. Lowder, P. Paquin leg.; MCH 02_171; • ♂; W side Chunky Gal Mountain, Hwy 64, near scenic overlook; 35.0627°N, -83.6204°W; 6 May. 1999; M. Hedin, B. Dellinger leg.; MCH 99_019; – **Swain Co.** • 2♀; Nantahala River Gorge, Blowing Springs Cave; 10 Sep. 2001; J.D. Mayes leg.

###### Diagnosis.

**Figure 46.**
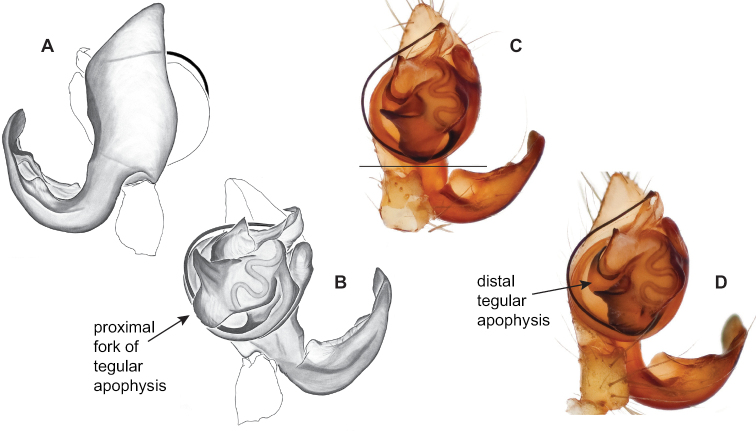
*Nesticuslowderi* sp. nov. ♂ palps. North Carolina, Clay Co., Chunky Gal Mountain, Chestnut Branch of Barnard’s Creek, MCH 99_016 (SDSU_TAC000667), dorsal (**A**), ventral (**B**) **C** North Carolina, Clay Co., Big Tuni Creek, MCH 02_171, ventral **D** North Carolina, Clay Co., Fires Creek Road, Picnic Area along Fires Creek, MCH 07_108, ventral. Scale bar: 0.5 mm.

Several male features distinguish *Nesticuslowderi* from other members of the species group (and Appalachian clade), including the distinctive shape of the posterior keel of the forked tegular apophysis and the low sinuous paradistal process (Fig. 46A–D). Median bars, extending V-shaped upwards from the median septum and interrupting the epigynal pockets, diagnose *N.lowderi* females from other members of the species group and other common regional taxa (e.g., *N.reclusus*).

###### Description of ♂ holotype

**(SDSU_TAC000667).** Carapace cream-colored, faint gray pigmentation behind ocular area leading to midline. Legs pale yellow / cream. Abdomen with many dark gray blotches on a pale cream background. All eyes approximately equal in size, except for AMEs, ~ 1/2 width of ALEs. Eyes with rings of dark pigment. CL 1.32, CW 1.2, abdomen length 1.48, total body length 2.8. Leg I total length 9.88 (2.71, 0.58, 2.9, 2.59, 1.1), leg formula 1423, leg I / CW ratio 8.2. Ventral paracymbial process consists of a large, basal lobe that broadens down length of paracymbium. Distal process somewhat spoon-shaped, dorsal process a low lobe, and the paradistal process consists of a sinuous, prolaterally directed extension with a heavily sclerotized anterior edge. Median apophysis rectangular with an anteriorly directed point and a sclerotized prolateral edge. Tegulum forked, with strong posterior keel including a wide lobe with a flattened edge. Distal tegular process crescent-shaped with a heavily sclerotized point directed anterolaterally, closely appressed to median apophysis. Distal tip of conductor bent and directed prolaterally.

###### ♂ Variation.

Males from different locations varied slightly in the shape of the basal fork of the tegular apophysis (Fig. 46A–D).

###### Description of ♀ paratype

**Figure 47.**
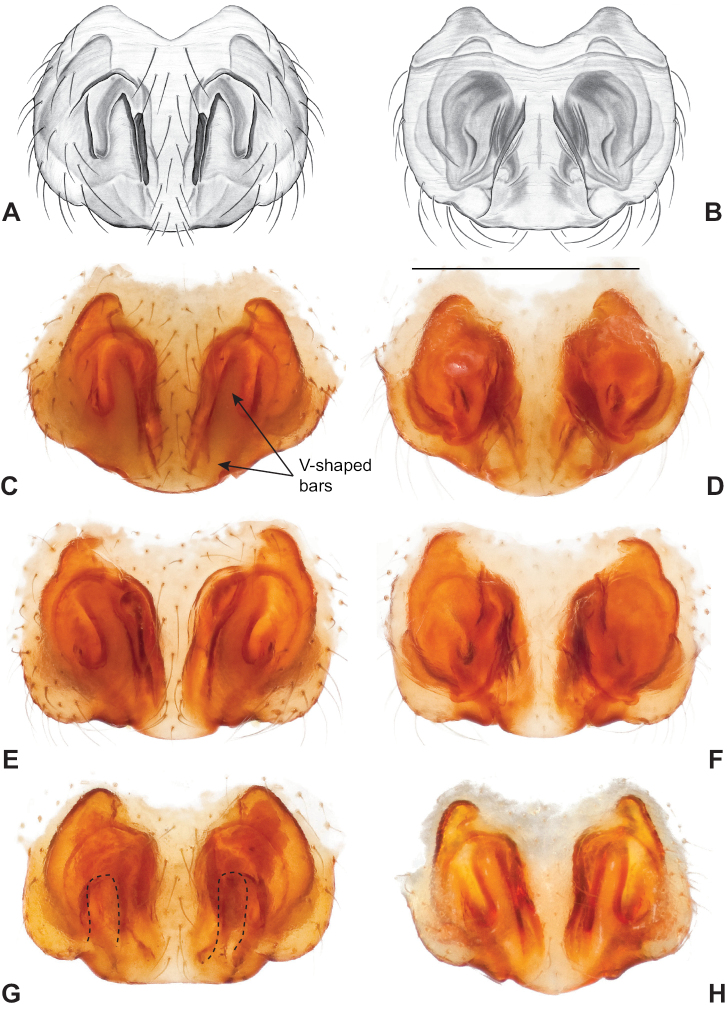
*Nesticuslowderi* sp. nov. epigynal variation. North Carolina, Clay Co., Chunky Gal Mountain, Chestnut Branch of Barnard’s Creek, MCH 99_016 (SDSU_TAC000668) ventral (**A**), dorsal (**B**). North Carolina, Clay Co., Big Tuni Creek, MCH 02_171, ventral (**C**), dorsal (**D**). Swain Co., Blowing Springs Cave, ventral (**E**), dorsal (**F**). North Carolina, Clay Co., Fires Creek Road, Picnic Area along Fires Creek, MCH 07_108, ventral (**G**). North Carolina, Clay Co., Dave Barrett Fork of Eagle Fork Creek, MCH 02_143, ventral (**H**). Scale bar: 0.5 mm. Septal bars outlined in image **G** to better reflect actual specimen.

**(SDSU_TAC000668).** Carapace dusky cream to orange, with faint gray pigmentation behind ocular area leading to midline and around edges. Leg pale yellow / cream. Abdomen with paired dark gray blotches on a light gray background. All eyes approximately equal in size, except for AMEs, ~ 1/2 width of ALEs. Eyes with rings of dark pigment. CL 1.25, CW 1.11, abdomen length 1.46, total body length 2.71. Leg I total length 8.34 (2.41, 0.51, 2.39, 2.04, 0.99), leg formula 1423, leg I / CW ratio 7.5. Epigynal pockets interrupted by median bars that extend upwards V-shaped from base of median septum to nearly the top of the larger pocket (giving an overall appearance of an anchor, Fig. 47A–H). The presence of these bars forms septal grooves that lie directly adjacent to the median septum, and smaller pockets lateral to the V-shaped bars. Median septum slightly protruding posteriorly past lateral lobes. Spermathecae elongated and curved along lateral borders of epigynum, approximately banana-shaped. Ventrolateral sides of epigynal plate bulging (convex), as viewed from the side. Viewed dorsally, large internal lobes extend anteriorly and possess sclerotized rims. Interior margins directed inward diagonally towards the center of the epigynum.

###### ♀ Variation.

Epigynal structure fairly uniform across collecting locations (Fig. 47A–H). Blowing Springs Cave females are concolorous and relatively long-legged.

###### Distribution and natural history.

Most populations are from the Chunky Gal, Tusquitee, and Valley River Mountains of western North Carolina (Fig. 42). Holler et al. (2020) attributed female specimens from Blowing Springs Cave to *Nesticuscooperi* (= *N.reclusus*); we instead have identified these as *N.lowderi* based on epigynal morphology, including the inward curve of the internal plates (Fig. 47A–H). This would represent a disjunct northern-most record for *N.lowderi* (Fig. 42) and should be confirmed with the collection of males and /or nuclear DNA data from this location. Another possibility is sympatry at this location.

At Fires Creek (MCH 02_144), *Nesticuslowderi* (3♂, 5♀) was found in syntopy with *N.reclusus* (♂, 4♀); field notes read “30-minute survey, 3 persons, S-facing and N-facing rock fields”. Because we did not identify specimens directly in the field it remains unclear if these different species were truly syntopic or were segregated somehow at this location. Also, at least 15 immatures were collected at this location but were not identified to species because of sympatry.

Collection records suggest that this species is less common in the Chunky Gal Mountains than in the more westerly Tusquitee and Valley River Mountains.

###### Etymology.

This species is named to recognize and honor Michael Lowder, faculty member at Stanly Community College, native North Carolinian, fan of western North Carolina, and collector of many Appalachian *Nesticus*. Michael was the first graduate student of MH, who remains forever grateful for our continued friendship and reflects on our early lab and field time together with great fondness.

###### Remarks.

The extent of mitochondrial divergence observed in this taxon over a small geographic region (including only Chunky Gal, Tusquitee, and Valley River Mountains) is notable (Fig. 6).

#### ﻿*barri* group, including:

*Nesticusbarri* Gertsch, 1984

*Nesticusfurtivus* Gertsch, 1984

This group includes the sister species *Nesticusbarri* and *N.furtivus*, a clade supported by nuclear phylogenomics (Figs 3, 4) but not mitochondrial evidence (Fig. 6). Each species is phylogenomically distinctive, with minimal UCE gene tree conflict as evidenced by high gene and site CF values (Fig. 5).

The unique morphology of each species is discussed below. We do not attempt to identify diagnostic morphological features for this small species group.

*Nesticusbarri* and *N.furtivus* are cave-dwelling species from the Tennessee / Alabama / Georgia (TAG) region, the former conspicuously widespread for a cave-restricted species, while *N.furtivus* is narrowly endemic to limestone caves from a single mountain (Fig. 49).

##### ﻿ Nesticus barri

Taxon classificationAnimaliaAraneaeNesticidae

Gertsch, 1984

7A19D16D-7EA3-56E2-ABE9-989E1B233E69

Fig. 48A, C

Nesticus barri Gertsch, 1984: 36, figs 121–123, 161–163; Hedin and Dellinger 2005: 3, figs 2–10; Snowman et al. 2010: fig. 1; Carver et al. 2016: fig. 2.
Nesticus valentinei Gertsch, 1984: 29, figs 150–152.

###### Material examined.

**Non type material**: USA – **Alabama, Jackson Co.** • ♀; Fern Cave, AJK597; 1 Dec. 2018; M.L. Niemiller, M.E. Slay, T. Inebnit, J. Pinkley, J. Lamb, P. Pattavina, K. Sapkota, B. Miller, N. Mann leg.; MLN 18–051.8; • ♀, 1 imm; Fern Cave, AJK597, bottom of cave; 1 Aug. 2008; J. Pinkley leg.; JP 08–AJK597.1; • ♀; Fern Cave, AJK597, Johnston entrance; 2 Jun. 2018; M.L. Niemiller, M.E. Slay, T. Inebnit, B. Miller, et al. leg.; MLN 18–020.8; • ♀; Fern Cave, AJK597, Morgue – past first Bat Room; 23 Jun. 2018; A. Hinkle, S. Pitts leg.; AH 18–001.2; • ♀; Fern Cave, AJK597, upper formation passage; 2 Jun. 2018; M.L. Niemiller, M.E. Slay, T. Inebnit, B. Miller, et al. leg.; MLN 18–020.26; • ♀; Fern Cave, AJK597, upper north passage; 3 Jun. 2018; M.L. Niemiller, M.E. Slay, T. Inebnit, B. Miller, et al. leg.; MLN 18–021.1; • 8♀; Guess Creek Cave, E Trenton; 25 Sep. 1992; M. Hedin, J. Hedin, S O’Kane leg.; • 2♀, 1 imm; Moody Cave, AJK1189; 18 Mar. 2019; M.L. Niemiller, J. Lamb, A. Hinkle leg.; MLN 19–014.20; • ♀, 1 imm; Tumbling Rock Cave, AJK171; 8 Mar. 2014; M.L. Niemiller, C.D.R. Stephen, K.S. Zigler, R. Miller, C. Borer, C. Maddux, J. Clark, V. Leray leg.; MLN 14–011.10; – **Alabama, Marshall Co.** • 9♀; Bishop Cave, N of Guntersville Dam; 25 Sep. 1992; M. Hedin, J. Hedin, S O’Kane leg.; • ♂, 2♀; Bishop Cave; 17 Aug. 2005; M. Hedin, R. Keith, J. Starrett, S. Thomas leg.; MCH 05_056; – **Tennessee, Franklin Co.** • ♂, 8♀; Keith Cave, S of Cowan; 24 Mar. 1995; M. Hedin, J. Hedin leg.; • 2♀, 2 imm; Little Crow Creek Cave, TFR15; 20 Sep. 2008; M.L. Niemiller, BT Miller, J Miller, N. Mann leg.; MLN 08–041; • ♂, 3♀; Lost Cove Cave, N/NE of Sherwood; 23 Sep. 1992; M. Hedin, J. Hedin, S O’Kane leg.; • 2♂, 7♀; Salt River Cave, W of Gonce, Alabama; 24 Mar. 1995; M. Hedin, J. Hedin leg.; • 2♀, 1 imm; Sinking Cove Cave, TFR25; 15 Oct. 2016; N.S. Gladstone leg.; NSG 16–TFR25.10; – **Tennessee, Marion Co.** • ♂, 2♀; Tate Spring Cave, SE of Monteagle; 15 Aug. 2004; M. Hedin, L. Hedin, R. Keith, J. Starrett, S. Thomas leg.; MCH 04_050.

###### Diagnosis.

**Figure 48.**
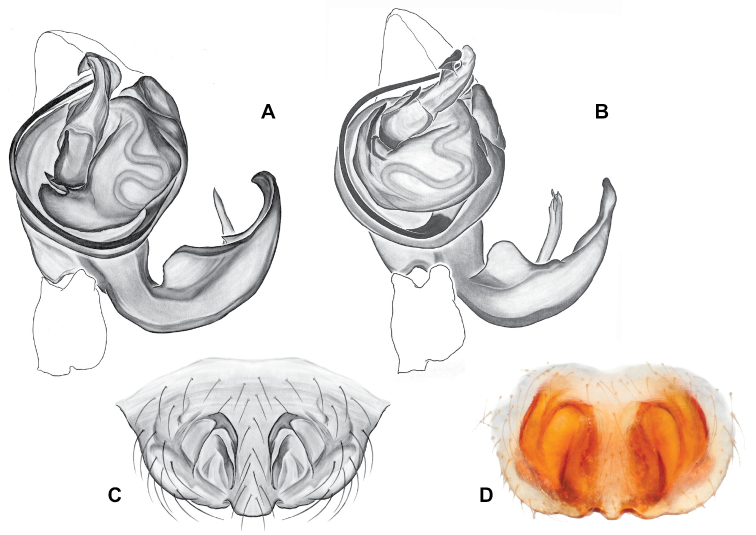
*Nesticusbarri* and *N.furtivus* genitalia. *N.barri* – Tennessee, Marion Co., Tate Spring Cave, MCH 04_050, ♂ palp ventral (**A**), epigynum ventral (**C**). *N.furtivus* – Tennessee, Hamilton Co., Raccoon Mountain Caverns, SE Chattanooga, MCH 00_137, ♂ palp ventral (**B**), epigynum ventral (#1660) (**D**).

The diagnosis of Gertsch (1984) is here modified to reflect a close phylogenetic relationship to *Nesticusfurtivus*. These taxa share an overall similarity in features of the male paracymbium and shape of the median apophysis but differ in the shape of the tegular apophysis (Fig. 48A–D). In *N.barri* the tegulum is forked, with a basal projection shaped like a curved blade, and with a distal crescent-shaped tegular process lying close behind the quadrate median apophysis. The epigynum of *N.barri* is similar to that of distant relative *N.lowderi* in general structure (Fig. 47A–H), but with internal plates (viewed dorsally) not as long. Females are distinctly different from *N.furtivus*, as discussed below in the diagnosis for this latter species.

###### Variation.

The shape of the basal tegular fork varies notably across cave locations. One male from Salt River Cave (MCH #2105) completely lacked a dorsal paracymbial process, without evidence that this was broken off. Variation in *Nesticusbarri* epigynal morphology was illustrated in Hedin and Dellinger (2005), figs 2–8.

###### Distribution and natural history.

**Figure 49.**
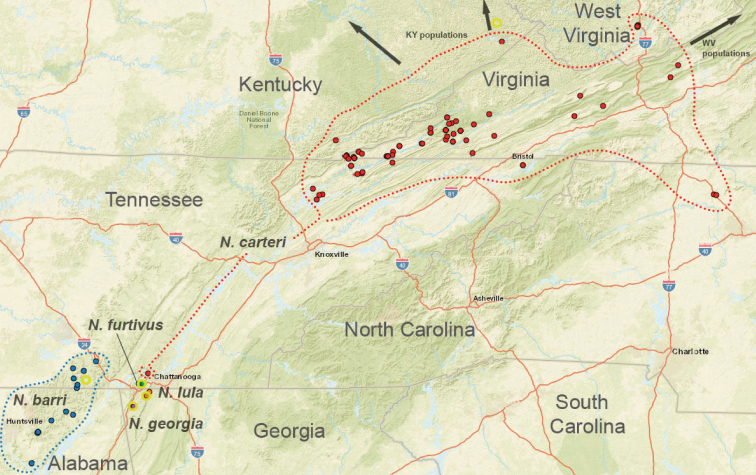
Distribution of *barri* and *carteri* groups. Type localities designated with yellow circles. State boundaries and major cities shown for geographic context. Dashed lines circumscribe known species distributions.

Known from possibly hundreds of caves in northwest Alabama and south-central Tennessee (Fig. 49; Hedin and Dellinger 2005: fig. 1; Snowman et al. 2010: fig. 1; Carver et al. 2016: fig. 2). Carver et al. (2016) reported on the reproductive biology of this species.

###### Remarks.

Based on consideration of morphology Hedin and Dellinger (2005) hypothesized that troglomorphic spiders from Tate Spring Cave, Tennessee represented a northern population of *Nesticusbarri*. The mitochondrial data included here further support this hypothesis (Fig. 6).

##### ﻿ Nesticus furtivus

Taxon classificationAnimaliaAraneaeNesticidae

Gertsch, 1984

F23B201B-A20D-5030-91A5-F9550C12187C

Fig. 48B, D

Nesticus furtivus Gertsch, 1984: 27, figs 97–99; Hedin and Dellinger 2005: 12, figs 15, 16.

###### Material examined.

**New collections from type locality**: USA – **Tennessee, Hamilton Co.** • ♀; Raccoon Mountain Caverns, se Chattanooga; 28 Mar. 1993; M. Hedin, M. Wolinsky leg.; • ♂; Raccoon Mountain Caverns; 25 Jul. 2000; M. Hedin, D. Wood, B. Delllinger, S. Perlacky leg.; MCH 00_137; • ♀; Raccoon Mountain Caverns; 19 Aug. 2005; M. Hedin, R. Keith, J. Starrett, S. Thomas leg.; MCH 05_063; **Non type material**: – **Marion Co.** • ♀; Hugden Branch Cave (TMN 127); 17 Apr. 2016; K.S. Zigler, P.R. Heald leg.; KSZ 15–570.

###### Diagnosis.

Closely related to *Nesticusbarri*, but the males differ in that the tip of the *N.furtivus* paracymbial dorsal process is finely forked, the shape of the basal tegular fork is broader (rather than blade-like), and the apical tegular fork is reduced and lacking a distinct tip (Fig. 48B). Female *N.furtivus* have a distinctly wide median septum that narrows to a conspicuous tip posteriorly (Fig. 48D).

###### Variation.

The Hugden Branch Cave female specimen, representing the second known location for this species, is troglomorphic with an epigynum that closely matches females from the type locality.

###### Distribution and natural history.

This troglomorphic species is known from two nearby caves from a single mountain in southeastern Tennessee, near Chattanooga (Fig. 49; Hedin and Dellinger 2005: fig. 1; Carver et al. 2016: fig. 2).

Carver et al. (2016) provide important natural history, reproductive biology, and abundance data for this rare species, extending earlier observations of Hedin and Dellinger (2005).

#### ﻿*carteri* group, including:

*Nesticuscarteri* Emerton, 1875

*Nesticusgeorgia* Gertsch, 1984

*Nesticuslula* Zigler & Milne, 2022

This small species group is strongly supported as monophyletic on both concatenated and coalescent phylogenomic trees (Figs 3, 4). The sister species *Nesticusgeorgia* and *N.lula* are recovered together with high support on the mitochondrial tree (Fig. 6), but they are separate from *N.carteri* populations, the latter fragmented into three distantly related mitochondrial clades (Fig. 6). This is one of the most notable examples of mitonuclear discordance in the Appalachian clade, here hypothesized to result from a lack of phylogenetic signal in the mitochondrial data at greater phylogenetic depths.

The unique morphology of each species is discussed below; we otherwise do not attempt to identify diagnostic morphological features for this small species group.

This group includes the geographically widespread *Nesticuscarteri*, and short-range endemic sister species from caves of northwestern Georgia (*N.georgia*, *N.lula*). An intriguing southern population of *N.carteri* (Pitchfork Cave), which is highly disjunct from any other known *N.carteri* population, might bridge this biogeographic gap (Fig. 49).

##### ﻿ Nesticus carteri

Taxon classificationAnimaliaAraneaeNesticidae

Emerton, 1875

EFC7AF57-8A4B-59F7-92F8-C8986DFC8F3A

Fig. 50A–J

Nesticus carteri Emerton, 1875: 279, pl. 1, fig. 28; Gertsch 1984: 34, figs 124, 125, 175–177.

###### Material examined.

**New collections from near type locality**: – **Kentucky, Carter Co.** • 3♂, 13♀; Laurel Cave, Carter Caves State Park, 10 mi NE Olive Hill; 15 Sep. 1992; M. Hedin, S. O’Kane leg.; **Non type material**: – **Indiana, Crawford Co.** • ♂, ♀; Heron Cave, ca. 7 mi. S of Leavenworth; 12 Sep. 1996; J. J. Lewis leg.; • 9♀; Wallier Cave; 26 Apr. 1997; J. J. Lewis leg.; – **Kentucky, Pike Co.** • ♂, ♀; Lick Creek County Park, N of Hwy 460, NE of Belcher; 37.3996°N, -82.3057°W; 26 Jun. 2014; M. Hedin leg.; MCH 14_008; – **North Carolina, Surry Co.** • ♂; just E of Pilot Mtn State Park, Pilot Knob Park Rd; 36.3415°N, -80.4538°W; 1 Jun. 2016; M. Hedin, S. Derkarabetian, J. Starrett, M. Lowder leg.; MCH 16_036; • ♂, 6♀; Pilot Mtn State Park, near campground; 36.3479°N, -80.4732°W; 31 May. 2016; M. Hedin, S. Derkarabetian, J. Starrett, M. Lowder leg.; MCH 16_035; – **Tennessee, Claiborne Co.** • 2♀; Sour Kraut Cave, TCB46; 1 Jun. 2015; M.L. Niemiller, E.T. Carter, L.E. Hayter leg.; MLN 15–009.10; • ♀, 1 imm; Station Creek Cave, CGNHP; 6 Jun. 2019; K.S. Zigler, L.E. Trumbore leg.; KSZ 19–102; • 2♀; English Cave, 0.9 mi. S Hamilton School; 25 Sep. 1991; M. Hedin, K. Crandall leg.; • 10♀; English Cave, 20 Sep. 1992; M. Hedin, S. O’Kane leg.; • ♀; Kings Saltpeter Cave, TCB52; 30 May. 2015; M.L. Niemiller, C.D.R. Stephen, E.T. Carter, A.S. Engel, S. Engel, P.B. Hart leg.; MLN 15–008.34; • 2♀, 2 imm; Coonsies Creek Cave, TCB57; 23 Mar. 2016; M.L. Niemiller, C.D.R. Stephen leg.; MLN 16–023.13; – **Tennessee, Hamilton Co.** • 3♂, 7♀; N of Tiftonia, near Pitchfork Cave; 22 Sep. 1992; M. Hedin leg.; – **Tennessee, Hancock Co.** • ♂, 6♀; Hwy 63, S Mulberry Gap; 36.5659°N, -83.2465°W; 21 Aug. 2005; M. Hedin, R. Keith, J. Starrett, S. Thomas leg.; MCH 05_070; – **Tennessee, Sullivan Co.** • 12♀; Bristol Caverns, SE of Bristol; 18 Sep. 1992; M. Hedin, S. O’Kane leg.; • ♀, 1 imm; Bristol Caverns, TSL1; 17 Oct. 2017; N.S. Gladstone leg.; NSG 17–TSL1.9; – **Tennessee, Union Co.** • 2♀; Big Cave, TUN10; 22 Mar. 2015; M.L. Niemiller, C.D.R. Stephen leg.; MLN 15–005.18; • 5♀; Oaks Cave, TUN5; 23 Mar. 2015; M.L. Niemiller, C.D.R. Stephen, E.T. Carter, LE Hayter leg.; MLN 15–007.3; • ♂, 3♀; Rogers Hollow Cave, TUN23; 22 Mar. 2015; M.L. Niemiller, C.D.R. Stephen leg.; MLN 15–002.6; • 3♀; Wright Cave, TUN9; 21 Mar. 2015; M.L. Niemiller, C.D.R. Stephen, E.T. Carter, JP McClendon leg.; MLN 15–001.10; – **Virginia, Giles Co.** • 2♀; Salamander Cave, CGNHP; 26 Jul. 2019; K.S. Zigler, L.E. Trumbore leg.; KSZ 19–169; • ♀, 10 imm; Sugar Run Cave System, Birthday Entry; 27 Aug. 2018; T. Malabad leg.; – **Virginia, Lee Co.** • 3♀, 5 imm; Bacon Cave; 8 Mar. 2017; T. Malabad leg.; • ♀, 5 imm; Bacon Cave; 21 Mar. 2018; T. Malabad leg.; • ♀; Bacon Cave; 15 Nov. 2019; T. Malabad leg.; • 2♀; Bacon Cave; 22 Oct. 2019; T. Malabad, K. Kosič Ficco leg.; • ♂; Bacon Cave; 3 Mar. 2020; T. Malabad, K. Kosič Ficco, R. Blackwell, L. Young leg.; • ♀; Bacon Cave; 10 Mar. 2021; T. Malabad, W. Orndorff, Z. Orndorff leg.; • 2♂, 15♀; Bowling Cave, SW of Pineville; 19 Sep. 1992; M. Hedin, S. O’Kane leg.; • ♂1 imm; Burja Cave; 29 Apr. 2017; T. Malabad leg.; • 1 imm; Burja Cave; 1 Jul. 2017; T. Malabad leg.; • ♂; Burja Cave; 19 Aug. 2017; T. Malabad leg.; • ♂; Burja Cave; 1 Dec. 2018; T. Malabad leg.; • 6♀; Cave Spring Recreational Area, NE of Dryden; 36.8033°N, -82.921°W; 21 Aug. 2005; M. Hedin, R. Keith, J. Starrett, S. Thomas leg.; MCH 05_071; • 2♂, 7♀; Cumberland Gap National Historic Park, Skylight Cave; 20 Sep. 1992; M. Hedin, S. O’Kane leg.; • 2♀, 2 imm; Gallohan No. 2 Cave; 29 Jan. 2018; T. Malabad leg.; • ♂1 imm; Gap Cave, CGNHP; 31 Aug. 2019; K.S. Zigler, et al. leg.; KSZ 19–235; • 2♀; Gibson Frazier Cave, 8 miles southwest of Jonesville, VA; 20 Nov. 2019; T. Malabad, R. Blackwell leg.; • 4♀, 2 imm; Indian Burial Cave; 30 Jan. 2018; T. Malabad leg.; • ♂, ♀, 3 imm; Indian Cave, CGNHP; 5 Jun. 2019; K.S. Zigler, L.E. Trumbore leg.; KSZ 19–165; • ♀, 2 imm; Indian Cave, CGNHP; 9 Jul. 2019; K.S. Zigler, L.E. Trumbore leg.; KSZ 19-78; • 2♀, 2 imm; Little Saltpeter Cave, CGNHP; 11 Jul. 2019; K.S. Zigler, L.E. Trumbore leg.; KSZ 19–13; • 2♀; Litton Cave No. 1, 4.8 miles west of Stickleyville, VA; 10 Mar. 2021; T. Malabad, W. Orndorff, Z. Orndorff leg.; • ♂, 5♀; Litton Cave No. 2, 6.3 miles east of Jonesville, VA; 24 Mar. 2021; T. Malabad, W. Orndorff leg.; • 2♀, 2 imm; Pack Rat Cave, CGNHP; 10 Jul. 2019; K.S. Zigler, LE Trumbore leg.; KSZ 19–60; • ♀; Robertson Cave No. 1, 1.75 miles northeast of Wheeler, VA; 17 Sep. 2020; T. Malabad, K. Kosič Ficco, M. Ficco leg.; • ♀; Robertson Cave No. 2, 1.75 miles northeast of Wheeler, VA; 28 Apr. 2021; T. Malabad, K. Kosič Ficco, W. Orndorff, M. Ficco leg.; • 8♀; Secret Cave, 1.3 miles southeast of Dryden, VA; 11 Mar. 2021; T. Malabad, W. Orndorff, Z. Orndorff leg.; • ♂, 3♀; Secret Cave; 22 Apr. 2021; T. Malabad, W. Orndorff, Z. Orndorff, J. Lewis, L. Young leg.; • 2♀, 3 imm; Skylight Cave, CGNHP; 5 Jun. 2019; K.S. Zigler, LE Trumbore leg.; KSZ 19–132; • 6 imm; Spangler Cave, west of Jonesville, VA; 30 Jan. 2018; T. Malabad leg.; • 5♀; Spangler Cave; 27 Jan. 2020; T. Malabad, R. Blackwell, Rick Reynolds leg.; • ♀, 1 imm; Young–Fugate Cave, southwest of Wheeler, VA; 26 Aug. 2015; W. Orndorff leg.; • 2 imm; Young–Fugate Cave; 14 Sep. 2016; T. Malabad leg.; • ♀; Young–Fugate Cave; 22 Oct. 2018; T. Malabad leg.; • ♂, 3♀; Young–Fugate Cave; 28 Oct. 2019; T. Malabad, K. Kosič Ficco leg.; • ♂, 3♀; Young–Fugate Cave, Fugate entrance; 24 Jun. 2020; T. Malabad, A. Malabad leg.; – **Virginia, Rockbridge Co.** • 3♂, 13♀; Dollhouse Cave, Natural Bridge, E of Springfield; 16 Sep. 1992; M. Hedin, S. O’Kane leg.; – **Virginia, Scott Co.** • 4♀; Alley Cave (entrance sink), E of Natural Tunnel State Park; 19 Sep. 1992; M. Hedin, S. O’Kane leg.; • 2♀; Alley Cave (entrance sink), E of Natural Tunnel State Park; 22 Aug. 2005; M. Hedin, R. Keith, J. Starrett, S. Thomas leg.; MCH 05_072; • ♀; Big Entrance Crawl Cave; 3 May. 2017; T. Malabad leg.; • ♂, 9♀; Cliff Mountain, Dry Branch, County Road 655, NE of Duffield; 36.7495°N, -82.7787°W; 7 Aug. 2004; M. Hedin, R. Keith, J. Starrett, S. Thomas leg.; MCH 04_028; • ♂, 2♀, 7 imm; Grisby Cave; 7 Mar. 2017; T. Malabad leg.; • ♂; Hill Cave, 5.2 miles northeast of Duffield, VA; 3 Mar. 2020; T. Malabad, K. Kosič Ficco, R. Blackwell, L. Young leg.; • ♂, 14♀, 2 imm; Hwy 23/58/421 at Moccasin Gap, near Weber City; 36.6338°N, -82.555°W; 22 Aug. 2005; M. Hedin, R. Keith, J. Starrett, S. Thomas leg.; MCH 05_073; • ♂, 2 imm; Kerns Cave; 16 Sep. 2015; W. Orndorff leg.; • 2♀, 1 imm; Kerns No. 1 Cave, northwest of Fort Blackmore, VA; 7 Mar. 2017; W. Orndorff leg.; • 4♀; Kerns No. 1 Cave; 4 Mar. 2020; T. Malabad, K. Kosic Ficco, R. Blackwell, L. Young leg.; • ♀; Spurlock Cave, northeast of Duffield, VA; 17 Dec. 2020; T. Malabad, K. Kosič Ficco, M. Ficco leg.; • 2♀; Summer Shaft, west of Dungannon, VA; 10 Sep. 2020; T. Malabad, K. Kosič Ficco, M. Ficco leg.; – **Virginia, Smyth Co.** • 3♂, 14♀; Atwell’s Tunnel Cave, N of Nebo; 17 Sep. 1992; M. Hedin, S. O’Kane leg.; • ♀; Beaver Creek Cave; 9 Dec. 2014; E. Koertge leg.; – **Virginia, Tazewell Co**. • ♀; Whitt Cave, southwest of Tazewell, VA; 6 May. 2021; T. Malabad, K. Kosič Ficco, M. Ficco leg.; – **Virginia, Wise Co.** • ♀; above Guest River, County Road 660, 3 mi. S of County Road 658, SE of Coeburn; 36.9009°N, -82.4146°W; 7 Aug. 2004; M. Hedin, R. Keith, J. Starrett, S. Thomas leg.; MCH 04_027; • ♀; Cloud Hole Cave, SW of East Stone Gap, VA; 18 Dec. 2020; T. Malabad, K. Kosič Ficco, M. Ficco leg.; • ♀; Getting Warmer Cave, NE of Big Stone Gap, VA; 24 May. 2020; T. Malabad, K. Kosič Ficco, M. Ficco, Sara Fleetwood, P. Schuchardt leg.; • ♀; Parsons Cave, southeast of East Stone Gap, VA; 29 Jan. 2020; T. Malabad, K. Kosič Ficco, R. Blackwell, Rick Reynolds, L. Young leg.; • ♀; Space Turtles Cave, NE of Big Stone Gap, VA; 13 Jun. 2020; T. Malabad, K. Kosič Ficco, M. Ficco, P. Schuchardt leg.; • ♀, 8 imm; Wildcat Caverns; 14 Sep. 2016; W. Orndorff leg.; – **West Virginia, Kanawha Co.** • 2♀; Kanawha SF, Davis Creek campground; 38.2474°N, -81.6586°W; 24 Jun. 2014; M. Hedin leg.; MCH 14_003; – **West Virginia, Mercer Co.** • 3♂, ♀, 1 imm; Camp Creek State Park, along Mash Fork; 37.5039°N, -81.1343°W; 4 Jun. 2016; M. Hedin, S. Derkarabetian, J. Starrett leg.; MCH 16_050; • ♂, 19♀; Camp Creek State Park, vic Campbell Falls trailhead; 37.5092°N, -81.1337°W; 15 Sep. 1992; M. Hedin, S. O’Kane leg.; • 4♂, 5♀; Camp Creek State Park, vicinity Blue Jay campground; 37.5137°N, -81.1309°W; 25 Jun. 2014; M. Hedin leg.; MCH 14_007; • 4♀; Camp Creek State Park, near campground; 37.5019°N, -81.1357°W; 13 Aug. 2007; M. Hedin, R. Keith leg.; MCH 07_095.

###### Diagnosis.

**Figure 50.**
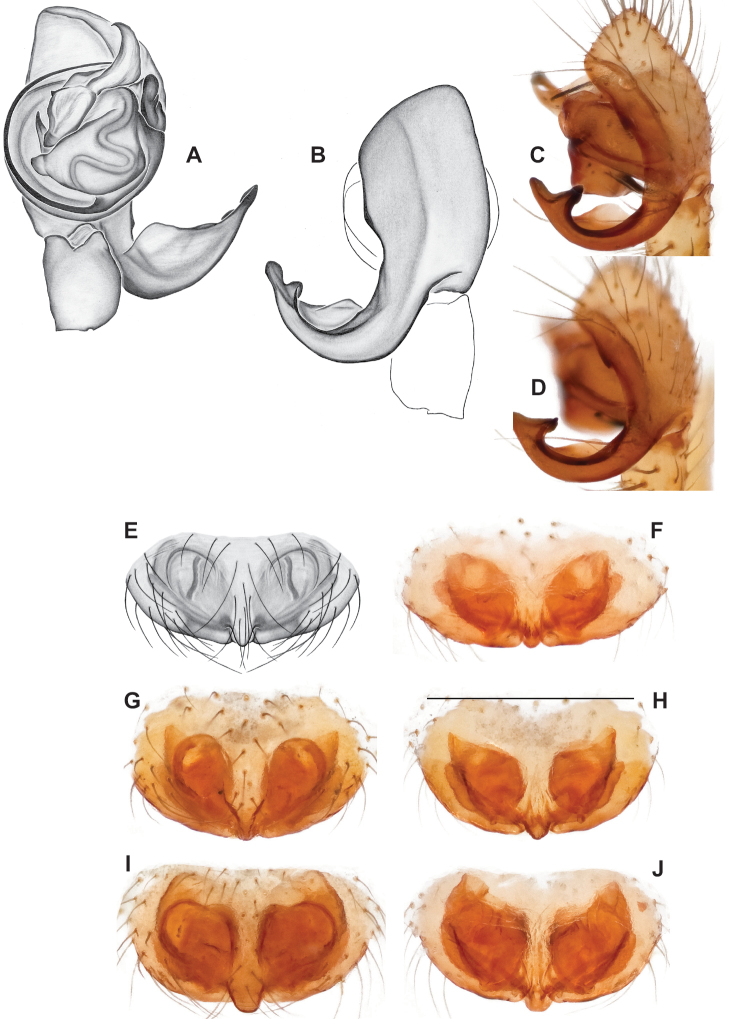
*Nesticuscarteri* ♂ palps. Tennessee, Hamilton Co., near Pitchfork Cave, MCH specimen #1571, ventral (**A**), dorsal (**B**). **C** Virginia, Scott Co., Cliff Mountain, MCH 04_028, dorsal **D** Tennessee, Hancock Co., S of Mulberry Gap, MCH 05_070, dorsal. *N.carteri* epigynal variation. Tennessee, Hamilton Co., near Pitchfork Cave, MCH specimen #1580, ventral (**E**), dorsal (**F**). Tennessee, Hancock Co., S of Mulberry Gap, MCH 05_070, ventral (**G**), dorsal (**H**). Virginia, Scott Co., Cliff Mountain, MCH 04_028, ventral (**I**), dorsal (**J**). Scale bar: 0.5 mm.

Male palp with a distinctive elongate conductor, with a tip that lacks the strong distal fold found in other Appalachian taxa. Strongly concave median apophysis with medial point, tegular apophysis with a shallow fork, basal branch just a small lobe (Fig. 50A–D). Paracymbium simple with a well-sclerotized, short paradistal process of various shape. Epigynum distinctive, wider than long with broad lateral pockets and an obviously pointed median septum (Fig. 50E–J).

###### Variation.

The paradistal dorsal process of the paracymbium varies in shape from very low and inconspicuous (Fig. 50A–D), to spoon-like, to rectangular (Gertsch fig. 124). The distal (highly sclerotized) fork of the tegular apophysis also varies in length and shape, from nearly straight to more curved. Males from the disjunct Pilot Mountain and Pitchfork Cave locations (Fig. 49) fall within this range of variation.

Minor variation was observed in the shape of the epigynal median septum (sometimes with a median bulge, then narrowing distally, viewed ventrally, Fig. 50E–J), but no obvious geographic trends were apparent.

###### Distribution and natural history.

This species has the largest known geographic distribution of any Appalachian *Nesticus* species, ranging from southern Tennessee (near Chattanooga) to southern Indiana, east to West Virginia, and southeast towards Winston-Salem (Fig. 49). Because we have collected this species from near surface habitats at relatively low elevations, we hypothesize that this taxon can withstand slightly drier situations, perhaps explaining this relatively broad distribution.

The southern Pitchfork Cave population is highly disjunct from all other more northerly records; this is possibly an artifact of insufficient collecting effort on the eastern edge of the Cumberland Plateau in east-central Tennessee (Fig. 49). UCE data indicate that the Pitchfork Cave population is genetically divergent (on a relatively long branch), and sister to all remaining *Nesticuscarteri* populations (Figs 3, 4).

This species is known from both caves (both deeper and twilight situations) and dark, relatively moist near-surface habitats (mostly void spaces in rock piles). As noted above, *Nesticuscarteri* has been collected in near syntopy with *N.holsingeri* at Alley Cave, Virginia, where the former is found in a talus sink leading to the cave entrance, the latter collected from the dark zone of the cave.

###### Remarks.

This species is not recovered as monophyletic on mitochondrial gene trees but is instead fragmented into three separate clades (Fig. 6).

##### ﻿ Nesticus georgia

Taxon classificationAnimaliaAraneaeNesticidae

Gertsch, 1984

A9344A70-202B-5918-8ADE-99CC5FFAAA85

Fig. 51A–C

Nesticus georgia Gertsch, 1984: 39, figs 156–158, 164–166.

###### Material examined.

**Type material: *Holotype***: USA – **Georgia, Dade Co.** • ♂ holotype; Sitton’s Cave, near Trenton; 28 Nov. 1952, E.J. Kuenzler leg.; AMNH. **New collections from type locality**: – **Dade Co.** • ♂, ♀; Sitton’s Cave, 1 mi E of Trenton; 30 Sep. 1991; M. Hedin, K. Crandall leg.; • 2♂, 3♀; Sitton’s Cave; 23 Sep. 1992; M. Hedin, S. O’Kane leg.

###### Diagnosis.

**Figure 51.**
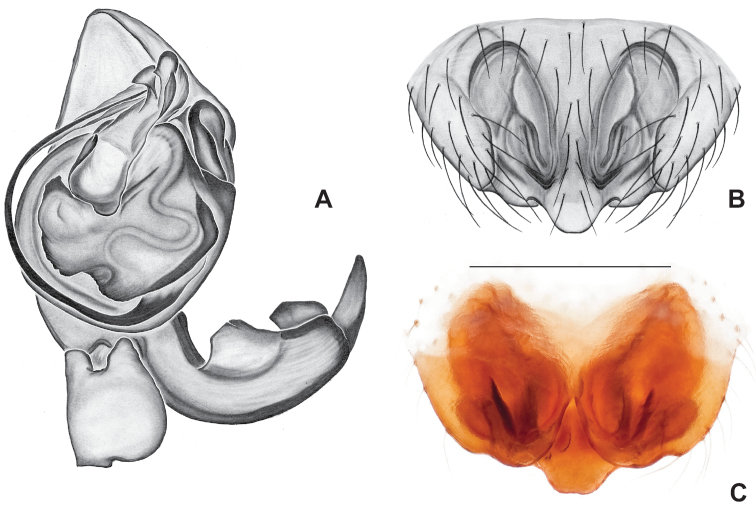
*Nesticusgeorgia* genitalia **A** Georgia, Dade Co., Sitton’s Cave, MCH specimen #1015, ♂ palp, ventral. Georgia, Dade Co., Sitton’s Cave, MCH specimen #1014, epigynum, ventral (**B**), dorsal (**C**). Scale bar: 0.5 mm.

Nearly eyeless, long-legged taxon. Male palp most similar to that of *Nesticuslula*, but with a spatulate tegular apophysis and details of the distal edge of the paracymbial ventral process with a sclerotized process projecting dorsally (Fig. 51A). Ventral epigynum very close to similarly troglomorphic *N.lula*, difficult to separate based on epigynal morphology alone (compare Fig. 51B, C to Zigler and Milne 2022: figs 2, 3). Both *N.georgia* and *N.lula* are also similar in overall epigynal morphology to the more distantly related (but geographically proximate) *N.barri* (Fig. 48C).

###### Distribution and natural history.

Known only from a handful of limestone caves from three adjacent counties in northwest Georgia (Fig. 49; Hedin and Dellinger 2005: fig. 1; Carver et al. 2016: fig. 2). Reeves (1999) summarized natural history information (microhabitat preference, fecundity, prey items, etc.) for topotypic *Nesticusgeorgia*.

##### ﻿ Nesticus lula

Taxon classificationAnimaliaAraneaeNesticidae

Zigler & Milne, 2022

5A41123C-F230-5744-BE2F-27EB2EDEC423

Fig. 49

Nesticus lula Zigler & Milne, 2022: 293, figs 1A, C, 2, 3, 7.

###### Material examined.

**Type material: *Holotype***: USA – **Georgia, Walker Co.** • ♂; Lula Falls Cave (GWK617); 15 Apr. 2014; K.S. Zigler, L. Carver, L. Lyles leg.; KSZ 13–169 (SDSU_G2084). **Non type material**: – **Walker Co.** • ♂; Bee Rock Cave (GWK123); 31 May. 2015; K.S. Zigler, T. Lichtefeld, M. Abercrombie leg.; KSZ 15–388.

###### Diagnosis.

Morphological diagnosis as in Zigler and Milne (2022).

###### Distribution and natural history.

This troglomorphic taxon is currently known from only two caves in northwestern Georgia (Fig. 49; Zigler and Milne 2022: fig. 7).

#### ﻿*reclusus* group, including:

*Nesticussheari* Gertsch, 1984

*Nesticusdellingeri* sp. nov.

*Nesticusjonesi* Gertsch, 1984

*Nesticusbinfordae* sp. nov.

*Nesticusdykemanae* sp. nov.

*Nesticusbishopi* Gertsch, 1984

*Nesticusstupkai* Gertsch, 1984

*Nesticusreclusus* Gertsch, 1984

Phylogenomic structure indicates three subclades within this larger group, including a distinctive *Nesticussheari* sister to all other species, and a close-knit *N.dellingeri* subgroup sister to a *N.reclusus* subgroup. This overall structure is strongly supported by both concatenated and coalescent UCE analyses (Figs 3, 4). Relationships within the *N.reclusus* subgroup are particularly challenging, where concatenated versus coalescent UCE analyses differ in resolution, and both differ from morphology and mitochondrial evidence.

The mitochondrial data do not support the overall *reclusus* group as monophyletic, and although several species are recovered as monophyletic, their interrelationships vary strongly from that suggested by nuclear data (Fig. 6). Also, some species supported by nuclear evidence are intermixed on mitochondrial trees, suggesting a role for mitochondrial introgression. These patterns are more fully discussed in the species accounts below.

**Figure 52.**
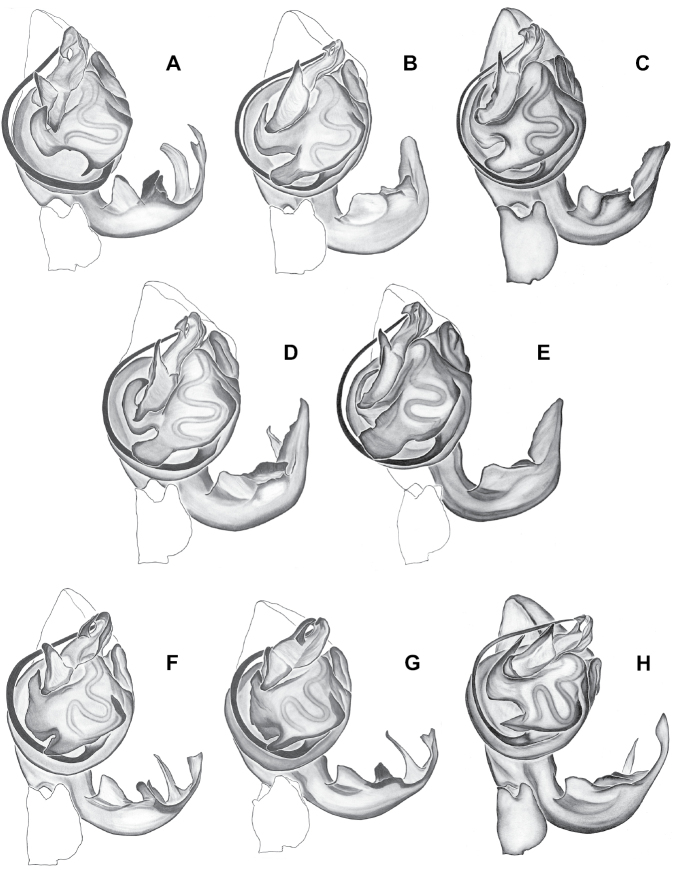
Comparative ♂ morphology of *reclusus* group species **A***Nesticussheari***B***N.dellingeri***C***N.jonesi***D***N.binfordae***E***N.dykemanae***F***N.bishopi***G***N.stupkai***H***N.reclusus*. All views ventral. See subsequent figures for specimen locations and voucher details.

Male genital morphology suggests common ancestry for this complex of eight species (Fig. 52). The male tegular apophysis is approximately S-shaped (with modifications), and the ventral paracymbial process includes an associated ventromedial process that varies in shape (although this is mostly lacking in *Nesticusdykemanae*). In our discussion of this group below we refer to a male paracymbial paradistal process; it is possible that this represents a distally migrated dorsal process (Fig. 52).

**Figure 53.**
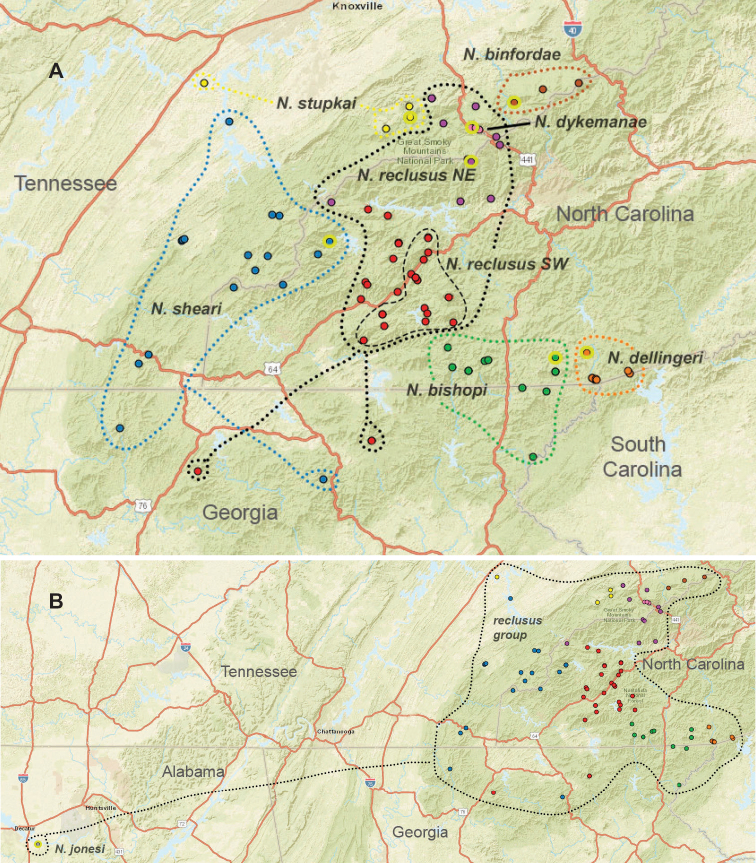
**A** distribution of *reclusus* group species **B** distribution of *Nesticusjonesi*. Type localities designated with yellow circles. State boundaries and major cities shown for geographic context. Dashed lines circumscribe known species distributions. The geographic distribution of “*N.cooperi*-like” populations is circled; this includes some female-only locations which are inside this distribution and included here only for purposes of graphical convenience.

Species in this group are distributed in the montane southern Blue Ridge west of the Asheville Basin, except for the geographically disjunct *Nesticusjonesi* known from a single cave in northern Alabama (Fig. 53). Groups of taxa show interesting parallel geographic distributions in the southern Blue Ridge. This includes a southeastern *N.dellingeri* sister to taxa from the Great Smoky Mountains, a southern *N.bishopi* related to *N.stupkai* also from the Great Smoky Mountains, and *N.reclusus* which also spans from south to north (Fig. 53).

##### ﻿ Nesticus sheari

Taxon classificationAnimaliaAraneaeNesticidae

Gertsch, 1984

124263E1-7955-570D-AA5A-39DD3F29CF70

Fig. 54A–F

Nesticus sheari Gertsch, 1984: 32, figs 79–81, 135–137.

###### Material examined.

**Type material: *Holotype***: USA – **North Carolina, Graham Co.**• ♂ holotype; Joyce Kilmer Memorial Forest, Poplar Cove; 30 May 1975; W.A. Shear leg.; AMNH; **New collections from type locality**: – **North Carolina, Graham Co.**• 3♂, 7♀; Joyce Kilmer Memorial Forest, NW of Robbinsville; 35.3585°N, -83.9291°W; 1 Aug. 1992; M. Hedin leg.; • 4♀; Joyce Kilmer Memorial Forest, NW of Robbinsville; 35.3585°N, -83.9291°W; 28 Aug. 2002; M. Hedin, M. Lowder, P. Paquin leg.; MCH 02_164; • 2♀; Joyce Kilmer Memorial Forest, NW of Robbinsville; 35.3585°N, -83.9291°W; 17 Aug. 2007; M. Hedin, M. McCormack, S. Derkarabetian leg.; MCH 07_115; **Non type material.** – **Georgia, Fannin Co.** • 19♀; Cohutta Wilderness, Cowpen Trail, NW Three Forks Mountain trailhead; 34.8905°N, -84.5715°W; 22 Aug. 2002; M. Hedin, M. Lowder, P. Paquin leg.; MCH 02_151; **Georgia, Union Co.** • 4♀; Sosebee Cove State Natural Area, off Hwy 180, 2 mi W jnct Hwy 19, S Blairsville; 34.7617°N, -83.9482°W; 18 Apr. 1994; M. Hedin leg.; – **North Carolina, Cherokee Co.** • 4♀; along Tipton Creek, 1.2 mi. SNC/TN stateline; 35.2503°N, -84.0724°W; 26 Aug. 2002; M. Hedin, M. Lowder, P. Paquin leg.; MCH 02_158; • 2♀; FR 50 Shuler Creek, below Wolf Ridge; 35.2424°N, -84.2227°W; 17 Aug. 2004; M. Hedin, R. Keith, J. Starrett, S. Thomas leg.; MCH 04_054; – **North Carolina, Graham Co.** • ♂, 2♀; along Wright Creek, S Santeetlah Creek, W of Seven Springs Gap; 35.3258°N, -83.9647°W; 19 Aug. 1991; B. Dellinger, D. Loch leg.; – **Tennessee, Loudon Co.** • ♂, ♀; Blankenship Cave, TLN1; 25 Jan. 2014; M.L. Niemiller, E.T. Carter leg.; MLN 14–006; – **Tennessee, Monroe Co.** • ♂, 2♀, 2 imm; Alans Hideaway Cave, TMO9; 16 Nov. 2013; M.L. Niemiller, E.T. Carter, M. Finkle leg.; MLN 13–079.1; • 2♀; along North River, FR 217, Unicoi Mountains; 35.3215°N, -84.1199°W; 17 Aug. 2007; M. Hedin, M. McCormack, S. Derkarabetian leg.; MCH 07_112; • ♂, 16♀, 4 imm; along Tellico River, near Bald River Falls; 35.3248°N, -84.1787°W; 26 Aug. 2002; M. Hedin, M. Lowder, P. Paquin leg.; MCH 02_157; • 7♀; Bald River, FR 126, E of Holly Flats campground; 35.2855°N, -84.1586°W; 17 Aug. 2007; M. Hedin, M. McCormack, S. Derkarabetian leg.; MCH 07_113; • 2♂, 8♀; Citico Creek near confluence with Flat Creek; 35.4252°N, -84.1047°W; 27 Aug. 2002; M. Hedin, M. Lowder, P. Paquin leg.; MCH 02_160; • ♀; Doublecamp Creek, 0.5 mi. E confluence with Citico Creek, Unicoi Mountains; 35.4224°N, -84.0847°W; 17 Aug. 2007; M. Hedin, M. McCormack, S. Derkarabetian leg.; MCH 07_114; • ♀, 4 imm; Gay Cave, TMO3; 16 Nov. 2013; ML Niemiller, C.D.R. Stephen, M. Finkle leg.; MLN 13–077; • 3♀, 5 imm; Lick Creek Cave, TMO8; 16 nov. 2013; M.L. Niemiller, C.D.R. Stephen, E.T. Carter, M. Finkle leg.; MLN 13–078; – **Tennessee, Polk Co.** • ♂, 10♀; FR 221, N of Peavine Mountain, vicinity Big Frog Mountain Wilderness; 35.0531°N, -84.5139°W; 23 Aug. 2002; M. Hedin, M. Lowder, P. Paquin leg.; MCH 02_152; • ♂; S Ocoee River at Thunder Rock Road, off Hwy 64; 35.0743°N, -84.4852°W; 17 Aug. 2004; M. Hedin, R. Keith, J. Starrett, S. Thomas leg.; MCH 04_052.

###### Diagnosis.

**Figure 54.**
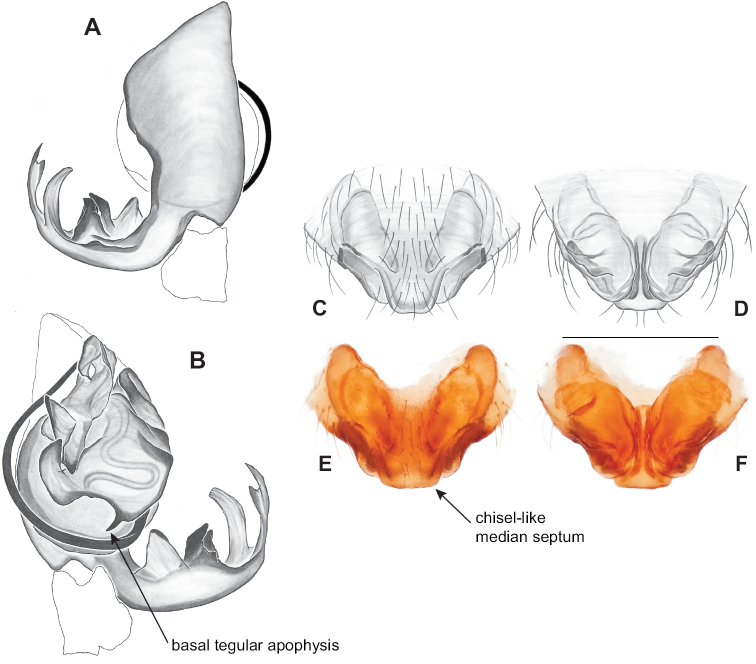
*Nesticussheari* genitalia. Tennessee, Polk Co., N of Peavine Mountain, vicinity Big Frog Mountain Wilderness, MCH 02_152, ♂ palp, dorsal (**A**), ventral (**B**). Tennessee, Polk Co., vicinity Big Frog Mountain Wilderness, MCH 02_152, epigynum, ventral (**C**), dorsal (**D**). Georgia, Union Co., Sosebee Cove State Natural Area, MCH specimen #1995 epigynum, ventral (**E**), dorsal (**F**). Scale bar: 0.5 mm.

The diagnosis of Gertsch (1984) is here modified to recognize relationships to other *Nesticusreclusus* group members. Male paracymbium with the combination of translucent blade-like paradistal process, distal process with twisted, tubular tip; well-sclerotized, toothlike dorsomedial process adjacent to small flange-like ventromedial process (Fig. 54A, B). These paracymbium characters are similar to those found in *N.bishopi* and *N.stupkai*. Male tegular apophysis with a general S-curve, distal process a truncate curving blade, basal process nipple-like. Acute distal median apophysis. In females the posterior end of the epigynal median septum is “squared-off” on three sides, like a chisel (Fig. 54C–F), projecting inwards towards the abdomen.

###### Variation.

In males from non-type locations the ventromedial process is more confluent with the ventral process (less displaced medially) and more elongate.

Different populations exhibit very little epigynal variation despite a large and fragmented geographic distribution. Some adult females from Holly Flats campground are approximately one-half the size of other adult females.

###### Distribution and natural history.

Previously known only from the type locality at Joyce Kilmer Memorial Forest, which now represents one of the easternmost known records for the species. The species distribution almost forms a circle in the montane uplands that surround the Ducktown lowlands (lacking the eastern edge), with disjunct Cohutta, Sosebee Cove, and Blankenship Cave populations (Fig. 53). It appears to surround the geographic distribution of the more narrowly distributed *Nesticusbondi*. Previously thought to be a strictly montane taxon, but we report here several important new cave records.

As an example of natural history we include here field notes for Cohutta Wilderness (MCH 02_151) where we collected 19♀. Notes read “small drainage in pine forest, rocks in drainage, moist but not running water”, where spiders were collected from beneath rocks.

As discussed above *Nesticussheari* (4♀) was found in sympatry with *N.bondi* (♂, 6♀) at Tipton Creek. Because we did not identify specimens directly in the field, it remains unclear if these different species were found side-by-side or were perhaps somehow segregated by microhabitat. *Nesticussheari* was also collected in sympatry with the nesticid *Eidmanella* Roewer, 1935 at Doublecamp Creek (07_114); *Nesticus* is otherwise rarely found in sympatry with members of this genus.

###### Remarks.

Monophyletic on mitochondrial and nuclear trees, with high gene and site CF values for the latter. Phylogenomic evidence strongly supports *Nesticussheari* as sister to remaining members of the *reclusus* group (Figs 3, 4).

##### ﻿ Nesticus dellingeri sp. nov.

Taxon classificationAnimaliaAraneaeNesticidae

D6741DA3-4B20-5759-8822-83426FBAB4DE

https://zoobank.org/BDC1A285-4B27-4549-901B-FB2BBDE32B64

Fig. 55A–G

###### Material examined.

**Type material: *Holotype***: USA – **North Carolina, Macon Co.** • holotype ♂; vicinity Whiteside Mountain, off Hwy 64, SW of Cashiers; 35.0793°N, -83.1415°W; 8 Aug. 1992; M. Hedin, I.-M. Tso leg.; MCH specimen #1047; ***Paratypes***: – **North Carolina, Macon Co.** • 5♂, 9♀; vic Whiteside Mountain, off Hwy 64, SW of Cashiers; 35.0793°N, -83.1415°W; 8 Aug. 1992; M. Hedin, I.–M. Tso leg.; **Non type material**: – **North Carolina, Jackson Co.** • 2♀; along Chattooga River, NE side near mouth Scotsman Creek; 35.013°N, -83.1123°W; 16 Aug. 1991; B. Dellinger leg.; • ♂; along Chattooga River, NE side, 0.2 mi. W mouth Scotsman Creek; 35.0136°N, -83.1135°W; 17 Aug. 1991; B. Dellinger leg.; • ♂, 5♀; Whitewater River, below Upper Falls; 35.0337°N, -83.0141°W; 2 Sep. 2002; M. Hedin, F. Coyle, M. Lowder, P. Paquin leg.; MCH 02_183; – **North Carolina, Macon Co.** • ♂, 5♀, 5 imm; Chattooga River, vic BullPen bridge crossing; 35.0172°N, -83.1262°W; 2 Sep. 2002; M. Hedin, M. Lowder, P. Paquin leg.; MCH 02_180; • 7♀; Chattooga River, vic BullPen bridge crossing, SE of Highlands; 35.0172°N, -83.1262°W; 8 Aug. 1992; M. Hedin leg.; – **South Carolina, Oconee Co.** • ♂, 3♀; along Whitewater River, just S NC/SC stateline; 35.0271°N, -83.0094°W; 13 Apr. 1992; B. Dellinger leg.

###### Diagnosis.

Sister to other members of a phylogenomic subclade including *Nesticusbinfordae*, *N.dykemanae* and *N.jonesi*, and morphologically most similar to these geographically disjunct taxa (in particular, sharing the spade-like basal tegular apophysis; Fig. 52). Males of *N.dellingeri* differ from males of these other taxa in the shape of the distal tegular apophysis (broad vs. skinny), the shape of the median apophysis, the shape of the paracymbial distal process, and in details of the paracymbial ventral process cusps. This species shares a very similar epigynal morphology with other members of the subclade (see descriptions below).

###### Description of ♂ holotype

**Figure 55.**
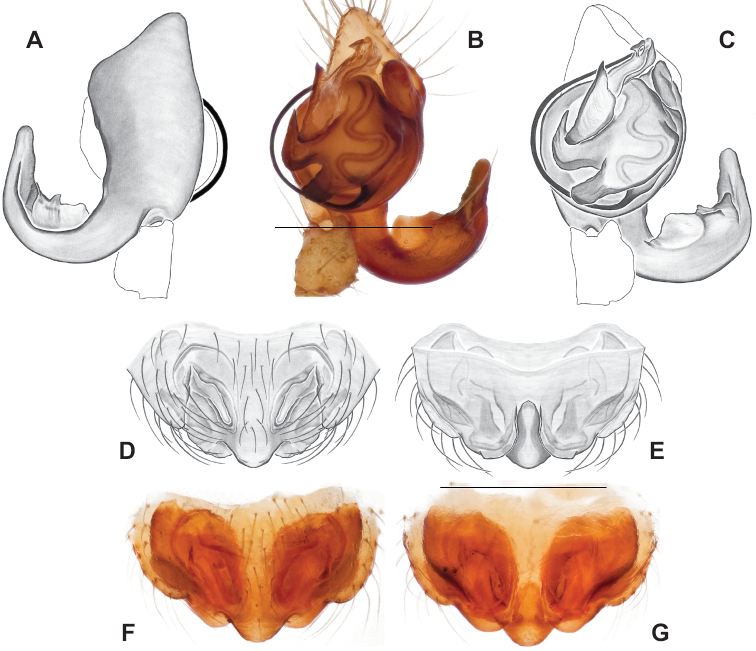
*Nesticusdellingeri* sp. nov. genitalia. North Carolina, Macon Co., vicinity Whiteside Mountain, MCH specimen #1047, ♂ palp, dorsal (**A**), ventral (**B**) **C** North Carolina, Jackson Co., Whitewater River, below Upper Falls, MCH 02_183, palp, ventral. North Carolina, Macon Co., vicinity Whiteside Mountain, MCH specimen #1057, epigynum, ventral (**D**), dorsal (**E**). North Carolina, Jackson Co., Whitewater River, below Upper Falls, MCH 02_183, ventral (**F**), dorsal (**G**). Scale bar: 0.5 mm.

**(MCH specimen #1047).** Carapace dusky cream to orange, with faint dark pigment behind ocular area and along carapace margin. Legs approximately concolorous pale. Abdomen with paired, lateral darker markings on dirty gray background. All eyes approximately equal in size, AMEs barely visible. Eyes with light rings of dark pigment. CL 1.4, CW 1.1, abdomen length 2, total body length 3.4. Leg I total length 9.9 (2.75, 0.6, 3, 2.5, 1.05), leg formula 1423, leg I / CW ratio 9.0. Palp with broadly S-shaped tegular apophysis, distal part a short, curved blade with acute tip, basal fork of apophysis a short, square sclerotized spade (Fig. 55A–C). Median apophysis anvil-shaped, distal end a sharp tip. Conductor tip bent, surrounded by small funnel-shaped cuticular sheath. Paracymbium lacking a paradistal process, distal process finger-like with slight serration along paradistal edge. Lacking a dorsomedial process. Distal part of ventral paracymbial process thickened, with small cusps.

###### ♂ Variation.

Males from four non-type localities match topotypic males very closely (Fig. 55A–C).

###### Description of ♀ paratype

**(MCH specimen #1057).** Carapace color as in male, slightly darker orange. Legs approximately concolorous pale. Abdomen with paired, lateral darker maculations on dirty gray background. All eyes approximately equal in size, AMEs miniscule but visible. Eyes with rings of dark pigment. CL 1.2, CW 1.1, abdomen length 1.65, total body length 2.85. Leg I total 7.3 (2.1, 0.55, 2.05, 1.7, 0.9), leg formula 1423, leg I / CW ratio 6.6. Epigynum generally wider than tall, median septum relatively wide with directly adjacent lateral pockets (Fig. 55D–G). Posterior end of septum with lateral bars oriented obliquely upwards, dark spermathecae lying beneath these bars and approximately following the upwards oblique path. Median septum narrowing past these bars and projecting inwards towards the abdomen. Viewed dorsally, dorsal internal pockets lying slightly above sclerotization of the lateral pockets.

###### ♀ Variation.

Females from different locations share a very similar epigynal morphology (Fig. 55D–G).

###### Distribution and natural history.

Known only from a very small area in the upper Chattooga River and upper Whitewater River drainages (Fig. 53), along the south face of the Blue Ridge Escarpment in the North and South Carolina borderlands. Except for the type locality, most collections are relatively small in total animals collected, suggesting a natural rarity for this species. At the type locality (Whiteside Mountain) a total of 14 adults and 8 immature specimens was collected from a crevice cave at the base of rocky cliffs.

*Nesticusbishopi* has also been collected from nearby locations in the Chattooga River Gorge (locations near Scotsman Creek; Fig. 53), suggesting that these species might somewhere be syntopic in this area.

###### Etymology.

This species is named to recognize and honor Bob Dellinger, a special naturalist from western North Carolina. Bob’s knowledge of the flora and fauna of southern Appalachia is remarkable, and he personally collected or helped to collect (with first author MH) many *Nesticus* from this region.

###### Remarks.

*Nesticusdellingeri* is geographically disjunct from phylogenetic relatives *N.binfordae*, *N.dykemanae* and *N.jonesi* (Fig. 53). The regions separating these taxa have been extensively sampled for *Nesticus* and are occupied by species from other species groups (*N.nasicus*, *tennesseensis* group members), or more distant *reclusus* group members.

##### ﻿ Nesticus jonesi

Taxon classificationAnimaliaAraneaeNesticidae

Gertsch, 1984

73464578-AB0C-5E50-9827-99690C857C6F

Fig. 56A–C

Nesticus jonesi Gertsch, 1984: 38, figs 153–155, 167–169.

###### Material examined.

**Type material: *Holotype***: USA – **Alabama, Morgan Co.** • ♂ holotype; Cave Spring Cave; 2 May 1959; W.B. Jones, Royer, Steeves, T.C. Barr leg; AMNH; **New collections from type locality**: – **Morgan Co.** • 4♂, 14♀; Wheeler NWR, Cave Spring Cave, E of Decatur; 14 Nov. 1992; M. Hedin, J. Hedin leg.

###### Diagnosis.

**Figure 56.**
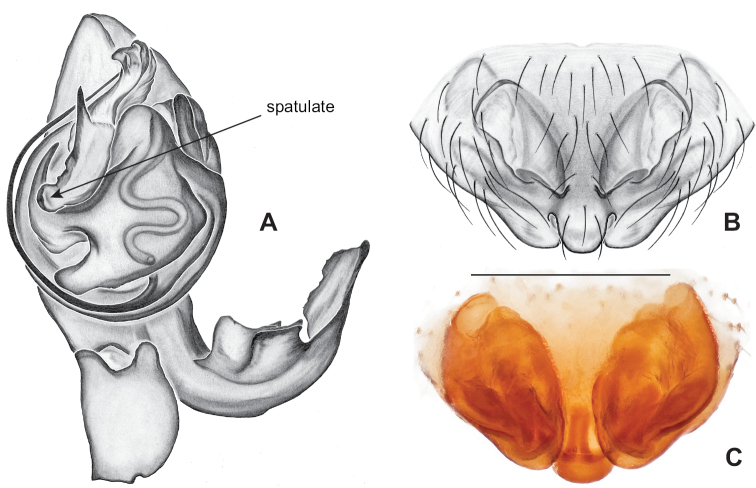
*Nesticusjonesi* genitalia **A** Alabama, Morgan Co., Cave Spring Cave, MCH specimen #1644, ♂ palp, ventral. Alabama, Morgan Co., Cave Spring Cave, MCH specimen #1656, epigynum, ventral (**B**), dorsal (**C**). Scale bar: 0.5 mm.

Similar to regional congener *Nesticusbarri*, this species is long-legged and nearly eyeless, but is otherwise morphologically and genetically allied with members of the *reclusus* group from montane western North Carolina. Very similar in male and female genital morphology to close phylogenomic kin *N.dellingeri* (Fig. 55A–G), *N.dykemanae* (Fig. 59A–C) and *N.binfordae* (57A–C), but geographically disjunct, troglomorphic, and larger in body size. Also differing from these taxa in the shape of the tegular apophyses (both basal and distal), and the shape of the basal edge of the median apophysis (Fig. 56A).

###### Distribution and natural history.

This species is known only from the type locality south of the Tennessee River in north-central Alabama (Fig. 53). Geographically far-flung from phylogenetic relatives, perhaps similar to the biogeographic situation observed in *Nesticuspaynei* and/or *N.carteri*, both of which also include disjunct populations towards the southern end of the Tennessee River valley.

Collections in 1992 revealed a very large spider population in Cave Spring Cave, perhaps up to 1,000 individuals. This cave is home to a protected bat colony and located in a US National Wildlife Refuge. The extraordinary size of the *Nesticusjonesi* population is perhaps related to the high productivity associated with the large bat colony and/or the protected status of this cave.

###### Remarks.

Part of a near phylogenomic trichotomy with *Nesticusdykemanae* and *N.binfordae* (Figs 3, 4), with sCF values near a lower limit.

##### ﻿ Nesticus binfordae sp. nov.

Taxon classificationAnimaliaAraneaeNesticidae

6918A538-2103-5156-A9EC-58BFE6F41345

https://zoobank.org/70333742-4E8D-46B5-BA00-455CEEAA4E32

Figs 57A–C , 58A–F

###### Material examined.

**Type material: *Holotype***: USA – **Tennessee, Sevier Co.** • ♂ holotype; Great Smoky Mountains NP, Greenbrier Cove, Middle Prong Little Pigeon River, 1.3 mi. upstream Greenbrier Picnic Area; 35.7042°N, -83.3653°W; 20 Aug. 1992; M. Hedin leg; MCH specimen #1290; ***Paratypes***: – **Sevier Co.** • ♀ paratype; data as for holotype; MCH specimen #1287; • 5♂, 14♀; data as for holotype; **Non type material**: – **Cocke Co.** • ♀; Great Smoky Mountains NP, N side Indian Camp Creek on Maddron Bald Trail; 35.7378°N, -83.2777°W; 16 Apr. 1994; M. Hedin, B. Dellinger leg.; • 2♀; Great Smoky Mountains NP, trail from Low Gap to Mt. Cammerer; 35.754°N, -83.1658°W; 1 Aug. 2000; M. Hedin leg.; MCH 00_146.

###### Diagnosis.

Most similar to close phylogenomic kin *Nesticusdykemanae* (Fig. 59A–C) and *N.jonesi* (Fig. 56A). Differing from the latter in having a sharp-tipped median apophysis, the shape of the basal tegular apophysis, and having a whip-like paradistal paracymbial process. Very similar to *N.dykemanae*, sharing the double-tipped median apophysis, but differing in the shape of the basal tegular apophysis and possessing a whip-like paradistal process. Sharing an almost identical epigynal morphology with *N.jonesi*.

###### Description of ♂ holotype

**Figure 57.**
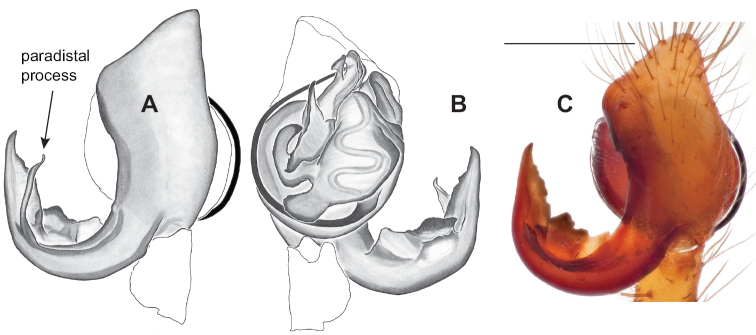
*Nesticusbinfordae* sp. nov. ♂ palps. Tennessee, Sevier Co., Great Smoky Mountains NP, Middle Prong Little Pigeon River, MCH specimen #1290, dorsal (**A**), ventral (**B**). Middle Prong Little Pigeon River, MCH specimen #1289, dorsal (**C**). Scale bar: 0.5 mm.

**(MCH specimen #1290).** Carapace dirty light orange, dusky lines leading from fovea to eye group. Legs colored as carapace, without markings. Abdomen background color as carapace, six pairs of lateral faint darker markings. All eyes approximately equal in size, except for AMEs, ~ 1/4 width of ALEs. Eyes with rings of dark pigment. CL 1.5, CW 1.3, abdomen length 1.7, total body length 3.2. Leg I total length 14.2 (3.9, 0.7, 4.35, 3.85, 1.4), leg formula 1423, leg I / CW ratio 10.9. Palp with broadly S-shaped tegular apophysis, distal part a short skinny curved blade with tapered tip, basal fork of apophysis a squat sclerotized spade with rounded edges (Fig. 57A–C). Median apophysis anvil-shaped, both ends with sharp tips. Conductor tip bent, surrounded by small funnel-shaped cuticular sheath. Paracymbium with a skinny whip-like paradistal process, and distal process finger-like with slight serration along paradistal edge. Lacking a dorsomedial process. Distal part of ventral paracymbial process thickened, forming a small blade without cusps (Fig. 57A–C).

###### ♂ Variation.

Males are only known from the type locality and all match the holotype male, except for MCH specimen #1289 which lacks the paracymbial paradistal process (Fig. 57C). Close examination of this specimen suggests that this process was broken off (process base is evident).

###### Description of ♀ paratype

**(MCH specimen #1287).** Carapace subdued burnt orange, distinct darker markings leading from fovea forward, dusky ring to edge of carapace. Legs light orange, with faint dusky dark markings. Abdomen slightly paler than carapace, with fused distal lateral dark markings. Posterior eyes approximately equal in size, ALE slightly smaller than PLEs, AMEs ~ 1/4 width of ALEs. Eyes with rings of dark pigment. CL 1.65, CW 1.4, abdomen length 2.35, total body length 4. Leg I total length 13.15 (3.75, 0.75, 4, 3.25, 1.4), leg formula 1423, leg I / CW ratio 9.4. Epigynum generally wider than tall, median septum relatively wide at top with adjacent heart-shaped lateral pockets (considering both sides). Septum narrows towards posterior end where lateral bars extend obliquely upwards, dark spermathecae lying beneath these bars and approximately following the upwards oblique path. Median septum extending past these bars and dipping inwards towards the abdomen. Viewed dorsally, dorsal internal plates lying slightly above sclerotization of the lateral pockets.

###### ♀ Variation.

**Figure 58.**
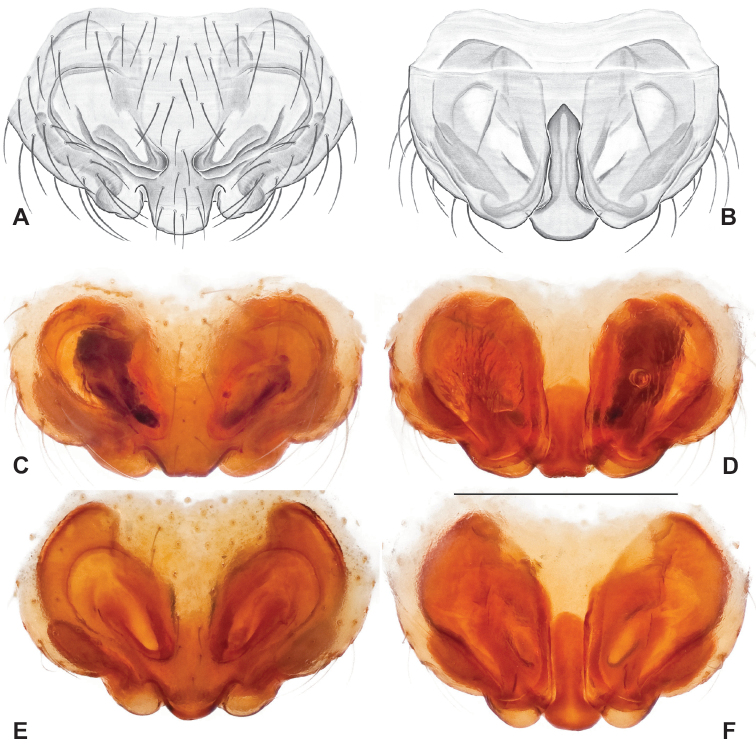
*Nesticusbinfordae* sp. nov. epigynal variation. Tennessee, Sevier Co., Great Smoky Mountains NP, Middle Prong Little Pigeon River, MCH specimen #1283, ventral (**A**), dorsal (**B**). Tennessee, Cocke Co., Great Smoky Mountains NP, N side of Indian Camp Creek, MCH specimen #1981, ventral (**C**), dorsal (**D**). Tennessee, Cocke Co., Great Smoky Mountains NP, trail from Low Gap to Mt. Cammerer, MCH 00_146, ventral (**E**), dorsal (**F**). Scale bar: 0.5 mm.

Females from different locations share a very similar epigynal morphology (Fig. 58A–F).

###### Distribution and natural history.

Known only from three parallel north-flowing drainages in the Great Smoky Mountains National Park, including the Middle Prong of the Little Pigeon River, and more easterly draining Indian Camp and Cosby Creeks.

At the type locality in 1992 spiders were “very abundant in rock crevices, low to the ground, close to the river”.

Along the Maddron Bald and Mt. Cammerer trails we collected both *Nesticuscherokeensis* and *N.binfordae*, indicating that these species are syntopic or nearly so at these locations. At both locations multiple collections were taken along an elevational transect and unfortunately lumped into a single collecting event, so it is not possible to discern if different species were collected at the exact same location (truly syntopic) or were closely parapatric along these elevational transects.

###### Etymology.

Named to honor Dr. Greta Binford. Friend, arachnologist, and Past President of the American Arachnological Society (AAS), here recognized for her inspirational spider research and her leadership in making the AAS a more diverse and welcoming society. We suspect that Dr. Binford would also greatly appreciate the beauty of the habitats that this spider calls home.

###### Remarks.

Part of a near phylogenomic trichotomy with *Nesticusdykemanae* and *N.jonesi* (Figs 3, 4), with sCF values near a lower limit.

This species was called “N novsp2” (from site 48) in Hedin (1997b) and lumped with *Nesticusdykemanae* despite having distinctive (non-sister) ND1/16S sequences.

##### ﻿ Nesticus dykemanae sp. nov.

Taxon classificationAnimaliaAraneaeNesticidae

89571AB7-DD90-5683-8621-0C91D8FC255E

https://zoobank.org/6A313CD5-EA60-4F68-8CBF-37887618A8B1

Fig. 59A–I

###### Material examined.

**Type material: *Holotype***: USA – **Tennessee, Sevier Co.** • ♂ holotype; Great Smoky Mountains NP, Hwy 441 near Chimney Tops trailhead; 35.6364°N, -83.4709°W; 31 Jul. 2000; M. Hedin, J. Cokendolpher leg.; MCH 00_143 (SDSU_TAC000673); **Type material: *Paratypes***: – **Sevier Co.** • ♂, 4♀; Great Smoky Mountains NP, Hwy 441 near Chimney Tops trailhead; 35.6364°N, -83.4709°W; 31 Jul. 2000; M. Hedin, J. Cokendolpher leg.; MCH 00_143; **Non type material: – Sevier Co.** • ♂, 8♀; Great Smoky Mountains NP, Hwy 441 half way between tunnel and Chimney picnic area; 35.6414°N, -83.4819°W; 16 Apr. 1994; M. Hedin, F. Coyle, B. Dellinger leg.; • 5♀; Great Smoky Mountains NP, Hwy 441 N Chimneys campground; 35.6406°N, -83.4949°W; 27 Aug. 2005; M. Hedin, R. Keith, J. Starrett, S. Thomas leg.; MCH 05_098.

###### Diagnosis.

**Figure 59.**
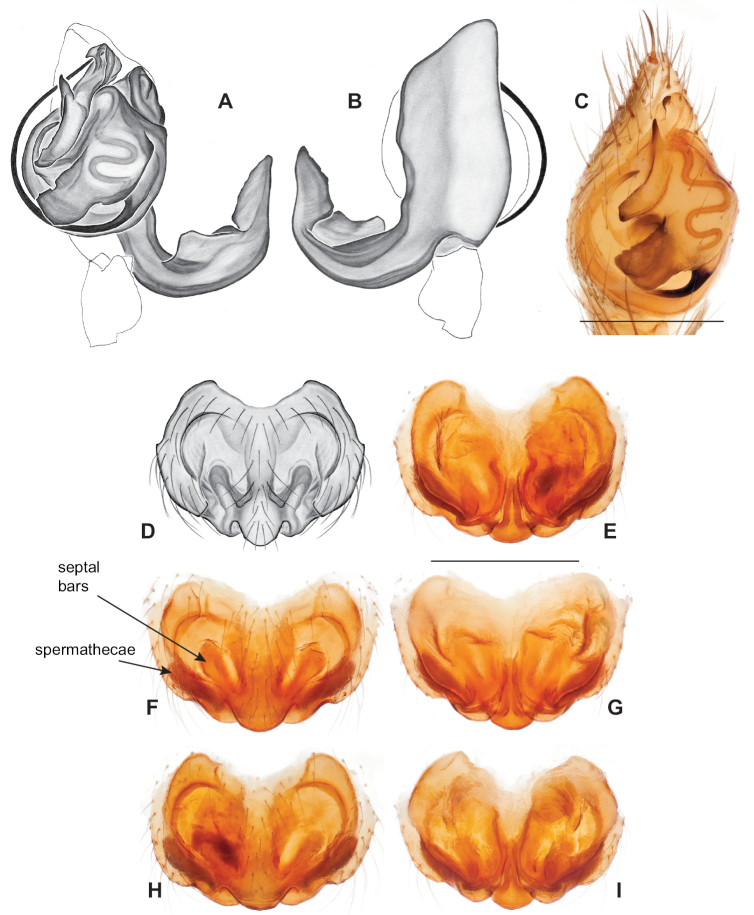
*Nesticusdykemanae* sp. nov. genitalia. ♂ palps – Tennessee, Sevier Co., Great Smoky Mountains NP, Hwy 441 near Chimney Tops trailhead, MCH 00_143 (SDSU_TAC000673), ♂ palp ventral (**A**), dorsal (**B**) **C** Tennessee, Sevier Co., Great Smoky Mountains NP, Hwy 441, between tunnel and Chimney picnic area, MCH specimen #1977, ♂ palp ventral. Scale bar: 0.5 mm. epigynal variation – Tennessee, Sevier Co., Great Smoky Mountains NP, Hwy 441 near Chimney Tops trailhead, MCH 00_143 (SDSU_TAC000674), epigynum ventral (**D**), dorsal (**E**). Tennessee, Sevier Co., Great Smoky Mountains NP, Hwy 441, between tunnel and Chimney picnic area, MCH specimen #1973, epigynum ventral (**F**), dorsal (**G**). Tennessee, Sevier Co., Great Smoky Mountains NP, Hwy 441, N of Chimneys campground, epigynum ventral (**H**), dorsal (**I**). Scale bar: 0.5 mm.

This species is included in a phylogenomic subclade with *Nesticusjonesi* and *N.binfordae*. Males share the double-tipped median apophysis with the latter species but differ in the shape of both distal and proximal tegular apophyses, lack a whip-like paracymbial paradistal process, and have a less modified ventral paracymbial process (Fig. 59A–C). The epigynum is most distinctive in the larger phylogenomic subclade (including *N.dellingeri*), with lateral bars that extend from the median septum obliquely upwards at approximately 45-degree angles, interrupting the lateral pockets (Fig. 59D–I). Also, dark spermathecae lie below the septum bars, extending obliquely outwards.

###### Description of ♂ holotype

**(SDSU_TAC000673).** Carapace dusky cream to orange, with conspicuous faint dark pigment behind ocular area, and along carapace margin bleeding inwards. Legs approximately concolorous pale. Abdomen with strong paired, lateral darker markings on a dirty orange/gray background. All eyes approximately equal in size, except for AMEs, ~ 1/4 width of ALEs. Eyes with rings of dark pigment. CL 1.5, CW 1.3, abdomen length 1.5, total body length 3. Leg I total length 14.25 (3.95, 0.75, 4.35, 3.8, 1.4), leg formula 1423, leg I / CW ratio 11.0. Palp with broadly S-shaped tegular apophysis, distal part a particularly skinny curved blade with sharp tip, basal fork of apophysis a squat sclerotized spade with saw-like leading edge (Fig. 59A–C). Median apophysis anvil-shaped, both ends with sharp tips, apical end more tongue-shaped. Conductor tip bent, surrounded by small funnel-shaped cuticular sheath. Paracymbium lacking paradistal process, distal process finger-like with slight serration along paradistal edge. Lacking a dorsomedial process. Distal part of ventral paracymbial process only slightly thickened, lobe-like (Fig. 59A–C).

###### ♂ Variation.

Other than the holotype male only two other males are known, and these closely match the holotype. MCH specimen #1977 (Fig. 59C) appears slightly unusual because this male had recently molted.

###### Description of ♀ paratype

**(SDSU_TAC000674).** Carapace color as in male, dark pigment not as strong. Legs approximately concolorous pale, very faint pigmentation. Abdomen with strong paired, lateral darker markings on a slightly lighter background. Eye development as in male. Eyes with rings of dark pigment. CL 1.45, CW 1.25, abdomen length 1.95, total body length 3.4. Leg I total length 11.3 (3.25, 0.6, 3.35, 2.9, 1.2), leg formula 1423, leg I / CW ratio 9.0. Epigynum generally wider than tall, median septum with adjacent heart–shaped lateral pockets (considering both sides). Septum towards posterior end with lateral bars that extend obliquely upwards at approximately 45-degree angles, interrupting lateral pockets. Dark spermathecae lying below septum bars, extending obliquely outwards. Median septum broadening slightly past these bars and dipping inwards towards the abdomen. Viewed dorsally, dorsal internal plates lying distinctly above the sclerotized ring of the lateral pockets.

###### ♀ Variation.

Females from adjacent locations share a very similar epigynal morphology (Fig. 59D–I).

###### Distribution and natural history.

Known from three closely adjacent locations from near the headwaters of the West Prong of the Little Pigeon River, Great Smoky Mountains National Park, on the southwest slopes of Mt. Leconte (Fig. 53). All nearby surrounding collections have resulted in the collection of *Nesticusreclusus* (Fig. 53), suggesting that this microendemicity is real (rather than a collecting artifact). Gertsch (1984) also includes records of *N.reclusus* from the “top of Mt. Leconte”.

1994 collections from near the Chimney Picnic Area resulting in collections of a male and eight females were from a “large talus breakdown in a south-facing cove” in rich hardwood forest.

###### Etymology.

Named to honor Wilma Dykeman (1920–2006), a writer, speaker, teacher, historian, and environmentalist who spent most of her life in western North Carolina and eastern Tennessee. Mrs. Dykeman was devoted to social justice and environmental integrity, discussing Appalachian water pollution in her classic 1955 book ‘The French Broad’, and sharing a social justice award in 1957 for her co-authored book ‘Neither Black Nor White’.

###### Remarks.

Part of a near phylogenomic trichotomy with *Nesticusbinfordae* and *N.jonesi* (Figs 3, 4), with sCF values near a lower limit.

This species was called “*N novsp2*” (from site 49) in Hedin (1997b) and lumped with *N.binfordae* despite having distinctive (non-sister) ND1/16S sequences.

##### ﻿ Nesticus bishopi

Taxon classificationAnimaliaAraneaeNesticidae

Gertsch, 1984

AC17DABF-EC31-5EBD-ACA0-9977D03496CA

Figs 60A–D , 61A–H

Nesticus bishopi Gertsch, 1984: 33, figs 147–149.

###### Material examined.

**Type material: *Holotype***: USA – **North Carolina, Macon Co.** • ♀ holotype; Highlands; 6 Apr. 1929; S.C. Bishop leg.; AMNH; **New collections from near type locality**: – **North Carolina, Macon Co.** • ♂, 8♀; below Glenn Falls, SW of Highlands; 35.0312°N, -83.2383°W; 2 Aug. 1992; M. Hedin leg.; • ♂, ♀; below Glenn Falls, SW of Highlands; 35.0312°N, -83.2383°W; 31 Aug. 2002; M. Hedin, M. Lowder, P. Paquin leg.; MCH 02_175; **Non type material**: – **Georgia, Rabun Co.** • 2♀; Holcomb Branch of Holcomb Creek, off Hale Ridge Road, NE of Rabun Bald; 34.9831°N, -83.2661°W; 14 Apr. 1992; T. McGarity leg.; • 2♂, 3♀; Hwy 246/106 along Mud Creek, NE of Dillard; 34.9924°N, -83.3385°W; 19 Aug. 2007; M. Hedin, M. McCormack, S. Derkarabetian leg.; MCH 07_126; • 2♂, ♀; Hwy 76 at Chattooga River crossing, confluence with Pole Creek; 34.8172°N, -83.3061°W; 2 Sep. 2002; M. Hedin, F. Coyle, M. Lowder, P. Paquin leg.; MCH 02_181; – **North Carolina, Jackson Co.** • ♀; along Chattooga River, NE side between Scotsman and Glade Creek; 35.0123°N, -83.1164°W; 13 Jul. 1992; B. Dellinger leg.; – **North Carolina, Macon Co.** • ♂, 8♀; 4.3 mile S Standing Indian campground, along Nantahala River; 35.0347°N, -83.5057°W; 10 Aug. 1992; M. Hedin leg.; • ♀, 5 imm; 4.3 mile S Standing Indian campground, along Nantahala River; 35.0347°N, -83.5057°W; 20 Aug. 2002; M. Hedin, F. Coyle, M. Lowder, P. Paquin leg.; MCH 02_140; • 2♂, 5♀, 2 imm; along Black Creek, NE side Chunky Gal Mountain; 35.092°N, -83.5663°W; 20 Aug. 2002; M. Hedin, F. Coyle, M. Lowder, P. Paquin leg.; MCH 02_141; • 2♀; Coweeta Hydrological Lab, along Shope Fork of Coweeta Creek, FR 751; 35.0611°N, -83.4447°W; 19 Aug. 2007; M. Hedin, M. McCormack, S. Derkarabetian leg.; MCH 07_125; • ♂; Coweeta Hydrological Lab, along Shope Fork of Coweeta Creek, W of Otto; 35.0601°N, -83.4547°W; 23 Oct. 2012; M. Hedin, J. Bond leg.; MCH 12_043; • 2♀; FR 710, 2 mi. N Deep Gap; 35.0425°N, -83.555°W; 20 Aug. 2002; M. Hedin, F. Coyle, M. Lowder, P. Paquin leg.; MCH 02_142; • 9♀; near Dry Falls, Cullasaja River, off Hwy 64 NW of Highlands; 35.069°N, -83.239°W; 11 Aug. 1992; M. Hedin leg.

###### Diagnosis.

**Figure 60.**
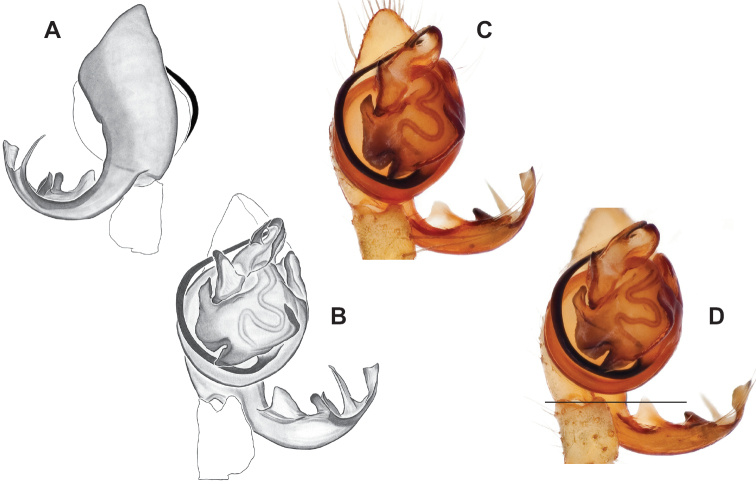
*Nesticusbishopi* ♂ palps. North Carolina, Macon Co., below Glenn Falls, MCH specimen #1078, dorsal (**A**), ventral (**B**) **C** Georgia, Rabun Co., Chattooga River at confluence with Pole Creek, MCH 02_181, ventral **D** North Carolina, Macon Co., along Black Creek, MCH 02_141, ventral. Scale bar: 0.5 mm.

**Figure 61.**
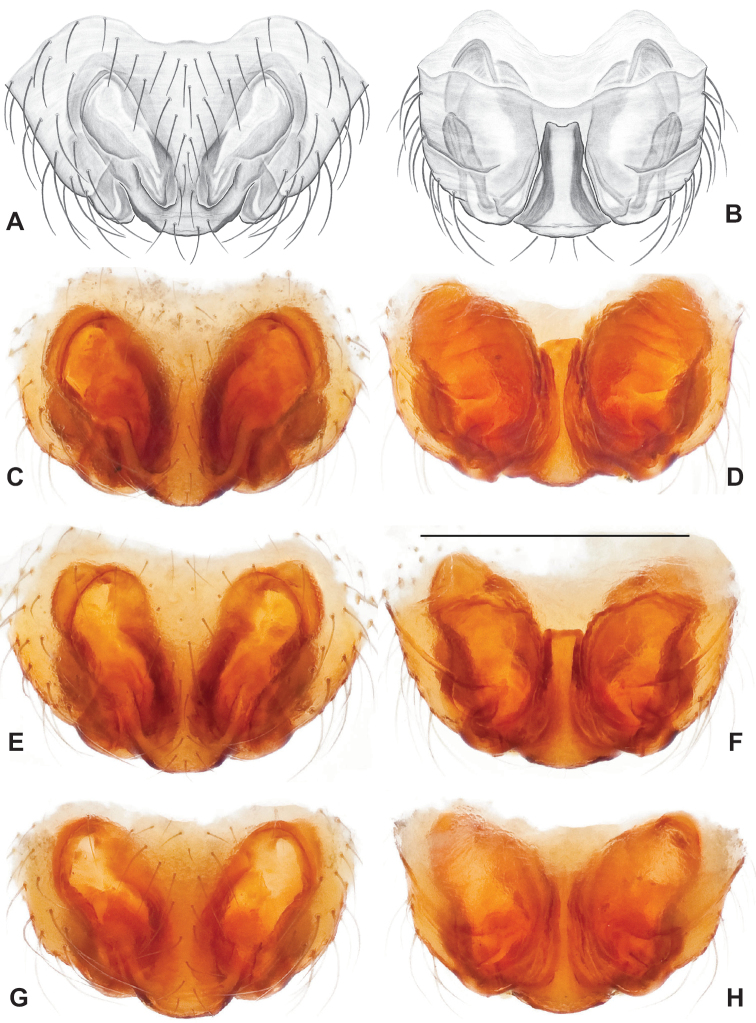
*Nesticusbishopi* epigynal variation. North Carolina, Macon Co., below Glenn Falls, MCH specimen #1071, ventral (**A**), dorsal (**B**). Georgia, Rabun Co., Chattooga River at confluence with Pole Creek, MCH 02_181, ventral (**C**), dorsal (**D**). North Carolina, Macon Co., along Black Creek, MCH 02_141, ventral (**E**), dorsal (**F**). North Carolina, Jackson Co., along Chattooga River, between Scotsman and Glade Creek, MCH specimen #2016, ventral (**G**), dorsal (**H**). Scale bar: 0.5 mm.

**Figure 62.**
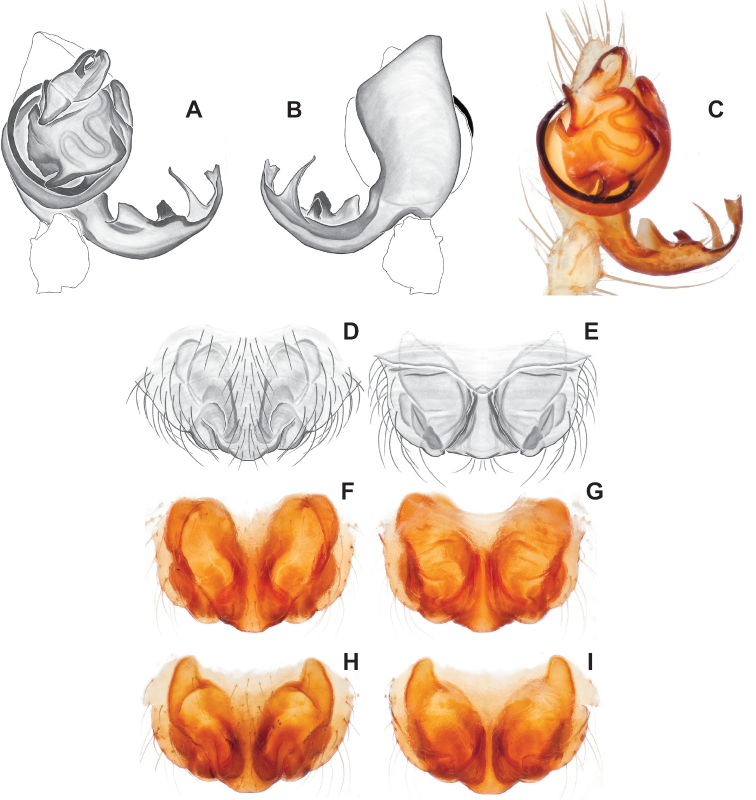
*Nesticusstupkai* genitalia. Tennessee, Blount Co., Blowing Cave, NE of Townsend, MCH specimen #1555, ♂ palp, ventral (**A**), dorsal (**B**) **C** Tennessee, Blount Co., Great Smoky Mountains NP, White Oak Sinks, holotype ♂ palp, ventral. Tennessee, Blount Co., Blowing Cave, MCH specimen #1567, epigynum, ventral (**D**), dorsal (**E**). Tennessee, Blount Co., Great Smoky Mountains NP, Little River at Mile 40 of Hwy 73, MCH specimen #1305, epigynum, ventral (**F**), dorsal (**G**). Tennessee, Blount Co., Great Smoky Mountains NP, White Oak Sinks, MCH specimen #1304, epigynum, ventral (**H**), dorsal (**I**). Scale bar: 0.5 mm.

**Figure 63.**
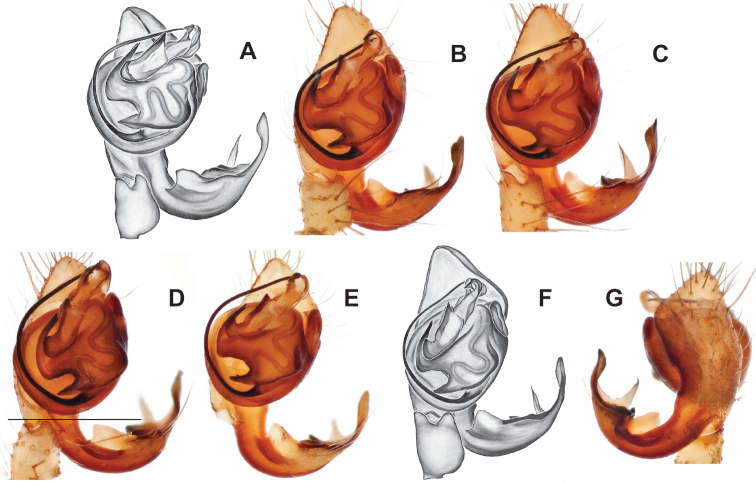
northeastern *Nesticusreclusus* ♂ palps, ventral view (*except for **G**) **A** Tennessee, Sevier Co., Wear Cove, Myhr Cave, MCH 01_182 (*right palp, inverted in Photoshop) **B** Tennessee, Sevier Co., Great Smoky Mountains NP, Lower Baskins Creek **C** Tennessee, Sevier Co., Great Smoky Mountains NP, Hwy 441, N of Newfound Gap **D** Tennessee, Sevier Co., Great Smoky Mountains NP, Elkmont Area, MCH 00_144 **E** North Carolina, Swain Co., Great Smoky Mountains NP, south of Clingman’s Dome, near Forney Ridge parking area **F** North Carolina, Swain Co., Great Smoky Mountains National Park, Andrew’s Bald (male holotype, AMNH) **G** North Carolina, Swain Co., Cheoah Dam along Cheoah Reservoir (MCH 07_116), dorsal view. Scale bar: 0.5 mm.

**Figure 64.**
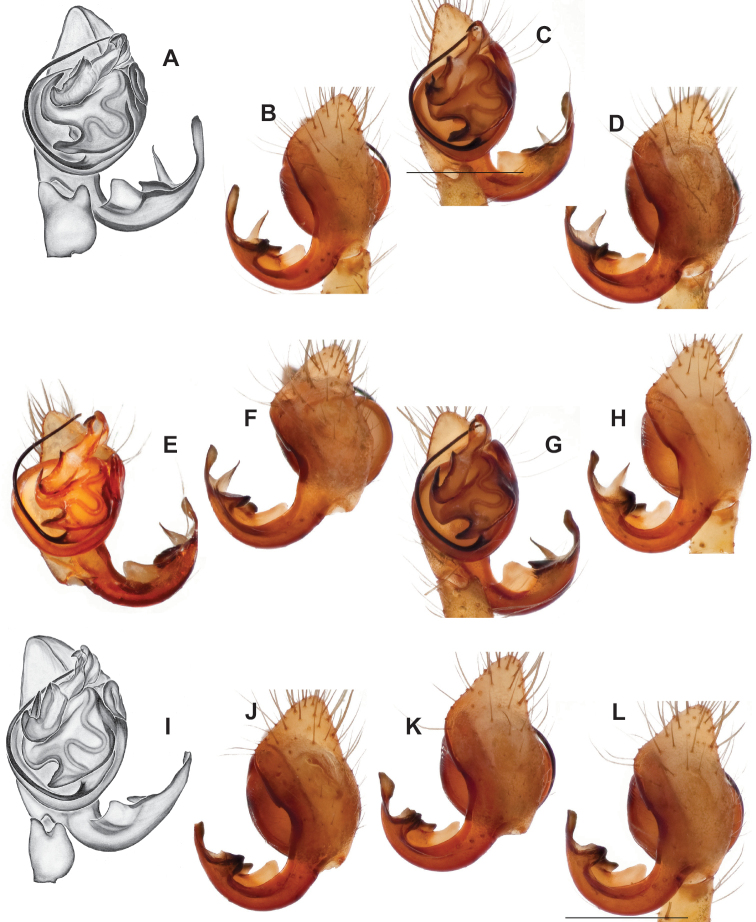
southwestern *Nesticusreclusus* ♂ palps. North Carolina, Graham Co., Appalachian Trail, S of Stecoah Gap, MCH 02_165, ventral (**A**), dorsal (**B**) North Carolina, Graham Co., ENE of Fontana Village, MCH 07_120, ventral (**C**), dorsal (**D**). Georgia, Gilmer Co., Rock Creek Road, N of Rich Mountain Wilderness, MCH 07_102, ventral (**E**), dorsal (**F**). North Carolina, Cherokee Co., Beaver Creek Road, MCH 04_057, ventral (**G**). North Carolina, Cherokee Co., S of Tatham Gap, MCH 04_058, dorsal (**H**). North Carolina, Clay Co., Tusquitee Mountains, Long Branch of Fires Creek, MCH 02_144, ventral (**I**), dorsal (**J**). North Carolina, Swain Co., Nantahala River Gorge, Nantahala River Gorge, across from Talc Mountain quarry, dorsal (**K**). North Carolina, Macon Co., S Wayah Bald, MCH 02_169, dorsal (**L**). Scale bar: 0.5 mm.

Compared to other members of the challenging *reclusus* subgroup, *Nesticusbishopi* is similar in detail in all aspects of male and female morphology to *N.stupkai* (compare Figs 60A–D, 61A–H, 62A–H). Shared male features include a distal tegular apophysis shoe-shaped with a beak-like tip, base of distal part with a sclerotized and blade-like shoulder, proximal fork of tegular apophysis arrowhead-like, median apophysis short and triangular, paracymbium with translucent bladelike paradistal process, distal process with twisted tip, and toothlike distomedial process directly adjacent to small flange-like ventromedial process. Although we cannot find morphological characters that distinguish *N.bishopi* from *N.stupkai*, we retain both as distinct based on diagnostic DNA characters and an allopatric distribution (see further arguments in the Discussion). Both species are easily distinguished from closely-related *N.reclusus* by the shape of the male median and tegular apophyses (Figs 63A–F, 64A–F).

###### Description of ♂ from near type locality

**(MCH specimen #1078).** Carapace dusky cream to orange, conspicuous faint dark pigment behind ocular area. Legs pale yellow to cream. Abdomen dirty pale cream, faint paired lateral pigmentation blotches. Eyes approximately equal in size, except for AMEs, ~ 1/3 width of ALEs. Eyes with rings of dark pigment. CL 1.3, CW 1.1, abdomen length 1.75, total body length 3.05. Leg I total length 9.75 (2.65, 0.55, 2.95, 2.5, 1.1), leg formula 1423, leg I / CW ratio 8.9. Palp with broadly S-shaped tegular apophysis, distal part shoe-shaped with a beak-like tip, base of distal part with a sclerotized and blade-like shoulder. Basal fork of tegular apophysis like a sclerotized broad-based arrowhead (Fig. 60A–D). Median apophysis short and triangular. Conductor tip bent, surrounded by small funnel-shaped cuticular sheath. Paracymbium with translucent bladelike paradistal process, distal process with twisted, tubular tip; well-sclerotized, toothlike distomedial process directly adjacent to small flange-like ventromedial process (Fig. 60A–D).

###### Variation.

Males and females from both sides of the Little Tennessee River barrier (see below) share very similar genitalic morphologies (Figs 60A–D, 61A–H).

###### Distribution and natural history.

From montane habitats in southern North Carolina and northern Georgia. Populations are found both east (Cowee Mountains, including the type locality) and west (Nantahala Mountains) of the Little Tennessee River, a known dispersal barrier in other arachnid taxa (e.g., Thomas and Hedin 2008; Keith and Hedin 2012; Hedin and McCormack 2017). Western populations (Black Creek Road, Standing Indian, Coweeta, Deep Gap Road) indeed form a subclade on mitochondrial trees (Fig. 6).

As an example of natural history, 1992 collections near Standing Indian Campground were made in a northwest-facing rocky ravine, where many specimens were collected “in dark ravine, wet, deep litter, rocks, *Rhododendron*”.

*Nesticusbishopi* has been collected from locations very near *N.dellingeri* in the Chattooga River gorge (locations near Scotsman Creek; Fig. 53), suggesting that these species might somewhere be syntopic in this area.

###### Remarks.

The species is obviously morphologically very similar to a disjunct *Nesticusstupkai* and is arguably conspecific from a morphological perspective. We have retained *N.bishopi* as distinct at the species level because this taxon is monophyletic on both UCE and mitochondrial trees (Figs 3, 4, 6), and is geographically disjunct from sister species *N.stupkai*. One complication is that *N.stupkai* is paraphyletic with respect to *N.bishopi* on nuclear trees (Figs 3, 4), as further discussed below.

We have made extensive collections of other *Nesticus* taxa in the region that separates *N.bishopi* from *N.stupkai*, finding only other *Nesticus* species (e.g., *N.silvanus*, *N.cherokeensis*, etc.; Fig. 53). As such, we view the probability of on-going gene flow as low.

##### ﻿ Nesticus stupkai

Taxon classificationAnimaliaAraneaeNesticidae

Gertsch, 1984

003F02A4-CD81-5C15-A6E9-C578CB406311

Fig. 62A–I

Nesticus stupkai Gertsch, 1984: 31, figs 71–74, 106–108; Reeves 2000: 338.

###### Material examined.

**Type material: *Holotype***: USA – Tennessee, **Blount Co.** • ♂ holotype; White Oak Sinks, Great Smoky Mountains National Park; 21 Jul. 1937; A. Stupka leg; AMNH; **New collections from near type locality**: – **Blount Co.** • ♀; Great Smoky Mountains NP, White Oak Sinks, Blowhole; 21 Aug. 1992; M. Hedin leg. **Non type material**: – **Blount Co.** • 6♂, 9♀; Blowing Cave, NE Townsend, off Hwy 321; 22 Sep. 1992; M. Hedin, S. O’Kane leg.; • ♀; Great Smoky Mountains NP, Little River at Mile 40 of Hwy 73; 35.6688°N, -83.6827°W; 22 Aug. 1992; M. Hedin leg.; – **Loudon Co.** • ♂, 4♀, 16 imm; Benjos Cave, TLN11; 30 Aug. 2014; M.L. Niemiller, C.D.R. Stephen, E.T. Carter leg.; MLN 14–044.17.

###### Diagnosis.

See Diagnosis of *Nesticusbishopi* for details on shared male morphology. Females are likewise similar to *N.bishopi*, with a narrowing median septum with posterior bars that form an anchor shape, directed upwards and outwards, spermathecae lying lateral to these bars at approximately the same angle (compare Fig. 61A–H. to Fig. 62D–I). Without knowledge of geographic origin, we cannot distinguish epigynal morphologies of these two species.

**Figure 65.**
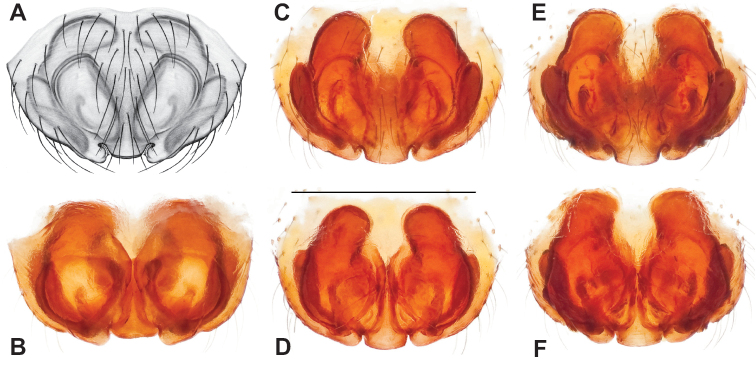
northeastern *Nesticusreclusus* epigynal variation. North Carolina, Swain Co., Great Smoky Mountains NP, south of Clingman’s Dome, vicinity Forney Ridge parking area, MCH specimen #1973, ventral (**A**), dorsal (**B**). North Carolina, Swain Co., Great Smoky Mountains NP, N of Smokemont Campground turnoff, MCH specimen #N1019, ventral (**C**), dorsal (**D**). North Carolina, Swain Co., Great Smoky Mountains NP, Noland Creek at Laurel Branch, MCH specimen #N1051, ventral (**E**), dorsal (**F**). Scale bar: 0.5 mm.

Females of *Nesticusstupkai* and *N.bishopi* can be distinguished from the closely related *N.reclusus* by the outwards oriented dorsal epigynal plates in the former (Figs 61A–H, 62D–I), vs. the inwards oriented dorsal epigynal plates in the latter (Fig. 65A–F).

###### Variation.

Males and females from different populations share very similar genitalic morphologies (Fig. 62A–C).

###### Distribution and natural history.

With a distribution similar to *Nesticusbarrowsi*, from cave entrances in karst windows along the northwestern edge of Great Smoky Mountains National Park (Fig. 53), and nearby surface (boulderfield) habitats. We include a new important western record from Benjos Cave, southwest of Knoxville in the Tennessee River Valley.

As an example of natural history, specimens from Blowing Cave were collected from a cave entrance, while those from Little River were collected from beneath rockpiles directly adjacent to a stream.

Reeves (2000) reported *Nesticusstupkai* in sympatry with *N.barrowsi* at two cave locations in Great Smoky Mountains National Park, and we collected this taxon pair in near sympatry in the White Oak Sinks, with *N.barrowsi* found in the dark zone of caves, and *N.stupkai* found closer to cave entrances (twilight zone). Reeves (2000) also recorded *N.stupkai* from Myhr Cave. We have confirmed male *N.reclusus* from this location (Fig. 63A). This is either a case of sympatry or an original misidentification, as females of these species can be difficult to distinguish.

###### Remarks.

*Nesticusbishopi* plus *N.stupkai* together form a strongly supported nuclear clade (Figs 3, 4). Within this clade however *N.stupkai* is not monophyletic on UCE trees, with the White Oak Sinks (type) population strongly supported as more closely related to a geographically distant *N.bishopi* clade than to the geographically adjacent Little River plus Blowing Cave *N.stupkai* clade (Figs 3, 4).

One possibility is that the latter clade (Little River, Blowing Cave) is not *Nesticusstupkai*, but a separate lineage. We have closely compared males and females from the White Oak Sinks (type) population to males and females from Blowing Cave and detect no morphological differences (Fig. 62A–C). Despite paraphyly, and as an expectation to the species criteria used in this revision, we retain *N.stupkai* as a distinct species.

##### ﻿ Nesticus reclusus

Taxon classificationAnimaliaAraneaeNesticidae

Gertsch, 1984

E7FFDFD6-708E-5564-8033-33A8644B285A

**Figure 66.**
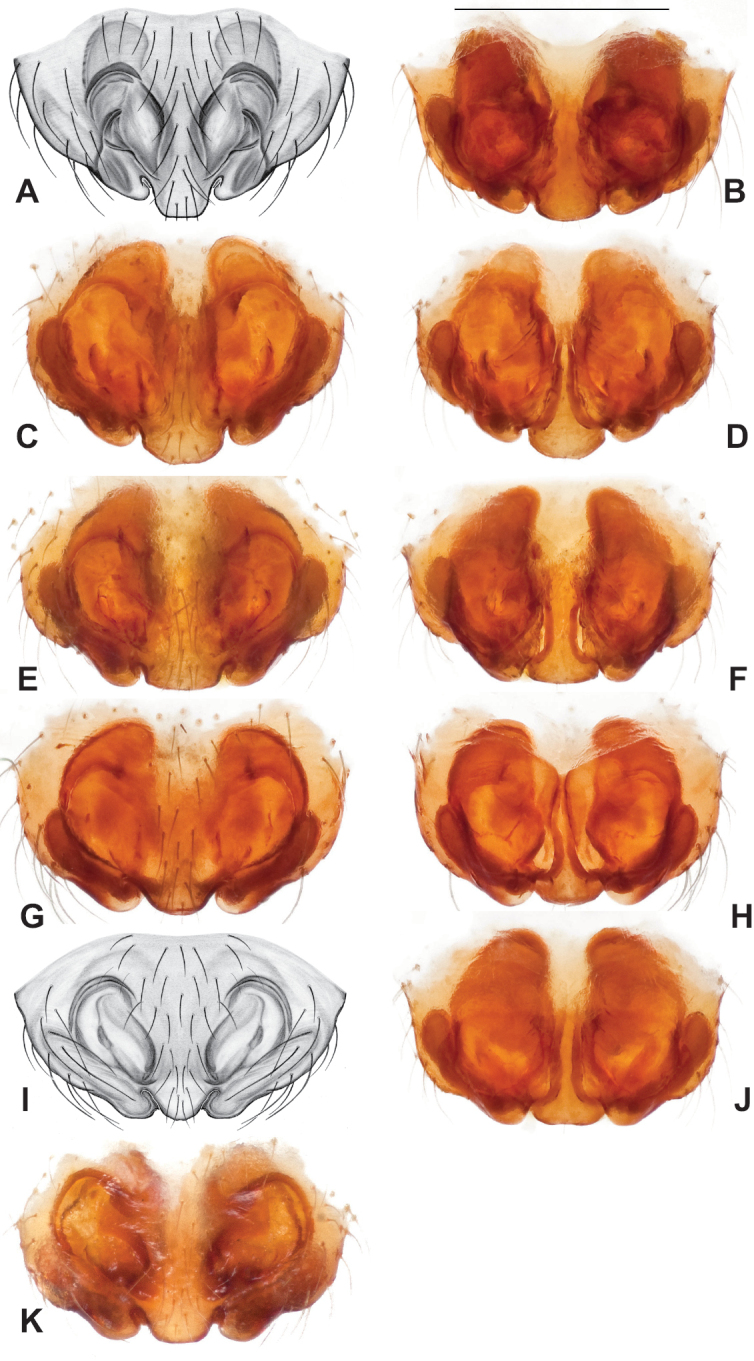
southwestern *Nesticusreclusus* epigynal variation. North Carolina, Graham Co., Appalachian Trail, S of Stecoah Gap, MCH 02_165, ventral (**A**), dorsal (**B**). North Carolina, Graham Co., ENE of Fontana Village, MCH 07_120, ventral (**C**), dorsal (**D**). North Carolina, Cherokee Co., Beaver Creek Road, MCH 04_057, ventral (**E**), dorsal (**F**). Georgia, Gilmer Co., Rock Creek Road, N of Rich Mountain Wilderness, MCH 07_102, MCH specimen #N1160, ventral (**G**), dorsal (**H**). North Carolina, Clay Co., Tusquitee Mountains, Long Branch of Fires Creek, MCH 02_144, ventral (**I**), dorsal (**J**). North Carolina, Macon Co., Ball Road, SE of Beechertown, MCH 04_072, ventral (**K**). Scale bar: 0.5 mm.

Figs 63A–G , 64A–L , 65A–F , 66A–K

Nesticus reclusus Gertsch, 1984: 29, figs 75–78, 109–111.
Nesticus cooperi Gertsch, 1984: 30, figs 132–134, 144–146. syn. nov.

###### Material examined.

**Northeastern locations: Type material: *Holotype***: USA – **North Carolina, Swain Co.** • ♂ holotype; Andrew’s Bald, Great Smoky Mountains National Park; no date given; W.M. Barrows leg.; AMNH; **Non type material**: – **North Carolina, Swain Co.** • 8♂, 4♀; Great Smoky Mountains NP, Clingman’s Dome, vicinity Forney Ridge parking area; 35.5558°N, -83.496°W; 20 Aug. 1992; M. Hedin leg.; • 2♀; Great Smoky Mountains NP, Deep Creek, 0.25 mi. above Deep Creek CG, N Bryson City; 35.4644°N, -83.4344°W; 14 Aug. 1992; M. Hedin leg.; • ♀, 1 imm; Great Smoky Mountains NP, Hwy 441 E Thomas Ridge, 6.4 mi. N Smokemont CG turnoff; 35.6°N, -83.4091°W; 26 Aug. 2005; M. Hedin, R. Keith, J. Starrett, S. Thomas leg.; MCH 05_091; • 9♀; Great Smoky Mountains NP, Noland Creek at Laurel Branch, off Fontana Road, W of Bryson City; 35.4582°N, -83.5293°W; 26 Aug. 2005; M. Hedin, R. Keith, J. Starrett, S. Thomas leg.; MCH 05_093; • ♂; Hwy 129, NE of Cheoah Dam along Cheoah Reservoir; 35.4554°N, -83.9254°W; 17 Aug. 2007; M. Hedin, M. McCormack, S. Derkarabetian leg.; MCH 07_116; – **Tennessee, Sevier Co.** • ♂; Great Smoky Mountains NP, Elkmont Area; 35.6536°N, -83.5802°W; 31 Jul. 2000; M. Hedin, J. Cokendolpher leg.; MCH 00_144; • 2♂, 5♀; Great Smoky Mountains NP, Hwy 441 0.8 mi. N Newfound Gap; 35.62°N, -83.4197°W; 20 Aug. 1992; M. Hedin leg.; • ♂; Great Smoky Mountains NP, Lower Baskins Creek; 35.6957°N, -83.4823°W; B. Dellinger leg; • ♂; Great Smoky Mountains NP, N side of Mt Buckley, W of Clingman’s Dome; 35.5626°N, -83.5058°W; 21 Oct. 2012; M. Hedin, J. Bond, F. Coyle, S. Cameron leg.; MCH 12_039; • 3♀; Wear Cove, Myhr Cave; 2 Aug. 2000; M. Hedin, J. Cokendolpher, W. Reeves leg.; MCH 00_148; • 2♂, ♀; Wear Cove, Myhr Cave; 29 Aug. 2001; M. Hedin, M. Lowder, P. Paquin leg.; MCH 01_182.

**Southwestern locations**: USA – **North Carolina**: *Swain County*, Lost Nantahala Cave, near Nantahala, 17 May. 1979, coll. P.T. Hertl, S.P. Plantani, C.O. Holler (♂ holotype of *Nesticuscooperi*). – **Georgia, Gilmer Co.** • ♂, 2♀; Rock Creek Road, N of Rich Mountain Wilderness, 3 mi. E Cherry Log at Hwy 76; 34.7811°N, -84.3339°W; 15 Aug. 2007; M. Hedin, M. McCormack, S. Derkarabetian leg.; MCH 07_102; – **Georgia, Towns Co.** • 12♀, 3 imm; 180 spur to Brasstown Bald; 34.8593°N, -83.8008°W; 21 Aug. 2002; M. Hedin, M. Lowder, P. Paquin leg.; MCH 02_147; • 2♀; 180 spur to Brasstown Bald; 34.8593°N, -83.8008°W; 15 Aug. 2007; M. Hedin, M. McCormack, S. Derkarabetian leg.; MCH 07_104; – **North Carolina, Cherokee Co.** • 3♂, 4♀; Beaver Creek Road, along Beaver Creek, N of Andrews; 35.2152°N, -83.8327°W; 18 Aug. 2004; M. Hedin, R. Keith, J. Starrett, S. Thomas leg.; MCH 04_057; • ♂, 3♀; Junaluska Road along Junaluska Creek, SE of Andrews; 35.176°N, -83.768°W; 18 Aug. 2004; M. Hedin, R. Keith, J. Starrett, S. Thomas leg.; MCH 04_059; • ♂; Junaluska Road along Junaluska Creek, SE of Andrews; 35.176°N, -83.768°W; 18 Aug. 2007; M. Hedin, M. McCormack, S. Derkarabetian leg.; MCH 07_122; • 2♂, 4♀, 2 imm; Tatham Gap Road, S of Tatham Gap, N of Andrews; 35.2495°N, -83.8154°W; 18 Aug. 2004; M. Hedin, R. Keith, J. Starrett, S. Thomas leg.; MCH 04_058; • ♂, 2♀; Watkins Creek Road, off Hwy 19, SW of Topton; 35.2312°N, -83.7204°W; 19 Aug. 2004; M. Hedin, R. Keith, J. Starrett, S. Thomas leg.; MCH 04_066; – **North Carolina, Clay Co.** • ♂, 8♀, 3 imm; along Fires Creek, NE Omphus Ridge; 35.1099°N, -83.8267°W; 21 Aug. 2002; M. Hedin, F. Coyle, M. Lowder, P. Paquin leg.; MCH 02_145; • ♂, 4♀; Tusquitee Mountains, Fires Creek, Long Branch, just up from Short Branch; 35.1467°N, -83.7618°W; 21 Aug. 2002; M. Hedin, F. Coyle, M. Lowder, P. Paquin leg.; MCH 02_144; – **North Carolina, Graham Co.** • 3♂, 13♀, 11 imm; 0.25 mi. S Stecoah Gap on Appalachian Trail, off Hwy 143, Cheoah Mountains, NE of Cheoah; 35.353°N, -83.7187°W; 28 Aug. 2002; M. Hedin, M. Lowder, P. Paquin leg.; MCH 02_165; • 3♂, 8♀, 7 imm; along Panther Creek at Cook Branch confluence, N of Grassy Gap; 35.3677°N, -83.6272°W; 28 Aug. 2002; M. Hedin, M. Lowder, P. Paquin leg.; MCH 02_167; • ♀; Franks Creek, along Franks Creek Road, E of Sweetgum; 35.3158°N, -83.7361°W; 18 Aug. 2007; M. Hedin, M. McCormack, S. Derkarabetian leg.; MCH 07_121; • ♂, 2♀, 3 imm; Hwy 28, 0.6 mi. E entrance to Cable Cove campground; 35.4234°N, -83.7514°W; 28 Aug. 2002; M. Hedin, M. Lowder, P. Paquin leg.; MCH 02_166; • 3♂, 4♀; Hwy 28, ENE of Fontana Village, N side Yellow Creek Mountains; 35.4387°N, -83.8122°W; 18 Aug. 2007; M. Hedin, M. McCormack, S. Derkarabetian leg.; MCH 07_120; • 5♀; Panther Creek, FT 405; 35.3683°N, -83.6267°W; 18 Aug. 2007; M. Hedin, M. McCormack, S. Derkarabetian leg.; MCH 07_119; • ♂, 8♀, 4 imm; Snowbird Mountains, N Tatham Gap, head of Long Creek on FR 423; 35.2579°N, -83.8196°W; 27 Aug. 2002; M. Hedin, M. Lowder, P. Paquin leg.; MCH 02_162; • ♂, ♀; south of Stecoah Gap on Appalachian Trail, Cheoah Mountains, NE of Cheoah; 35.3546°N, -83.7186°W; 18 Jul. 1991; B. Dellinger leg.; – **North Carolina, Macon Co.** • 29♀, 10 imm; Ball Road, SE of Beechertown; 35.2687°N, -83.6672°W; 30 Aug. 2002; M. Hedin, M. Lowder, P. Paquin leg.; MCH 02_172; • ♂, 3♀; Ball Road, SE of Beechertown; 35.2687°N, -83.6672°W; 21 Aug. 2004; M. Hedin, R. Keith, J. Starrett, S. Thomas leg.; MCH 04_072; • 2♀; Jarrett Creek, W of Wayah Gap; 35.1587°N, -83.6349°W; 18 Aug. 2007; M. Hedin, M. McCormack, S. Derkarabetian leg.; MCH 07_123; • ♀; just N Jarrett Bald, above Wine Spring Creek; 35.1777°N, -83.6302°W; 1 May. 1993; B. Dellinger leg.; • 6♀; Nantahala River Gorge, SE of Hwy 74 19W, on Ball Road (also called Wayah Road); 35.2613°N, -83.6608°W; 10 Aug. 1992; M. Hedin leg.; • ♀; Nantahala River Gorge, vicinity Patton’s Run Overlook; 35.278°N, -83.681°W; 29 Aug. 2001; M. Hedin, M. Lowder, P. Paquin leg.; MCH 01_183; • 2♂, ♀, 8 imm; S Burnington Gap, head of Ben Creek; 35.2185°N, -83.5639°W; 30 Aug. 2002; M. Hedin, M. Lowder, P. Paquin leg.; MCH 02_170; • ♂, 2♀, 12 imm; S of Wayah Bald on FR 388, 0.9 mi. S Wayah Road; 35.1559°N, -83.5512°W; 30 Aug. 2002; M. Hedin, M. Lowder, P. Paquin leg.; MCH 02_169; • ♀; Wine Spring Creek, E Nantahala Lake off Wayah Bald Road, S of Aquone; 35.1913°N, -83.6381°W; 25 Mar. 1993; B. Dellinger leg.; – **North Carolina, Swain Co.** • 2♀; Nantahala River Gorge, 0.25 mi. downstream from Blowing Spring, Hwy 19W; 35.3307°N, -83.6272°W; 18 Apr. 1994; M. Hedin leg.; • ♂, 2♀; Nantahala River Gorge, E side of River along Hwy 19W, across from Talc Mountain quarry, NE of Hewitt; 35.312°N, -83.6406°W; 8 Apr. 1993; B. Dellinger leg.

###### Diagnosis.

Male palps of *Nesticusreclusus* are easily distinguished from close phylogenetic relatives *N.stupkai* and *N.bishopi*. In *N.reclusus* the distal tegular apophysis is shaped differently and has a blunt or forked tip, the space separating the distal from basal parts of the tegular apophysis is itself wide, and the median apophysis is shaped differently, with a spatulate basal end and a blade-like distal tip (Figs 63A–G, 64A–L). Females of these three species are challenging to diagnose; see comments above regarding the orientation of the dorsal epigynal plates.

###### Variation.

We here discuss and distinguish *Nesticusreclusus* populations as “northeastern” vs. “southwestern”, separated by the Little Tennessee River, including the Little Tennessee River Gorge and Fontana Lake (Fig. 53). The type locality for *N.reclusus* is in the northeast, at Andrew’s Bald in Great Smoky Mountains National Park. Southwestern populations surround and include the type locality of *N.cooperi* in the Nantahala River Gorge. We hypothesize that the Little Tennessee River might act as a dispersal barrier and promote divergence, although as discussed below combined evidence does not support this hypothesis.

In the northeast we examined males from seven locations in addition to the type locality, noting minimal palpal variation (Fig. 63A–G). One male from Lower Baskins Creek possessed a palp with a translucent bladelike paradistal process slightly wider at the base, and mostly lacking a ventromedial paracymbial process. Females from the northeast have conspicuously dark spermathecae and (viewed dorsally) the dorsal-projecting internal anterior plates are well sclerotized (Fig. 65A–F).

In the southwest we examined males from eighteen separate locations. All southwestern males approximated character conditions seen in northeastern males for all but one character. Males from eight locations possessed a paracymbium with the paradistal process lacking (and distomedial process moving towards the edge; Fig. 64I–L), like the condition seen in type *N.cooperi* (Gertsch 1984, figs 132–134). These locations included Fires Creek (MCH 02_144, MCH 02_145), Ben Creek (MCH 02_170), Wayah Bald (02_169), Ball Road (MCH 04_072), Panther Creek (02_167, 07_119), Junaluska Road (MCH 04_059, MCH 07_122) and Nantahala River Gorge (1993 collection, very near the type locality of *N.cooperi*). These locations are geographically contiguous, found mostly along the western flanks of the Nantahala Mountains including the Nantahala River Gorge (Fig. 53).

Females from southwestern populations vary slightly (Fig. 66A–K), but those from sample locations with “*N.cooperi*-like” males (Fig. 66I–K) are not obviously different from other populations. That is, we could not discern a distinctive “*N.cooperi*-like” female morphology.

###### Distribution and natural history.

This relatively wide-ranging montane species occurs from the northern side of the Great Smoky Mountains National Park, southwestward across the Little Tennessee River to the Yellow Creek, Cheoah, Snowbird, Nantahala, Valley River, and Tusquitee Mountains (Fig. 53). Two conspicuously disjunct populations occur even further south, in northern Georgia at Brasstown Bald and Rock Creek Road (Fig. 53). We comment more on the Rock Creek Road specimens below.

We hypothesize that the geographic gap north and northeast of Fontana Lake in the Great Smoky Mountains is an artifact of poor sampling, as this region is mostly roadless (Fig. 53). As such, northeastern vs. southwestern populations should be approximately contiguous, except for the river barrier itself. This differs from the situation in *Nesticusbishopi* versus *N.stupkai*, where we view the geographic disjunction as real (Fig. 53).

As an example of natural history, at Ball Road (MCH 02_172) a team collected 29 females and ten immatures in a 30-minute devoted survey from beneath rocks in a south-facing boulderfield. As mentioned above, *Nesticusreclusus* (♂, 4♀) was found in syntopy with *N.lowderi* (3♂, 5♀) at Fires Creek (MCH 02_144).

See comments above regarding the unlikely Gertsch (1984) record of *N.reclusus* from “*McDowell County, Montreat*”. Despite extensive collections we have never found members of the *reclusus* group from east of the Asheville Basin (Fig. 53).

###### Remarks.

Gertsch (1984) described both *Nesticusreclusus* and *N.cooperi*, distinguishing males by the shape of the basal tegular apophysis and the shape / presence of a paradistal paracymbial process (Gertsch referred to this as a dorsal process; see our comments above). Importantly, although Gertsch examined many records for montane *N.reclusus*, he only had *N.cooperi* specimens from two adjacent Nantahala River Gorge populations. Our geographic sampling has greatly expanded the distribution for southwestern *N.reclusus*, including many locations surrounding the type locality of *N.cooperi*. With this greater sampling we found that male morphology varies slightly with geography, particularly in the presence of the paradistal paracymbial process. We could not discern the shape differences in the basal tegular apophysis that Gertsch (1984) noted (Figs 63, 64). From a morphological perspective we view this as a single species with a relatively broad montane distribution, with minor male morphological variation across this distribution.

Nuclear phylogenomic data is mostly consistent with this single species hypothesis, except for the southern disjunct Rock Creek Road population, further discussed below. Only one “*Nesticuscooperi*-like” population was sampled for nuclear data (Nantahala River Gorge) and is embedded within a paraphyletic grade including both northeastern and other southwestern *N.reclusus* (Figs 3, 4). The nuclear data within this complex are notable for many low gene and site CF values, and low local posterior probability values (Figs 3, 4), suggesting extensive gene tree discordance.

The mitochondrial evidence is similarly challenging to interpret in this complex, as mitochondrial data do not support the larger *reclusus* group as monophyletic, and species interrelationships diverge strongly from that suggested by the nuclear data (Fig. 6). Within *Nesticusreclusus* itself Noland and Clingmans sequences are recovered with *N.stupkai* sequences, separate from Smokemont and Newfound sequences. We hypothesize that this discordance is a result of mitochondrial introgression from *N.stupkai* into certain *N.reclusus* populations, where these taxa occur in geographic proximity. For example, sympatry in Myhr Cave is a potential conduit for mitochondrial gene exchange. Six sampled locations with a “*N.cooperi*-like” paracymbium do not form a clade on mitochondrial trees (Fig. 6).

The southern disjunct Rock Creek Road sample (Fig. 53) adds further intrigue to this complex. Mitochondrial sequences are highly divergent, falling with *Nesticussheari* (Fig. 6), while nuclear sequences are sister to a clade including *N.stupkai*, *N.bishopi*, and remaining *N.reclusus* (Figs 3, 4). At the same time, males from this location possess unremarkable palps, identical in detail to other southwestern *N.reclusus* palps (Fig. 64E, F), and females are similarly morphologically unremarkable (Fig. 66G, H). We suspect that gene flow across species boundaries (perhaps involving *N.sheari*?) might be impacting results in this part of the *Nesticus* phylogeny. More geographic and UCE sampling in this geographic region will be needed to resolve this tricky taxonomic issue.

## Supplementary Material

XML Treatment for Nesticus stygius

XML Treatment for Nesticus cressleri

XML Treatment for Nesticus jemisinae

XML Treatment for Nesticus archeri

XML Treatment for Nesticus pecki

XML Treatment for Nesticus silvanus

XML Treatment for Nesticus cherokeensis

XML Treatment for Nesticus holsingeri

XML Treatment for Nesticus mimus

XML Treatment for Nesticus tennesseensis

XML Treatment for Nesticus dilutus

XML Treatment for Nesticus carolinensis

XML Treatment for Nesticus paynei

XML Treatment for Nesticus roanensis

XML Treatment for Nesticus nasicus

XML Treatment for Nesticus brimleyi

XML Treatment for Nesticus templetoni

XML Treatment for Nesticus crosbyi

XML Treatment for Nesticus gertschi

XML Treatment for Nesticus secretus

XML Treatment for Nesticus canei

XML Treatment for Nesticus bondi

XML Treatment for Nesticus barrowsi

XML Treatment for Nesticus lowderi

XML Treatment for Nesticus barri

XML Treatment for Nesticus furtivus

XML Treatment for Nesticus carteri

XML Treatment for Nesticus georgia

XML Treatment for Nesticus lula

XML Treatment for Nesticus sheari

XML Treatment for Nesticus dellingeri

XML Treatment for Nesticus jonesi

XML Treatment for Nesticus binfordae

XML Treatment for Nesticus dykemanae

XML Treatment for Nesticus bishopi

XML Treatment for Nesticus stupkai

XML Treatment for Nesticus reclusus

## Supplementary materials

Supplementary material 1 *Nesticus* morphological material examined Data type occurrences (excel file) https://binary.pensoft.net/file/804518This dataset is made available under the Open Database License (http://opendatacommons.org/licenses/odbl/1.0/). The Open Database License (ODbL) is a license agreement intended to allow users to freely share, modify, and use this Dataset while maintaining this same freedom for others, provided that the original source and author(s) are credited.Marshal Hedin, Marc A. Milne

Supplementary material 2 *Nesticus* immature specimen records Data type occurrences (excel file) https://binary.pensoft.net/file/804519This dataset is made available under the Open Database License (http://opendatacommons.org/licenses/odbl/1.0/). The Open Database License (ODbL) is a license agreement intended to allow users to freely share, modify, and use this Dataset while maintaining this same freedom for others, provided that the original source and author(s) are credited.Marshal Hedin, Marc A. Milne

Supplementary material 3 Specimens used in molecular work Data type DNA records (excel file) https://binary.pensoft.net/file/804520This dataset is made available under the Open Database License (http://opendatacommons.org/licenses/odbl/1.0/). The Open Database License (ODbL) is a license agreement intended to allow users to freely share, modify, and use this Dataset while maintaining this same freedom for others, provided that the original source and author(s) are credited.Marshal Hedin, Marc A. Milne

Supplementary material 4 Input alignments, analysis log files, and output tree files Data type zip. archive https://binary.pensoft.net/file/804521This dataset is made available under the Open Database License (http://opendatacommons.org/licenses/odbl/1.0/). The Open Database License (ODbL) is a license agreement intended to allow users to freely share, modify, and use this Dataset while maintaining this same freedom for others, provided that the original source and author(s) are credited.Marshal Hedin, Marc A. Milne
